# Finishing a complete giraffe genome from telomere to telomere with Verkko-Fillet

**DOI:** 10.1016/j.xgen.2026.101279

**Published:** 2026-08-06

**Authors:** Juhyun Kim, Benjamin D. Rosen, Sarah E. Fumagalli, Kristen L. Kuhn, Amy Long, Jeffrey J. Schoenebeck, Heather Schwartz, Lan Wu-Cavener, Aleksey V. Zimin, Douglas R. Cavener, Timothy P.L. Smith, Adam M. Phillippy, Sergey Koren, Arang Rhie

**Affiliations:** 1 Center for Genomics and Data Science Research, National Human Genome Research Institute, National Institutes of Health, Bethesda, MD 20892, USA; 2 Animal Genomics and Improvement Laboratory, USDA-ARS, Beltsville, MD 20705, USA; 3 Meat Animal Research Center, USDA-ARS, Clay Center, NE 68933, USA; 4 Cincinnati Zoo and Botanical Garden, Cincinnati, OH 45220, USA; 5 The Roslin Institute and Royal (Dick) School of Veterinary Studies, Midlothian EH25 9RG, UK; 6 Nashville Zoo, Nashville, TN 37211, USA; 7 Department of Biology, Pennsylvania State University, University Park, PA 16802, USA; 8 Department of Biomedical Engineering, Johns Hopkins University, Baltimore, MD 21218, USA; 9 Departments of Computer Science, Johns Hopkins University, Baltimore, MD 21218, USA; 10 Department of Genetic Medicine, Johns Hopkins University School of Medicine, Baltimore, MD 21205, USA

**Keywords:** genome assembly curation, Telomere-to-Telomere (T2T) genome assembly, giraffe reference, assembly graph, reference genomes, diploid assembly, graph-based curation, haplotype-resolved assembly, complete genome assembly, genome assembly refinement

## Abstract

High-quality reference genomes are critical for studying the biology of the genome, but current methods often leave gaps and errors, especially in repetitive regions. These issues arise from challenges in genome graph curation and are not fully resolvable by standard polishing approaches. To address this, we developed Verkko-Fillet, a Python-based interactive framework for inspecting, editing, and refining genome assembly graphs. It integrates multiple data sources and provides tools for visualization, gap filling, and structural correction. Applied to a giraffe and the benchmark human genome, Verkko-Fillet improves a draft assembly (Q61.5) to a complete telomere-to-telomere genome (Q73.6), increasing both contiguity and accuracy. This work highlights the importance of graph-based curation for producing a finished, gapless genome assembly suitable for downstream analyses.

## Highlights

• Verkko-Fillet enables interactive curation of complex genome assembly graphs
• Improved path continuity increases genome assembly accuracy and quality
• A diploid T2T giraffe genome resolves major reference errors

## Graphical abstract

**Figure:**
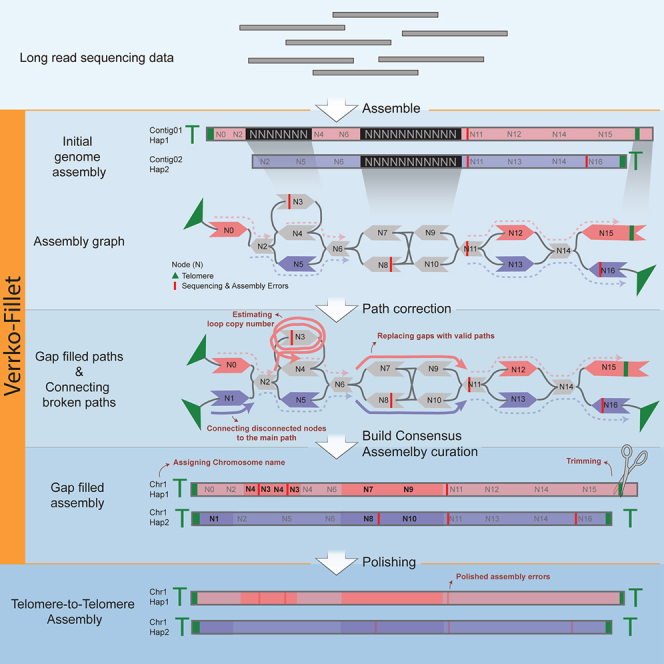

## Teaser

Kim et al. developed Verkko-Fillet, a Python-based interactive framework for genome graph path inspection, editing, and curation that improves assembly quality. Applying this tool to a draft giraffe genome generated a high-quality diploid telomere-to-telomere reference genome and corrected large assembly errors present in the previous reference. The improved assembly enabled high-quality gene annotations, which were produced alongside the genome for downstream analysis.

## Introduction

Generating high-quality genome assemblies is a critical foundation for understanding both individual species and their evolutionary relationships across the tree of life. A reference genome with minimal errors enables accurate functional annotation, comparative genomics, and evolutionary inference. To this end, numerous international efforts—such as the Telomere-to-Telomere (T2T) Consortium,^1^^,^^2^^,^^3^ the Human Pangenome Reference Consortium (HPRC),^4^^,^^5^ Vertebrate Genomes Project (VGP),^6^ and Ruminant Telomere-to-Telomere (RT2T) Consortium^7^—focused on producing complete and accurate genome assemblies. These initiatives have driven the development of a wide range of genome assembly^8^^,^^9^^,^^10^^,^^11^ and polishing algorithms^12^^,^^13^^,^^14^ aimed at improving assembly continuity, correctness, and completeness.

Traditional *de novo* assembly methods relied on scaffolding consecutive sequences with no gaps (contigs) assembled from long reads into linear haploid sequences.^15^^,^^16^^,^^17^^,^^18^^,^^19^ However, approaches have shifted toward graph-based assembly, allowing for more accurate representation of complex genomic regions by integrating linked-read, Hi-C, or phasing information using parental genomes.^8^^,^^10^^,^^11^^,^^20^^,^^21^^,^^22^^,^^23^^,^^24^ These tools enable accurate reconstruction of diploid^4^^,^^25^^,^^26^^,^^27^^,^^28^ and polyploid genomes,^29^^,^^30^^,^^31^ and even precise haplotype phasing, avoiding the collapse of multiple haplotypes into a single consensus sequence (a limitation often inherent to linear assembly models).

Despite advances in long-read sequencing and assembly algorithms, genomes still contain gaps and haplotype-switch errors, often caused by sequencing dropouts and repetitive regions,^32^^,^^33^ particularly within rDNA clusters and centromeres.^34^^,^^35^ While read mapping-based polishing methods have been used in the past to fill or extend sequences into the gap^36^^,^^37^^,^^38^ and correct assembly errors,^14^^,^^39^^,^^40^ they largely depend on the quality of the backbone assembly and are ineffective at resolving large, misassembled regions.^6^ Addressing structural issues before polishing is therefore critical, but manual graph editing and rebuilding assemblies remain technically challenging and difficult to be reproduced systematically.

To address this challenge, we developed Verkko-Fillet, a Python-based interactive framework for genome assembly graph curation and gap resolution, designed for use alongside the Verkko assembler. Here, we applied Verkko-Fillet to generate a complete “telomere-to-telomere” (T2T) reference genome of the giraffe (*Giraffa tippelskirchi*), demonstrating each step of the gap-filling and curation process. This approach achieved substantial improvements in base-level accuracy (QV), genome completeness, and alignment quality, ultimately producing a gapless diploid genome in which all contigs represent chromosomal haplotypes. The giraffe T2T assembly is more accurate than existing references, correcting major assembly errors and better reflecting known biological and cytogenetic features. When refining a draft Verkko assembly of the benchmark human genome (T2T-HG002),^26^ we confirmed assembly quality improved, matching closely to the benchmark genome. This underscores the utility of combining Verkko with Verkko-Fillet to generate biologically accurate and complete T2T reference genomes.

## Design

**Figure 1:**
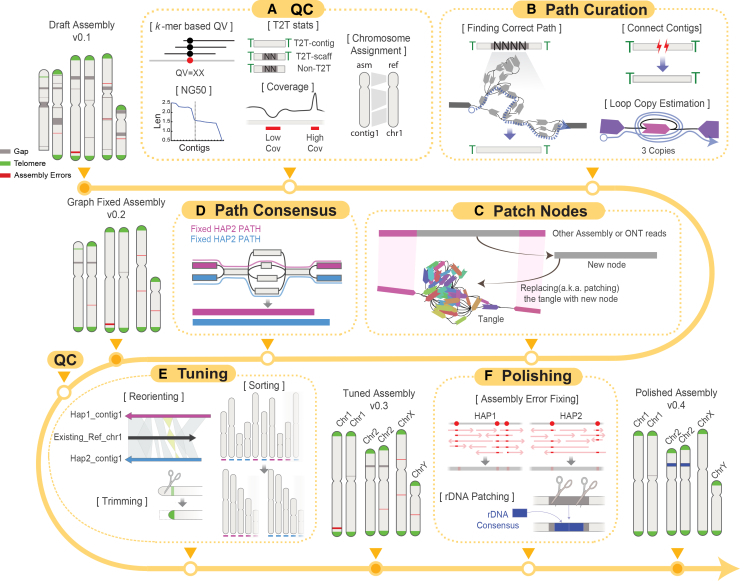
Verkko-Fillet is a software framework for the analysis and curation of complete genome assemblies (A) The quality control (QC) steps. (B) Gap filling and connecting broken contigs. (C) Unresolved repeat nodes in the graph are patched with ONT reads or nodes generated with an alternate assembler. (D) Verkko regenerates the consensus assembly using the curated paths. (E) Tuning step applies contig-level modifications and chromosome assignment. (F) Optional polishing step corrects base-level errors and rDNA patches, producing a finished T2T assembly.

Verkko-Fillet refines genome assemblies using the assembly graph and paths, providing interactive guidance for curation. The workflow (Figure 1) consists of the following steps: (1) assembly quality control (QC), (2) path curation and node patching, (3) path consensus generation, (4) QC of the updated assembly, and (5) chromosome assignment and tuning, with optional polishing. A key feature of Verkko-Fillet is path correction, enabling graph-based gap closing. By integrating outputs from Verkko, GraphAligner, and Merqury, Verkko-Fillet provides actionable insights to resolve gaps and ambiguities in assembly paths. Verkko-Fillet utilizes Oxford Nanopore Technologies (ONT) ultra-long reads spanning gap-flanking nodes and Hi-C to resolve ambiguous haplotype assignment. Verkko-Fillet retrieves and presents this information in clear, user-friendly tables for easy interpretation. The framework is organized into three modules—preprocessing (pp), tools (tl), and plotting (pl)—offering specialized functions during the refinement process.

## Results

### An interactive framework for generating T2T contigs

The Verkko assembler outputs multiple files, including assembly graphs, homopolymer-compressed nodes, stitched DNA segments (“pieces”), paths, and contigs (Figure S1). Additionally, Hi-C or trio-binning strategies produce useful information for scaffolding and haplotype phasing. Effectively and seamlessly integrating these heterogeneous data types is essential for gap-filling and path refinement. Verkko-Fillet provides a unified framework to oversee the entire refinement process, from parsing the initial Verkko outputs to producing a gap-resolved assembly that is ready for polishing.

We developed a Verkko-Fillet object (hereafter referred to as “vf-obj”), a Python class designed to read and organize all output formats from Verkko into a single object using attribute-based methods (Figure S2). The vf-obj maintains relationships among different data types in a manner analogous to a relational database, facilitating efficient data retrieval and analysis. The primary datasets handled by vf-obj are the assembly graph and paths. The graph consists of nodes and edges, while paths define the linear order and orientation of nodes corresponding to each contig. During gap-filling steps, vf-obj stores the name and orientation of nodes used to close assembly gaps and subsequently writes corrected path files for rerunning Verkko’s consensus module to generate an updated, gap-resolved assembly.

It is recommended to evaluate the quality of the assembly prior to path curation (Figure 1A). Verkko-Fillet provides functions to calculate QV by detecting error *k*-mers in the assembly, assessing the proportion of detectable telomeric motifs at contig ends, and identifying the number and locations of gaps, enabling classification of assembly completeness. A chromosome is considered a “T2T-contig” if both telomeres are present with no gaps, a “T2T-scaffold” if both telomeres are present but a few internal gaps remain, and just a “scaffold” or "non-T2T" if one or both telomeres are missing. These QC steps are recommended each time a new assembly is generated.

The core gap-filling function of Verkko-Fillet leverages ONT ultra-long reads mapped to the assembly graph with GraphAligner.^41^ Reads spanning the flanking nodes of unresolved gaps are identified and used to infer the correct paths through the graph, enabling replacement of missing regions. This process includes copy number estimation of repeats within loops and reconnection of previously disconnected scaffolds (Figure 1B). It can replace a graph tangle with a single bridging node constructed from sufficiently long ONT reads or long contigs generated by other assemblers (e.g., hifiasm) (Figure 1C). As this step introduces a new node that is absent from the original assembly graph, it requires a high degree of confidence in the accuracy of the supporting sequence, because tangles are formed by repetitive sequences that are inheritently difficult to resolve. Once the new paths are constructed, they are subsequently used to regenerate consensus sequences (Figure 1D). Throughout the process, Verkko-Fillet supports Integrative Genomics Viewer (IGV)^42^ and Bandage^43^ compatible intermediate files, facilitating simultaneous inspection of the assembly graph and sequence-level features.

The structurally refined assembly then undergoes tuning and polishing. The tuning step involves reorienting scaffolds according to known chromosomal orientation (if available), trimming, and sorting by chromosome or haplotype (Figure 1E). For species with no prior reference, Verkko-Fillet provides an option to sort the chromosomes by length and orient them based on user-provided centromere information. A finalized high-quality T2T assembly can then be generated by following the polishing workflow provided in a tutorial using error *k*-mers and variant-calling methods (Figure 1F). Because rDNA regions are notoriously difficult to assemble due to their high copy number, rDNAs are often partially resolved with gaps. When no intact rDNA copies are present at the gap boundary, Verkko-Fillet offers an option to insert representative rDNA copies generated with Ribotin^44^ (Figure 1F).

**Figure 2:**
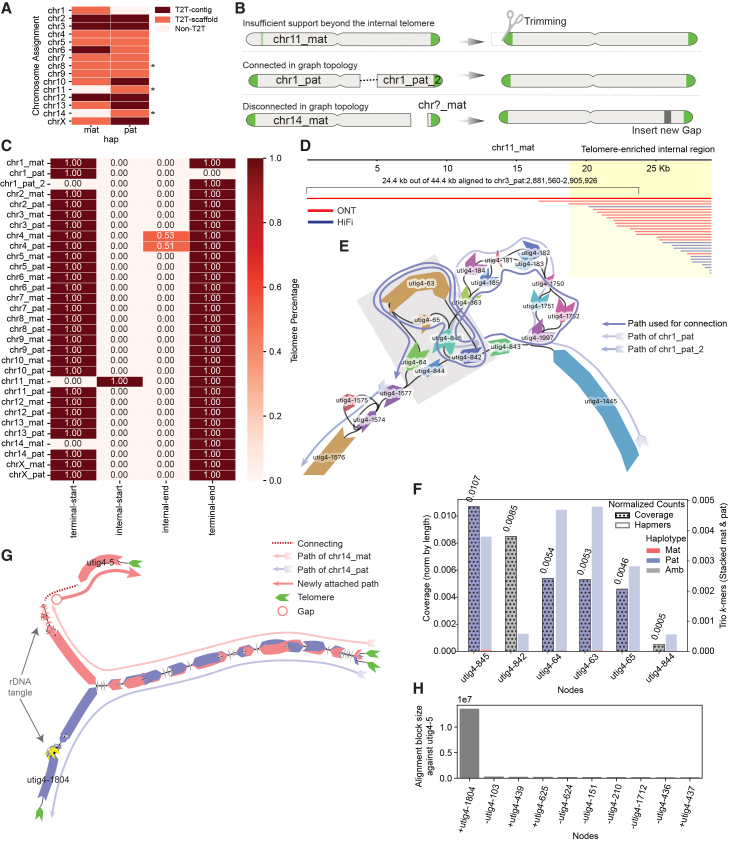
Recovering T2T contigs (A) Heatmap of telomere-to-telomere (T2T) status for each chromosome in the T2T-mGirTip1v0.1 assembly. Asterisks (∗) denote contigs containing rDNA arrays (see also Figure S4). (B) Schematic representation of three categories of non-T2T scaffolds. (C) Maximum proportion of telomeric sequence per scaffold, distinguishing terminal (≤15 kb from ends) and internal (>15 kb) regions. (D) Sequencing read composition at the tip node of maternal chr11 in the T2T-mGirTip1v0.1 assembly. The yellow shaded box highlights internal regions containing canonical telomeric repeats. (E) Assembly graph near a break in the paternal chr1 scaffold. (F) Normalized coverage and haplotype-specific *k*-mer (hapmer) counts per node length surrounding the breakpoint in €, highlighted by the gray box. (G) Graph structure of chr14 from both haplotypes. (H) Pairwise alignment of the disconnected node *utig4-5* in (G) against all other nodes in the graph, ranked by relative alignment block length.

Here, we present each step of the assembly refinement process using the genome of a male giraffe as a case study (Figure S3). The giraffe genome consists of 14 autosomes and the X and Y sex chromosomes, totaling 30 diploid chromosomes, along with mitochondrial DNA. Cytogenetically, all chromosome pairs except chromosomes 14 and Y are metacentric chromosomes, while chromosomes 14 and Y are acrocentric.^45^ The initial draft assembly (T2T-mGirTip1v0.1) was generated using Verkko v2.2 with parental Illumina short reads to support trio-binning, followed by haplotype extension using Hi-C data.^10^ The initial assembly graph comprises 2,755 nodes and 3,679 edges, representing a total diploid genome size of 3.40 Gb. This graph-based assembly consists of 10 T2T-contigs, 17 T2T-scaffolds, and 3 scaffolds classified as non-T2T (Figures 2A and S4).

### Recovering missing telomere ends

There are three common scenarios in which a contig fails to meet the criteria to be classified as T2T (Figure 2B). First, misplaced reads at the contig tip may introduce additional non-telomeric sequence. Second, a contig path may become disrupted within a complex repeat tangle, resulting in its fragmentation into two separate contigs, even though the graph remains fully connected. Third, a contig may lack a terminal node, leaving the end disconnected in the graph topology. All three scenarios are amenable to correction through tuning and graph-based refinement, enabling recovery of complete T2T contigs.

As an example, we detected an anomalous pattern in the maternal contig of chr11 (Figure 2C) when analyzing the distribution of telomeric motifs across contig termini. A high density of telomeric repeats was observed ∼18.8 kb from the 5′ end of the contig, while the true terminal sequence showed no detectable telomeric signal. Examination of read support at the 5′ terminus revealed that this non-telomeric segment was derived from one ONT read, whereas the internal region enriched for telomeric repeats was consistently supported by both ONT and HiFi datasets (Figure 2D). When we re-aligned the ONT read to the assembly, we found that the extra segment fully mapped to chr3_mat at 2.88–2.90 Mb, indicating that the segment was attached from an erroneous chimeric ONT read was often found in ligation-based methods.^46^ To recover a true T2T chromosome, the contig was trimmed to the point where a validated telomeric signal was detected during the tuning step, after the consensus step to close gaps as determined in the “gap filling” section below (Figure 1E).

We identified two large paternal haplotype scaffolds, both assigned to chr1, with one containing the p-arm telomere and the other the q-arm telomere (Figures 2C and 2E). These scaffolds should form a single continuous sequence but were separated by a large, complex tangle, presenting two possible traversal nodes, only one of which should be used: utig4-64 or utig4-844. To resolve this ambiguity, we evaluated trio-specific *k*-mer counts and ONT read coverage for each node within the tangle and found that utig4-64 had a higher number of paternal *k*-mers and greater ONT coverage than utig4-844, indicating that it more likely represents the true haplotype path (Figure 2F). Based on this evidence, we first identified the node within the large loop and then inferred the most plausible traversal as (utig4-842 → utig4-63 → utig4-64 → utig4-1577), thereby connecting the two paths into a continuous paternal chr1 (chr1_pat) T2T-contig.

Maternal chr14 (chr14_mat) was also missing one telomere (Figure 2C). Unlike the chr1_pat example, the chr14_mat graph lacked the telomeric node beyond the rDNA tangle (Figure 2G). However, we identified an unconnected node (utig4-5) with a length comparable to the telomere-bearing node at the end of the homologous paternal short arm (utig4-1804). To determine whether utig4-5 belonged to maternal chr14, we aligned it against all other nodes in the graph and ranked the results by alignment block length. The top match was utig4-1804, indicating that utig4-5 represents the missing maternal telomeric node (Figure 2H). Because no edges connected utig4-5 to the main contig, we bridged the inferred path with a gap placeholder, yielding the structure: utig4-5 → GAP → utig4-436. With this adjustment, the contig now qualifies as a T2T-scaffold.

### Gap filling

Gaps in genome assemblies can arise from multiple sources and can be broadly classified into two major categories, graph tangles and unassigned haplotypes (Figure S5). Tangles occur when nodes in the assembly graph are interconnected in a complex manner, making it difficult to resolve a clear, haplotype-specific path. These typically occur in repetitive regions of the genome, such as recently duplicated gene families or satellite repeat arrays.

**Figure 3:**
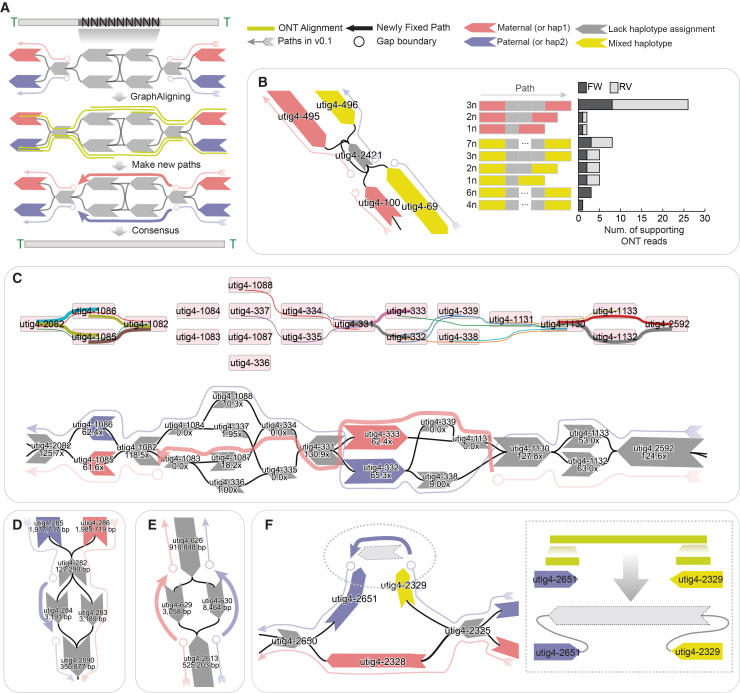
Generating a gapless assembly (A) Schematic view of gap filling using ONT alignments to the assembly graph. (B) Example of copy number estimation for a repeat node. Stacked bar chart shows ONT reads, supporting different path configurations, stratified by mapping direction. (C) Example of a complex tangle in the graph in which ONT alignments span multiple nodes. The depth in the bottom Bandage plot represents HiFi coverage from assembly graph construction. Some nodes show zero coverage due to repeats, missing HiFi reads, or reliance on ONT reads. Nodes with low (but nonzero) HiFi coverage likely reflect split coverage from genomic variation, such as cell heterogeneity or somatic changes. (D) Example of haplotype assignment by inference from the path of the counterpart haplotype. (E) Example of an ambiguous case where haplotypes are assigned randomly. (F) Patching example. A missing connection between two nodes on the paternal haplotype was resolved by generating a new patch node derived from split ONT alignments.

Gaps in the giraffe genome were resolved by integrating multiple sources of supporting data. The primary dataset consists of ONT read alignments to the assembly graph (Figure 3A). Specifically, we re-aligned the ONT reads used in the initial assembly to the graph using GraphAligner,^41^ enabling the identification of reads that span the flanking nodes on either side of a gap, as well as node-level coverage profiles. These spanning reads provide strong evidence for the correct traversal through unresolved regions. Additional data sources, including Hi-C read alignments and trio-specific *k*-mer markers, were also employed when available to further support gap resolution and facilitate accurate haplotype assignment.

Loop resolution is straightforward when sufficiently long ONT reads span the haplotype-specific flanking nodes surrounding the loop, supporting the estimation of the number of repeat units traversed by each read (Figure 3B). In the maternal chr11 haplotype around rDNA tangle, the majority of reads span from utig4-495 to utig4-100, each traversing three copies of utig4-2421 (21 kb), a length readily covered by ONT reads. In contrast, in the paternal haplotype, eight supporting reads span from utig4-69 to utig4-496, traversing seven copies of utig4-2421, with most alignments supporting this configuration. The alignment orientations are balanced between forward and reverse strands. Based on these estimates, we reconstructed the loop by inserting the corresponding number of repeat nodes into each haplotype-specific path.

We resolved the path in more complex tangles by integrating coverage and ONT alignment information. In one case, the diploid graph diverged into four nodes within the same genomic region, which may be associated with HiFi read dropout or somatic variations (Figure 3C). ONT alignments were detected only once each on utig4-1088 and utig4-334, respectively, but HiFi coverage indicated substantially higher support for utig4-1088 and utig4-1087, both of which were ultimately included in the resolved paths. Consequently, utig4-1087 was incorporated into the maternal path to fully resolve the tangle, as utig4-1088 had already been used in the paternal path.

Each path in the assembly graph is highly specific to its corresponding haplotype. Consequently, gaps may arise in regions where it is difficult to select a haplotype-specific node within a simple bubble consisting of two nodes (Figures 3D and 3E). In such cases, the gap can often be resolved by incorporating a node that is unused by the other haplotype (Figure 3D). However, when gaps occur in both haplotypes due to ambiguity between nodes, a last-resort approach is to fill the gap by assigning a path arbitrarily. This can improve contiguity and completeness, albeit at the cost of potentially introducing a haplotype switch error (Figure 3E).

Gaps may also arise from missing nodes or edges in the assembly graph, often due to insufficient HiFi coverage to make nodes or lack of support from ONT long reads to make edges (Figure 3F). In this case, the graph was disconnected, which prevented ONT alignments from spanning both nodes in a single path. However, we identified the ONT reads that aligned uniquely to both nodes in a split manner, allowing us to reconstruct the missing sequence. This process required introducing a new node and edges to connect the flanking nodes, thereby restoring graph continuity, and the new node is then used during the consensus step to fill the gap.

Interestingly, each rDNA locus shows a haplotype-specific tangle that is relatively small and can be resolved using the ONT alignments (Figures 3B and S6). However, chromosome 14 exhibits a larger and more complex tangle. In the maternal haplotype, three distinct small tangles are observed, allowing the paths to be inferred using ONT alignments. In contrast, the paternal haplotype contains a single large rDNA tangle composed of many nodes, with a central node surrounded by looping nodes, which cannot be fully resolved using the ONT data. Therefore, we inferred the paths based on ONT alignments as much as possible, while the remaining segments were selected according to their expected coverage, with lower confidence in node ordering. This approach may introduce assembly errors if incorrect edges are chosen during path reconstruction. Therefore, this array is considered less reliable than the other assembled arrays.

In summary, starting from T2T-mGirTip1v0.1 with 284 initial paths, we generated the T2T-mGirTip1v0.2 assembly using Verkko-Fillet, retaining only 31 essential paths—28 autosomes, two sex chromosomes, and one mitochondrial chromosome. Of the 45 gaps identified in the initial assembly, we successfully filled all but one, introducing a single new gap to reconstruct a fully telomere-capped scaffold for chr14_mat (Figure 2G).

### Trimming, chromosome assignment, and polishing

Consensus sequence generation (Figure 1D) for T2T-mGirTip1v0.2 was followed by tuning steps including trimming, reorientation (flipping), renaming, and sorting (Figure 1E). chr11_mat was the only contig missing one telomeric end (Figure S7). As noted above, the chr11_mat contig required specific trimming during this tuning stage because the tip sequence was derived from the consensus of a single ONT read (Figure 2D).

**Figure 4:**
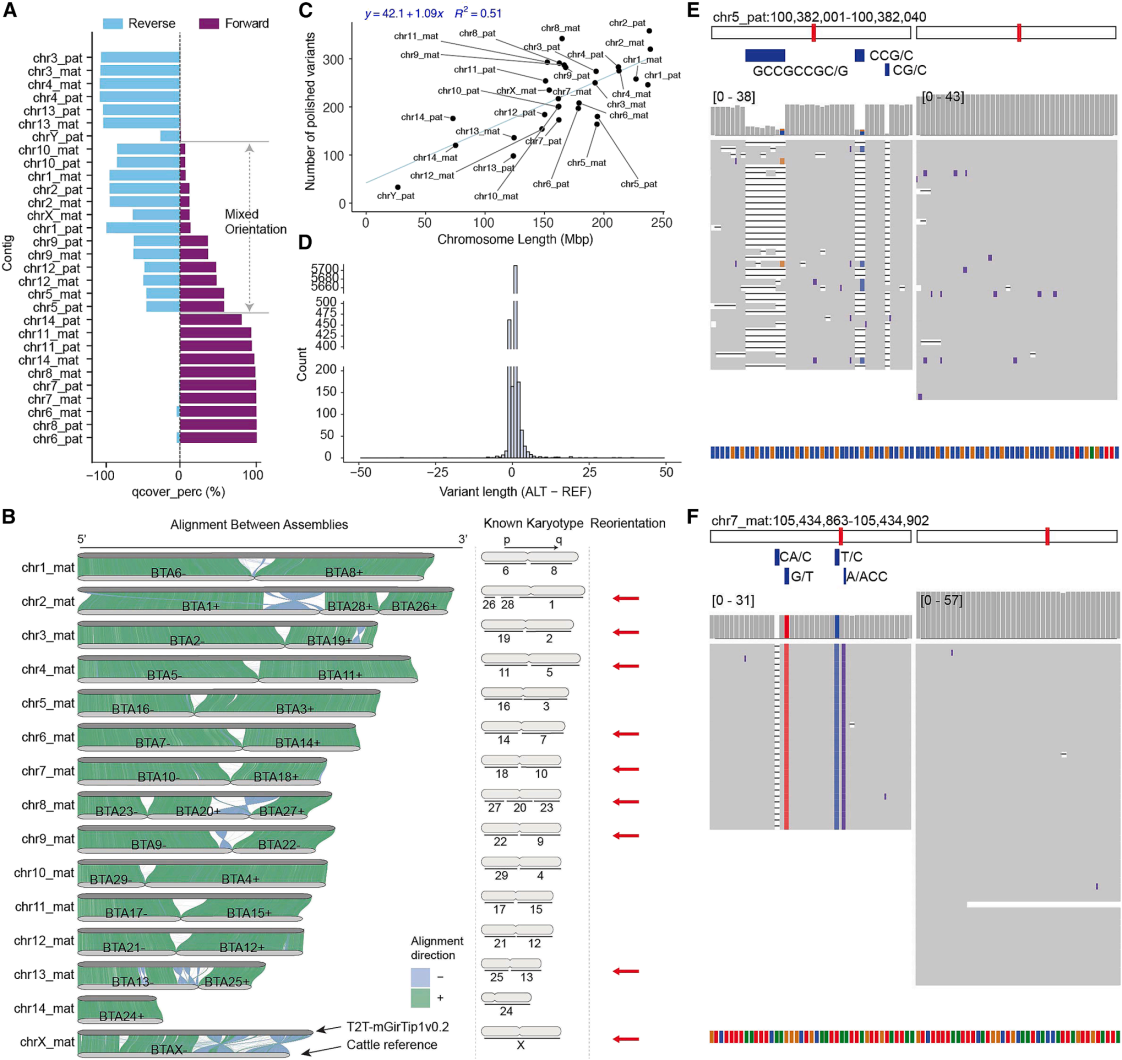
Tuning and polishing of the T2T-mGirTip1v0.2 assembly (A) Alignment size and strand orientation of the query assembly (T2T-mGirTip1v0.2) against the species-matched reference (ASM1759144v1) for chromosome assignment and orientation verification. (B) Alignment of the cattle reference (ARS-UCD2.0, Bos taurus) to T2T-mGirTip1v0.2. Upper: T2T-mGirTip1v0.2-Mat contigs and lower: cattle chromosomes. Contigs requiring reorientation are indicated by red arrows in the “reorientation” column. (C) Number of polished variants per chromosome as a function of contig length. The regression line is shown as y = 42.1 + 1.09×; R^2^ = 0.51. (D) Histogram of polished variants by size. (E and F) Examples of the same genomic region before (left) and after polishing (right), showing HiFi alignments with mismatches colored as A (green), C (blue), G (orange), and T (red). See also Figure S8.

Each contig was renamed according to its chromosomal assignment and haplotype, based on alignments to a matched reference genome when available. Using the available giraffe reference (GenBank: ASM1759144v1), we assigned contigs to chromosomes based on sufficiently long alignment blocks (Figure 4A). However, approximately one-third of the sequences could not be reliably oriented because the alignments contained mixed directionalities (Figure 4A). We leveraged the conventionally used comparative karyotype of giraffe to cattle^47^^,^^48^ to resolve the orientation by aligning the cattle reference genome (*Bos taurus* [BTA], RefSeq: GCF_002263795.3) to T2T-mGirTip1v0.2 (Figure 4B). For example, the known cattle-giraffe homology for giraffe chr1 is BTA6(−) followed by BTA8(+), which is consistent with the alignment of T2T-mGirTip1v0.2. In contrast, cattle BTA1, BTA28, and BTA26 aligned in the opposite order on T2T-mGirTip1v0.2 compared with the established recombination karyotype, prompting us to flip this contig. Overall, 9 of the 15 contigs per haplotype required reorientation to conform to the widely accepted giraffe karyotype. Finally, the contigs were sorted by chromosome name and haplotype.

Base-level correction of the assembly (Figure 1F) was performed using a variant-based strategy described by McCartney et al.^14^ and Yoo et al.^49^ In total, 6,689 correctable assembly errors were identified (excluding those on chrM), corresponding to an average of 1.3 variants per Mb (Figure 4C). The majority of corrections were 1 bp insertions (Figure 4D). Polishing improved local read alignments, reducing mismatches and indels within the corrected regions (Figures 4E and 4F), and further confirmed that reads that were incorrectly aligned in the unpolished assembly were mapped to the other haplotype with low mapping quality (Figure S8).

The current constraints of ultra-long read length and accuracy limit the ability to resolve complex rDNA arrays for some species’ assemblies. Verkko-Fillet provides an option to replace rDNA gaps with a model rDNA sequence (morph), in the form of gap – rDNA units – gap, when rDNA sequences are absent from the main chromosomes, thereby incorporating the most prevalent rDNA variants into the assembly (Figure S9). This conservative approach enhances the assembly by minimally preserving chromosome-specific rDNA unit information while avoiding the introduction of assembly errors created by inaccurate node traversals. In T2T-mGirTip1v0.2, all gaps, including those within rDNA tangles, were resolved without the need for this replacement. However, one rDNA unit on chr14_pat still showed a high density of local error *k*-mers after polishing, resulting from uncertain node ordering during gap filling. Thus, this one unit was removed (Figure S10).

Finally, the mitochondrial genome was circularized, and pseudoautosomal regions (PARs) were identified on chrX and chrY. All these post-polishing steps were carried out using Verkko-Fillet modules.

### Assembly quality evaluation for structural and base-level accuracy

**Figure 5:**
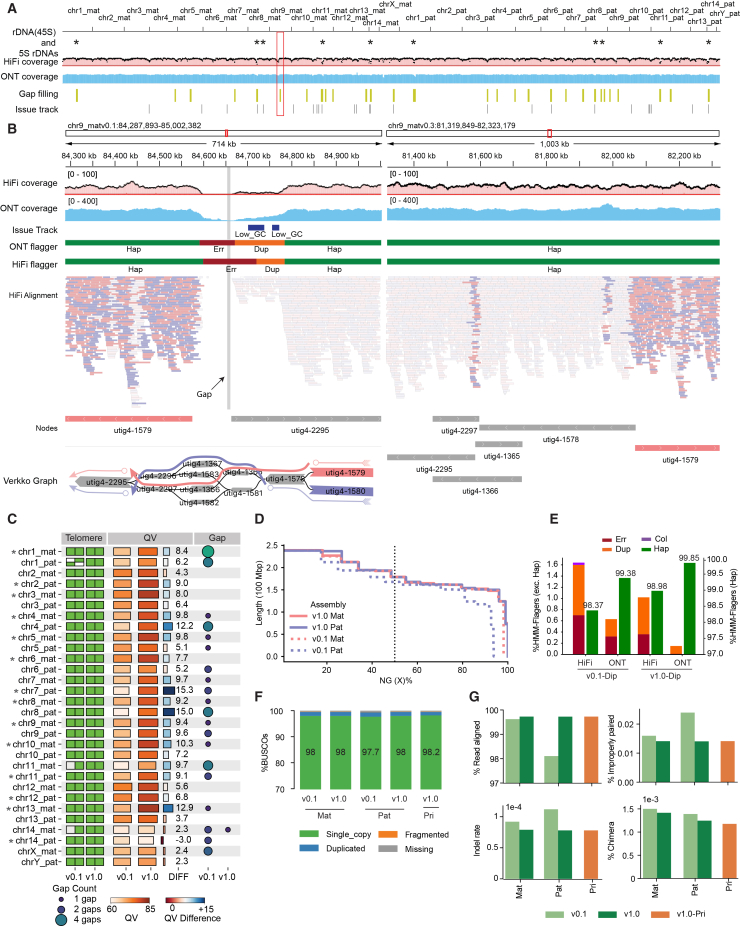
Structural and base-level refinement of the T2T-mGirTip1 assembly using Verkko-Fillet (A) Overview of the alignments and statistics for the T2T-mGirTip1v0.3 gap-filled and tuned assembly. The HiFi coverage track is overlaid with the first allele (red dots) and second allele (black dots) from NucFlag.^50^ Gap-filled regions are shown on the “gap filling” track. The “issue” track highlights regions identified by error *k*-mers and abnormal HiFi and ONT coverage. (B) A zoomed-in region before (left) and after (right) gap-filling on chr9_mat, highlighted by a red box in (A). Coverage abnormalities classified by hmm-flagger are shown below. HiFi alignments are colored by strand or shaded to indicate MAPQ = 0. Node sequence alignment from the original graph and the reconstructed paths are shown at the bottom. Note that chr9 was among the contigs re-oriented during the tuning step (Figure 4B). (C) Summary metrics comparing T2T-mGirTip1-v0.1 and T2T-mGirTip1-v1.0. Columns: (1 and 2) telomere presence on p- and q-arms. chr1_pat in v0.1 has two rows for each scaffold; (3 and 4) QV values for each assembly; (5) QV improvement; (6 and 7) number of gaps. Primary contigs are denoted with an asterisk (∗). See also Figure S11 and Table S1. (D) NG(X)% by version and haplotype. The total length of v1.0 for each haplotype was used as the genome size for the corresponding haplotype assembly. (E) Percentage of coverage abnormality categories from flagger by version and read type, using reads aligned to the diploid genome. (F) Stacked bar plot of BUSCO^51^ genes by version and haplotype. See also Figure S12. (G) Statistics of Illumina short-read alignment by version and haplotype (https://broadinstitute.github.io/picard/).

To validate the improvement of the assembly solely from gap filling through path correction, we examined the T2T-mGirTip1v0.3 assembly prior to polishing. The coverage profiles of ONT and HiFi reads aligned to T2T-mGirTip1v0.3 showed broadly consistent coverage, with expected low coverage confined to telomeric or satellite-enriched regions known to exhibit sequencing biases (Figure 5A). When we inspected the alignments before and after gap filling for the same region, the coverage and alignment patterns were improved. Notably, in T2T-mGirTip1v0.1, Flagger^4^ statistics and alignment patterns revealed that not only near the gap but also ∼200 kb of adjacent regions exhibited alignment difficulties (Figure 5B). After gap filling, read alignments improved substantially. Haplotype-specific alignments (MAPQ > 0) were restored at the boundary of node utig4-2295, and coverage profiles stabilized across both HiFi and ONT alignments. Importantly, no residual anomalies were detected by either Flagger or the coverage issue track,^14^ which reports abnormal coverage based on dinucleotide composition and error *k*-mers. The overall impact of all changes made with Verkko-Fillet from the initial (v0.1) to the final polished (v1.0). The assembly on the giraffe was assessed by comparing various assembly quality metrics (Figure 5C and Table S1). In v1.0, all chromosomes contained telomeres at both ends, with only a single remaining gap on chr14_mat, introduced during contig joining. The overall QV also improved substantially, increasing from an average of 61.5–73.6. The largest improvement was observed on chr7_pat, where the QV increased from 62.0 to 77.4 (Figure 5C). We observed a reduction in QV exclusively in chr14_pat, likely attributable to low-confidence path selection across rDNA arrays, as described above. Consistent with this, the majority of error *k*-mers were concentrated within the rDNA arrays rather than distributed across other genomic regions. (Figures 5C and S11). The haploid, linear representation of the genome to be used as a reference was constructed by selecting a primary haplotype for each chromosome based on having fewer gaps and higher QV, yielding a gap-free assembly hereafter referred to as T2T-mGirTip1v1.0-Pri.

Assembly contiguity improved, with N50 (the length of the shortest contig contributing to 50% of the total assembly length) increasing from 148 to 162 Mb for the maternal haplotype and from 150 to 162 Mb for the paternal haplotype (Figure 5D; Table S2). Flagger^4^ statistics showed incremental improvements from v0.1 to v1.0, with a lower proportion of erroneous (Err), collapsed (Col), and duplicated (Dup), and a higher proportion of haploid-specific regions (Hap) in both HiFi and ONT alignments (Figure 5E). Gene content assessed with the benchmarking sets of universal single-copy orthologs (BUSCO)^51^ results was in line with Flagger, such that v1.0-Pri contained the highest proportion of single-copy gene predictions (98.2%), along with fewer missing or incomplete genes (%) (Figures 5F and S12; Table S2). Finally, mapping of Illumina short-read data from the mGirTip1 to each assembly version further demonstrated v1.0 yields higher alignment rates, fewer improper pairs, reduced indel rates, and fewer chimeric alignments (Figure 5G). Overall, as graph refinement, tuning, and polishing progress, the assembly quality is substantially improved, making it more useful for downstream analyses by enhancing gene content and mapping quality.

### The T2T-mGirTip1v1.0 genome provides a more accurate and biologically valid representation of giraffe chromosomes

**Figure 6:**
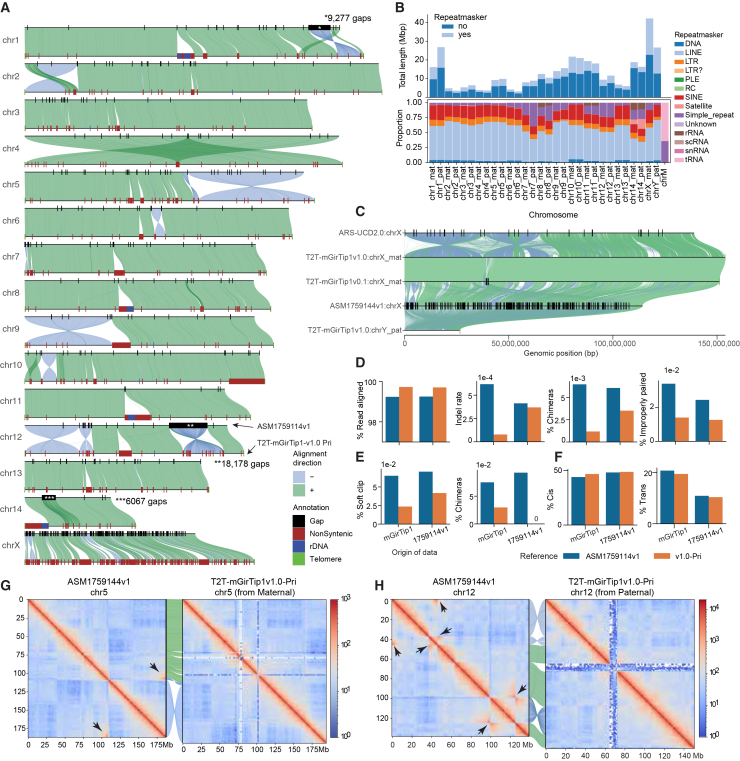
From draft to complete: The T2T-mGirTip1-v1.0 giraffe genome assembly (A) Syntenic plot showing structural differences between the T2T-mGirTip1-v1.0 primary assembly and the giraffe reference genome ASM1759114v1, generated using SVByEye.^53^ Top: ASM1759114v1 with gaps shown in black boxes. Bottom: T2T-mGirTip1-v1.0 primary contigs, with non-syntenic regions (brown), rDNA (blue), and telomeres (green). Regions with an excessive number of gaps are labeled with an asterisk and the number. See also Figures S13 and S14 and Table S2. (B) Total length of non-syntenic regions per chromosome (upper), with repeats in light blue color identified by RepeatMasker.^54^ Lower: the proportion of each repeat type within the non-syntenic regions. See also Table S3. (C) Syntenic comparison of the chrX of the cattle (ARS-UCD2.0), giraffes (v0.1, v1.0 and ASM1759114v1), and the chrY of T2T-mGirTip1-v1.0. Gaps (black) and telomeric regions (green) are marked on each contig. (D) Illumina short-read alignment statistics on T2T-mGirTip1-v1.0-pri and ASM1759114v1. The *x* axis indicates the dataset origin (mGirTip1 or ASM1759114v1). (E) ONT alignment statistics between assemblies. (F) Hi-C sequence data from each dataset aligned to the haploid genome, showing the percentage of *cis*- and *trans*-interactions. *cis*: paired within a chromosome. *Trans*: paired between chromosomes.^55^ (G and H) Hi-C contact maps using Hi-C data from mGirTip1 aligned to ASM1759114v1 and T2T-mGirTip1-v1.0 for chr5 (G) and chr12 (H). Interactions indicating the misassembled region in ASM1759114v1 are highlighted with black arrows. See also Figures S15–S18.

We next compared T2T-mGirTip1v1.0-Pri to the latest publicly available giraffe reference genome (GenBank: ASM1759114v1), derived from a male Rothschild’s giraffe (*Giraffa camelopardalis rothschildi*),^52^ assembled with ONT reads and scaffolded with Hi-C (Figure 6A). Compared with ASM1759144v1, the T2T-mGirTip1v1.0-Pri assembly demonstrates notable quality improvements (Table 1).

**Table 1:** Comparison of ASM1759114v1 and T2T-mGirTip1v1.0-Pri giraffe genome assemblies

STATISTICS	ASM1759114v1	T2T-mGirTip1v1.0-Pri	Difference
**Summary**			

Chromosomes	chr1-14, X	chr1-14, X, Y, M	new chrY and chrM
Number of contigs	2096	17	−2079
Total length (Gbp)	2.44	2.58	0.14
Minimum length (bp)	504	16430	15926
Average length (Mbp)	1.16	151.67	150.50
Maximum length (Mbp)	236.01	238.23	2.22
Q1 (bp)	1,732	150,165,456	150,163,724
Q2 (bp)	4,000	162,115,330	162,111,330
Q3 (bp)	9,026	192,562,917	192,553,892
N50 (Mbp)	158.2	166.6	8.4
N-gap bases (Mbp)	3.5	0.0	−3.5
Unplaced bases (Mbp)	50.0	0.0	−50.0

**BUSCO gene content**			

Single copy	96.4	98.2	1.8
Duplicated	2.6	1.3	−1.3
Fragmented	0.2	0.1	−0.1
Missing	0.8	0.4	−0.4

Q stands for quartile; Q1, Q2, and Q3 represent the 1st, 2nd, and 3rd quartiles, respectively. BUSCO gene contents are calculated using the Giraffa lineage. See also Table S2.

Whole-genome alignment between T2T-mGirTip1v1.0-Pri and ASM1759144v1 revealed 220 Mb of non-syntenic regions, of which 36.8% were composed of repeat sequences (Figures 6A and 6B; Table S3; Figures S13 and S14). The overall repeat composition of non-syntenic regions across chromosomes was largely similar, with LINEs and SINEs (long and short interspersed nuclear elements) comprising the majority of repetitive elements (Figure 6B). However, certain chromosomal regions displayed notable enrichment, including rRNA repeats on chromosomes 8, 11, and 14, and tRNA repeats on chr14 (Figures 6A and 6B).

Most notably, the sex chromosomes were substantially improved in the T2T-mGirTip1v1.0 assembly (Figure 6C). The previous reference genome, ASM1759144v1, lacks an assigned Y and contains 288 gaps scattered across the X, totaling 145 kb out of 114 Mb. This is likely due to incorrectly scaffolded sequences from both sex chromosomes into the X, which in turn contributed to the numerous gaps and fragmented syntenic regions observed between the X and Y.^56^^,^^57^ In contrast, T2T-mGirTip1v1.0 fully resolves both the X and Y separately, without any gaps, adding 42.21 Mb (27.4%) and 26.62 Mb (100%) of sequences, respectively. Alignment to the cattle chrX confirmed two previously reported inversions on the p-arm of the new giraffe chrX.^47^^,^^58^ One of the breakpoints matches the location of a gap in T2T-mGirTip1v0.1, suggesting that this region is highly repetitive.

To confirm T2T-mGirTip1v1.0 suits better as the giraffe reference, we compared alignments of Illumina, ONT and Hi-C data from both mGirTip1 and ASM1759144v1 (Figures 6D–6F). Alignment of Illumina data showed T2T-mGirTip1v1.0 had a higher proportion of properly aligned reads, along with lower portions of chimeric reads, indel rates, and improper pairs compared to ASM1759144v1 (Figure 6D). Similarly, ONT data alignment revealed fewer reads with soft clipping and chimeric signatures when mapped to T2T-mGirTip1v1.0 (Figure 6E). Hi-C alignment further demonstrated improved performance, with T2T-mGirTip1v1.0 showing a higher number of *cis*- interactions and fewer *trans*-interactions than ASM1759144v1 (Figure 6F).

Extensive synteny was observed between the T2T-mGirTip1v1.0 assembly and ASM1759144v1 across many chromosomes, yet several chromosomes exhibited notable orientation discrepancies in specific regions (Figures 6A and S14). In particular, chr1, 2, 5, 6, 9, 10, and 12 displayed large-scale structural differences, including apparent inversion and translocation errors (Figure 6A). We confirm that this did not originate from a giraffe individual- or species-specific structural variant by aligning Hi-C data from both giraffe samples (T2T-mGirTip and ASM1759144v1). Long-range interactions were consistent with the breakpoints identified in syntenic regions (Figures S15–S18). For example, the Hi-C contact maps of chr5 in ASM1759144v1 revealed a large inversion on the q-arm (Figures 6G and S18). Chr12 in ASM1759144v1 contained multiple inversions, whereas no off-diagonal interactions were observed in the T2T-mGirTip1v0.1 contact map (Figures 6H and S18). These results support the structural accuracy of T2T-mGirTip1v1.0 and suggest that T2T-mGirTip1v1.0 represents a more reliable reference for the giraffe species.

Gene annotation of the T2T-mGirTip1v1.0 assembly was performed with EviAnn.^59^ Illumina RNA sequencing (RNA-seq) and PacBio Kinnex sequencing data were used as transcriptomic evidence, and a set of 576,821 mammalian proteins served as protein homology evidence from UniProt-SwissProt database.^60^ We identified 20,848 protein-coding genes and 2,785 non-coding genes (Table S4). Additionally, 85,186 isoforms were predicted as protein coding, of which 83,131 were functionally annotated, and 61,891 showed strong homology to known proteins in the primary assembly. BUSCO^51^ analysis derived from this new annotation further confirmed that T2T-mGirTip1v1.0 is highly complete in terms of gene content, based on the mammalia database (Table S5).

Collectively, T2T-mGirTip1v1.0 represents the first high-quality T2T giraffe genome, correcting many large assembly errors present in previous references and adding new sequences that were previously difficult to assemble using the Verkko and Verkko-Fillet workflows. We also provide both gene and transcriptome annotations for the diploid assembly, offering a valuable resource for biological research.

### Verkko-Fillet improves a benchmark human genome assembly

We applied Verkko-Fillet to another genome assembly, the benchmark HG002 human genome^26^ to demonstrate its compatibility. We built the initial assembly using HiFi (65.9×), ONT-UL (507.7×, 134.5× over 100 kb), and Hi-C (51.8×) data with Verkko (v2.3) (see STAR Methods). Verkko-Fillet successfully filled gaps and recovered telomeres with post-consensus trimming (Table S6). Gap filling alone substantially improved the QV by 13.9 for chromosome 3 haplotype 1 by resolving two gaps, and the number of T2T-scaffolds increased from 38 to 45. When comparing the initial and post-Verkko-Fillet assemblies to the latest curated T2T-HG002v1.1 benchmark genome, auNGA (a metric measuring contig alignment and contiguity) increased from 113 to 117 Mb (Figure S19), and the number of assembly errors decreased by 1,959 (Table S6). These results demonstrate the compatibility and applicability of Verkko-Fillet.

The computational requirements of Verkko-Fillet are generally modest, as it operates primarily as a lightweight helper package, leveraging pandas dataframes and scikit-learn-based statistics for gap filling. Some steps depend on external tools (e.g., GraphAligner,^41^ Meryl,^61^ and Seqtk^62^) that are more resource-intensive (Table S7). In contrast, the core gap-filling procedures involve the storing and processing of graph and alignment information, which are computationally efficient.

## Discussion

Multiple genome assembly tools, such as Verkko^10^^,^^11^ and Hifiasm,^8^ employ graph-based representations, where nodes denote sequences, edges represent their connectivity, and paths define haplotype-resolved contigs. Despite advancements of these frameworks, manual curation of assembly graphs remains limited. As a result, unresolved gaps and structural errors frequently persist in the final consensus sequences, requiring correction prior to polishing to avoid mismapping of the reads.

Here, we present Verkko-Fillet, an interactive tool that integrates seamlessly into the Verkko genome assembly workflow to guide assembly graph curation. Verkko-Fillet provides tools for graph inspection, path curation, and error correction and regenerates the refined consensus, lifting a draft genome assembly to a finalized, structurally curated genome. As demonstrated on the giraffe and human genomes, our results show substantial improvements in assembly quality through graph correction and gap filling, even prior to polishing (Table S1). Consistently, alignment statistics and coverage uniformity across all sequencing platforms were markedly improved. Verkko-Fillet supports post-consensus procedures such as chromosome renaming, reorienting, rDNA patching, and polishing to produce a submission ready, complete T2T assembly.

The input data representation in Verkko-Fillet is the Verkko assembly graph, along with associated paths that represent assembled sequences. Users can edit these paths to correct assemblies, guided by supporting evidence such as haplotype information, coverage profiles, and alignments from Hi-C and ONT reads. While this approach could in principle be extended to other assemblers that employ graph-based representations, it is currently limited to the Verkko framework. Support for other assemblers could be a potential area of future development.

We demonstrate the complete workflow for generating the telomere-to-telomere giraffe genome (T2T-mGirTip1v1.0) using Verkko-Fillet, highlighting improvements in QV, gene content, and NG50, supported by detailed examples. T2T-mGirTip1v1.0 does not contain the arm-level assembly errors present in previous references and shows the expected synteny with cattle. In addition, we identified 220 Mbp of new sequences in non-syntenic regions, including proximal centromeric and rDNA sequences, as well as intact T2T contigs for chrX and chrY, and the complete chrM. Additionally, we provide a preliminary diploid gene annotation with 40,672 protein-coding genes. This resource will support studies in conservation and ruminant biology.

We also applied Verkko-Fillet to a draft assembly of the HG002 human genome and confirmed its ability to improve assembly quality by comparison to the T2T-HG002 genome benchmark. Additionally, Verkko-Fillet does not require substantial computational resources, making it a lightweight and practical helper tool for assembly graph curation. Overall, Verkko-Fillet is a compatible and efficient tool that can be applied to a variety of assemblies and helps streamline the genome refinement workflow.

### Limitations

Verkko-Fillet is designed as a helper tool and provides guidance to facilitate quality control and assist in correcting paths rather than to fully automate the entire process. Several steps, specifically gap filling, require manual inspection of the graph, which could be time-consuming. Graph curation depends heavily on the user’s expertise and the desired level of completeness. Therefore, assembly results may vary among user. Verkko-Fillet depends on sufficient ONT read length and a correct and complete assembly graph for resolving repeats, which limits its ability to resolve large, nearly identical repeat arrays, such as human rDNAs.

## Resource availability

### Lead contact

For additional information, resources, requests should be directed to the lead contact, Arang Rhie, PhD (arang.rhie@nih.gov).

### Materials availability

The giraffe samples are available upon request.

### Data and code availability

Verkko-Fillet is a Python package installable with pip along with the provided conda environment file, publicly available on GitHub (https://github.com/marbl/verkko-fillet) and Zenodo (doi: 10.5281/zenodo.19867931), along with a full documentation. Sequencing data and assemblies of the giraffe are available under the umbrella BioProject PRJNA1322564. RefSeq annotation of the primary assembly is available under GCF_054371585.1. Annotations produced from this study are available on GitHub (https://github.com/jjuhyunkim/giraffeT2T) and Zenodo (https://doi.org/10.5281/zenodo.19867517). The final curated HG002 diploid assembly is available at Zenodo (https://doi.org/10.5281/zenodo.19868173).

## Acknowledgments

We thank the Cincinnati Zoo and the Nashville Zoo for providing samples for the giraffe family. We thank the members of the Ruminant T2T Consortium (https://www.ruminant2t.org/) for conceptualizing and providing insightful comments during the development of this work. We also thank Steven Solar for sharing preliminary ideas for coordinate transformation between the assembly graph and the final consensus and Terence Murphy and his team for RefSeq annotation. J.K. was supported by a grant of the Korea Health Technology R&D Project through the 10.13039/501100003710Korea Health Industry Development Institute (KHIDI), funded by the Ministry of Health & Welfare, Republic of Korea (RS-2022-KH131838). J.K., A.M.P., S.K., and A.R. were supported by ZIA HG200398. J.J.S. was supported by the 10.13039/501100000268BBSRC BBS/E/D/10002070. L.W.-C. and D.R.C. were supported by Eberly College of Science and the Huck Institute of the Life Sciences, Penn State University. A.V.Z. was supported by the NSF IOS-2432298. This work utilized the computational resources of the NIH HPC Biowulf cluster (https://hpc.nih.gov) and the USDA ARS SCINet initiative. ChatGPT5.3 was used to rephrase text for fluency and was reviewed by the authors. The authors take full responsibility for the published article. This research was supported in part by the 10.13039/100030692Intramural Research Program of the 10.13039/100000002NIH. The contributions of the NIH authors are considered Works of the United States Government. The findings and conclusions presented in this paper are those of the authors and do not necessarily reflect the views of the NIH or the US Department of Health and Human Services. Any mention of trade names or commercial products is solely for the purpose of providing specific information and does not imply recommendation or endorsement by the USDA. The USDA is an equal opportunity provider and employer.

## Author contributions

J.K. designed Verkko-Fillet, performed data analyses, and drafted the manuscript. B.D.R. generated the initial T2T-mGirTip1v0.1 Verkko assembly. J.K., B.D.R., S.E.F., and J.J.S. optimized Verkko-Fillet. A.L. and H.S. collected the samples. D.R.C., L.W.-C., and K.L.K. generated sequencing data. A.V.Z. conducted gene annotation. S.K. supervised compatibility between Verkko-Fillet and Verkko. A.R. provided feedback on the polishing step and handled data submission. T.P.L.S., A.M.P., S.K., and A.R. supervised the project. J.K. and A.R edited the manuscript with the assistance of all authors. All authors read and approved the final manuscript.

## Declaration of interests

S.K. has received travel funding for speaking at events hosted by ONT.

## STAR★Methods

### Key resources table

**Table:** 

REAGENT or RESOURCE	SOURCE	IDENTIFIER
**Critical commercial assays**		

UHMW Blood extraction protocol	Circulomics	EXT-BLU-001
Tempus™ Blood RNA Tube	Thermo Fisher	4342792
Tempus™ Spin RNA Isolation Kit protocol	Thermo Fisher	4380204
TRIzol Reagent	Thermo Fisher	15596026
Qiagen RNAeasy protocol	Qiagen	74104
dsDNA BR Assay kit	Thermo Fisher	Q33266
SMRTbell-prep-kit-3.0	Pacbio	102-182-700
Revio SPRQ polymerase kit	Pacbio	103-520-100
IsoSeq express 2.0 kit	Pacbio	103-071-500
Binding kit 3.2 and cleanup beads	Pacbio	102-333-300
Kinnex full-length RNA kit	Pacbio	103-072-000
Blue Pippin High Pass 0.75% Agarose	Sage Science	BLF7510
Ligation Sequencing Kit LSK-114	ONT	LSK-114
AMPure XP Beads for DNA Cleanup, 60 mL	Beckman Coulter	A63881
Ultra-Long DNA Sequencing Kit V14 (ULK-114)	ONT	ULK-114
TruSeq DNA PCR-Free Kit	Illumina	20015962
Illumina Stranded mRNA libraries	Illumina	20040532
Dovetail Omni-C Protocol for Tissues v2.0	Dovetail Genomics	PN DG-REF

**Software and algorithms**		

Verkko2 (version 2.2.1)	Dmitry Antipov et al.^10^	https://github.com/marbl/verkko
Meryl (version 1.4.1)	Rhie, A et al.^61^	https://github.com/marbl/meryl
Merqury (version 1.3)	Rhie, A et al.^61^	https://github.com/marbl/merqury
minimap2 (version 2.30)	Heng Li.^63^	https://lh3.github.io/minimap2/
Bedtools (version 2.31.1)	Aaron R. Quinlan et al.^64^	https://bedtools.readthedocs.io/en/latest/
winnowmap (version 2.03)	Chirag Jain et al.^65^	https://github.com/marbl/Winnowmap
DeepVariant (version 1.6.1)	Ryan Poplin et al.^66^	https://github.com/google/deepvariant
NucFlag (version 0.3.3)	Mitchell R Vollger et al.^50^	https://github.com/logsdon-lab/NucFlag
BUSCO (version 6.0.0)	Felipe A. Simão et al.^51^	https://github.com/metashot/busco
Picard (version 3.3.0)		https://github.com/lh3/bwa
Flagger (version 1.1.0)	Wen-Wei Liao et al.^4^	https://github.com/mobinasri/flagger
Singularity (version 4.2.2)		https://github.com/sylabs/singularity
Paritools (version 1.1.0)	Open2C et al.^55^	https://pairtools.readthedocs.io/en/latest/
BWA-MEM (version 0.7.17)	Heng Li et al.^67^	https://github.com/lh3/bwa
Cooler (version 0.10.4)	Nezar Abdennur et al.^68^	https://cooler.readthedocs.io/en/latest/
Mashmap (version 3.1.1)	Chirag Jain et al.^69^	https://github.com/marbl/MashMap
RepeatMasker (version 4.2.1)		https://www.dfam.org/browse?clade_descendants=true
Dfam (version 3.9)		https://www.dfam.org/browse?clade_descendants=true
GraphAligner (version 1.0.19)	Mikko Rautiainen et al.^41^	https://github.com/maickrau/GraphAligner
GQC (version 0.1.0)	Hansen NF et al.^26^	https://gqc.readthedocs.io/en/latest/
Verkko-fillet	This paper	https://verkko-fillet.readthedocs.io/en/latest/index.html

**Deposited data**		

Sequencing data from mGirTip1, mGirTip2, mGirTip3 and mGirTip4	This paper	NCBI BioProject: PRJNA1322564
T2T-mGirTip1v1.2 assembly and RefSeq annotation	This paper	NCBI RefSeq: GCF_054371585.1
Giraffe genome annotations	This paper	https://doi.org/10.5281/zenodo.19867931
HG002 assembly	This paper	https://doi.org/10.5281/zenodo.19868173

### Experimental model and study participant details

#### Sample collection

Blood and tissue samples were collected from a Masai giraffe (*Giraffa tippelskirchi)* family at the Cincinnati Zoo and Botanical Garden, Cincinnati, OH USA and Nashville Zoo, Nashville, TN USA. For the paternal genome (Kimba, mGirTip3; DOB 2007), the skin samples were collected postmortem from his legs that were saved and frozen (−4°) and gDNA was isolated. He died in 2019 after an anesthetic procedure to assess chronic lameness issues. He was 12years old at the time of death. He was overall healthy other than his hoof issues causing the lameness. For the maternal sample (Tessa, mGirTip2; DOB 2006), tail hair root cells were collected when the animal was 15 years old and gDNA isolated. For the offspring (Fennesey, mGirTip1; DOB 2019), testes sample was collected when the animal was 2 years old during the castration procedure, and flash frozen in liquid nitrogen to preserve RNA integrity. Later, ∼12 cc of EDTA whole blood was collected from the left jugular vein with a 21 ga butterfly catheter and vacutainer setup when mGirTip1 turned 5 years old, and immediately frozen in liquid nitrogen to preserve chromosomal DNA integrity. Tessa and Fenn are both at the Cincinnati Zoo and are healthy. An older sibling of mGirTip1 (Nasha; DOB 2014), who is sharing the same parents as mGirTip1, was shipped to the Nashville Zoo in 2015. She gave birth to a calf in 2021, mGirTip4, who died from a neck injury along the cervical vertebrae shortly after birth. Postmortem tissues were collected from the cardiac muscle that were saved and frozen at −80F. Nasha died in the summer of 2025 due to a gastrointestinal torsion.

### Method details

#### Data generation

##### DNA and RNA extractions

High-molecular-weight DNA was isolated either from whole blood using the UHMW Blood extraction protocol (Circulomics) or from testes tissue utilizing an HMW Phenol:Chloroform protocol.

RNA was isolated from blood using the Tempus Blood RNA Tube and Tempus Spin RNA Isolation Kit protocol (ThermoFisher) or from tissues utilizing a TRIzol Reagent (Invitrogen) and Qiagen RNAeasy protocol (Qiagen).

##### Pacific Biosciences HiFi long-read sequencing

Pacific Bioscience (PacBio) HiFi libraries were generated for the mGirTip1. DNA was sheared using a Diagenode Megaruptor 3 to 20 kb mode size. At all steps, DNA quantity was checked on a Qubit Fluorometer with a dsDNA BR Assay kit (Thermo Fisher), and sizes were examined on a Fragment Analyzer (Agilent Technologies). SMRTbell libraries were then prepared for sequencing according to the protocol.

‘Procedure-checklist-Preparing-whole-genome-and-metagenome-libraries-using-SMRTbell-prep-kit-3.0’. The libraries were then size-selected on the Blue Pippin using the BLF-7510 cassette under the 0.75% DF Marker S1 high-pass 15–20 kb protocol (Sage Science) to remove fragments below 15 kb in size. The selected library fractions were bound with the Revio Polymerase Kit and sequenced on a Revio instrument (PacBio) with 30 h movie time. 228 Gb of HiFi data was generated.

PacBio Iso-Seq libraries enable direct sequencing and analysis of full-length transcript isoforms. A PacBio Iso-Seq library was created using the ‘Preparing Iso-Seq v2 libraries using SMRTbell prep kit 3.0’ from the testes sample from the mGirTip1. The library was bound with the Sequel II Polymerase Kit and run on one Sequel II SMRT Cell in a 24-h movie.

The PacBio Kinnex library preparation kits are based on the MAS-Seq method, which concatenates smaller amplicons into larger fragment libraries (Pacific Biosciences). Full-length RNA Kinnex libraries were made from RNA extracted from both the blood sample and the testes following the protocol ‘Preparing-Kinnex-libraries-using-the-Kinnex-full-length-RNA-kit.’ Kinnex libraries are pooled and bound with the Revio Polymerase Kit and run on a total of 7 Revio SMRT cells in 24-h movies.

##### Oxford Nanopore long-read sequencing

For the mGirTip1, a template for long read sequencing on the PromethION instrument was prepared using both the Ligation Sequencing Kit LSK-114 and the Ultra-Long DNA Sequencing Kit ULK-114 (Oxford Nanopore Technologies, Oxford, UK). For the LSK-114 kit, modifications to the DNA handling and cleanup procedure were utilized (https://community.nanoporetech.com/posts/rocky-mountain-adventures). 10 μg of DNA was extracted from blood and testes, cleaned and concentrated by addition of 1 volume of AMPureXP bead solution. After collecting the beads by magnet, the beads were washed 2 times with 0.5 mL 80% ethanol and dried for 1 min. The beads were suspended in a 51 μL elution buffer (EB) and DNA eluted at 37°C for 15 min. The sheared, eluted DNA was then processed by ligation to the ligation adapter (LA) as recommended by the manufacturer’s instructions, apart from an increase in the room temperature incubation of the ligation reaction to 60 min. Libraries were sequenced in 21 flow cells on a PromethION instrument using R10.4.1 flow cells. Two Ultra-Long DNA Sequencing Kit V14 (ULK-114) libraries were prepared, one from blood and one from testes tissue, following manufacturer protocols (Oxford Nanopore Technologies, Oxford, UK) and run on PromethION v10.4.1 flow cells.

##### Illumina short-read sequencing

DNA from the mGirTip1 was then processed into a short-read library using the TruSeq DNA PCR-Free Kit as recommended by the manufacturer (Illumina Inc., San Diego, CA) and sequenced on an Illumina NextSeq2000 platform using 2 × 151 base paired end reads. 147 Gb of total data was obtained.

An Illumina Stranded mRNA library was generated from testes tissues for the mGirTip1. Illumina Stranded mRNA libraries utilize a rapid, cost-effective workflow for accurate, unbiased detection of the protein-coding transcriptome with precise measurement of strand information (Illumina, San Diego, CA). The Illumina Stranded mRNA library was sequenced on the Illumina NextSeq2000 instrument (USDA MARC) with 2 × 101 base paired end reads.

To gather information about genome spatial organization for use in scaffolding, we generated a Dovetail Omni-C Hi-C library by cross-linking approximately 10 mg of testes tissue of mGirTip1. The Dovetail Omni-C Hi-C library was following the Dovetail Omni-C Protocol for Tissues v2.0 (Dovetail Genomics, Scotts Valley, CA). Omni-C libraries deliver uniform, shotgun-like sequence coverage that is utilized for enabling genotyping and haplotype phasing, alongside long-range information characteristic of Hi-C data. The library was sequenced on an Illumina NextSeq2000 sequencer with paired end 2 × 151 reads.

Whole-genome sequences of mGirTip3 and mGirTip2, the parents of mGirTip1, were generated using short-read Illumina sequencing. DNA for mGirTip3 came from postmortem skin tissue and for mGirTip2 from tail hair root cells collected at the Cincinnati Zoo and Botanical Garden. Libraries were prepared at the Pennsylvania State University Huck Institute’s Genomic Core and sequenced at the Penn State Hershey Genomics facility on a NovaSeq using 150 bp paired-end reads. Demultiplexing was performed with Bcl2fastq, which masked short adapter-overlapping bases. Read quality was assessed with FastQC v0.11.8, showing high base quality across reads, and Fastp v0.20.0 confirmed that adapter trimming was unnecessary.

#### T2T-mGirTip1v0.1 assembly

We assembled the giraffe genome with Verkko in trio mode using a combination of 90× PacBio HiFi and 45× Oxford Nanopore Technologies (ONT) duplex data (length >20 kb, Q > 20) passed to Verkko as long-accurate reads (--hifi), 370× ONT simplex data (45× reads greater than 100 kb) passed to Verkko as ultra-long reads (--nano), and Illumina data (38× maternal, 72× paternal) from the parents for phasing. The assembly was generated with Verkko v2.2 using the parameter --unitig-abundance 4 due to the high depth of long-accurate reads used. Verkko was then rerun using the trio output and 200 million Hi-C read-pairs to assist in scaffolding across unresolved tangles in the graph.

#### Verkko-Fillet functions

##### Verkko object

The Verkko object is a specialized Python class designed to consolidate extensive information into a single entity. This object can be easily saved and loaded from disk while maintaining a trackable history. It contains 11 key attributes, most of which are Pandas DataFrames,^70^ making them easy to parse and use independently.

• verkkoDir: Directory containing the verkko results.
• verkko_fillet_dir: Output directory for verkko-fillet results.
• paths: Path file extracted from assembly.paths.tsv.
• version: Genome version.
• species: Genome species.
• stats: Assembly statistics (e.g., contig and chromosome information, completeness).
• gaps: Gap information extracted from the.path file.
• gaf: Graph alignment results (GAF format).
• qv: Quality value (QV) calculation results using Meryl and Meruqury.
• history: Log of all modifications made during the gap-filling process.
• scfmap: Assembly scaffold mapping file (assembly.scfmap).

To generate the Verkko Fillet object from the Verkko result directory, the directory itself is the only required input.

obj = vf.pp.read_Verkko(<verkkoDir>)

##### QV score

To estimate the base-level accuracy of the assembly, we calculated QV scores using a *k*-mer–based approach implemented with Meryl (v1.4.1). First, 31-mers were generated independently from Illumina and HiFi read sets. k-mers present more than once were extracted from both datasets, and a hybrid *k*-mer database was then constructed by combining the filtered Illumina and HiFi *k*-mers. This hybrid k-mer set was compared against the *k*-mers from assembly to identify *k*-mers absent from the read data, which were considered potential sequencing or assembly errors. QV scores were computed using Merqury (v1.3),^61^ which can also be performed via the Verkko-Fillet function.

vf.tl.calQV(obj)

##### Contig stats

To retrieve T2T statistics, including the number of scaffolds, contigs, telomeres, and gaps, the final assembly.fasta file from the Verkko directory is used.

vf.tl.getT2T(obj)

This function uses the getT2T.sh script from the marbl training github (https://github.com/marbl/training/blob/main/part2-assemble/docker/marbl_utils/asm_evaluation/getT2T.sh). Specifically, it employs seqtk gap to identify gaps and seqtk telo to detect telomeres at both ends of contigs, with CCCTAA as the telomeric sequence. In some cases, seqtk telo fails to detect telomeres that are not sufficiently close to contig ends. To improve detection, the function first runs seqtk telo -d 50000. It then runs seqtk telo -d 50000 again after trimming 2,000 bp from both ends using trimfq -b 2000 -e 2000. Finally, it runs seqtk telo -d 5000 after an additional 4,000 bp trim using trimfq -b 4000 -e 4000. The detected telomeric regions are merged using bedtools, and the output files are saved in the chromosome_assignment folder within the main Verkko-Fillet directory. These files identify T2T contigs and provide BED annotations for telomere and gap regions.

##### N50 and L50

The N50, which measures the contiguity of an assembly, is calculated using the contig statistics from obj.stats. To determine the N50, all contigs (or scaffolds) in the assembly are arranged in descending order by length. The N50 value is the length of the shortest contig for which the sum of all contigs of equal or greater length covers at least 50% of the total assembly length.

vf.pl.n50Plot(obj)

The function reports the N50 and L50 value and generates a bar plot representing sequence lengths. The N50 value is displayed and saved along with the plot.

##### Chromosome assignment

If a reference genome for the species exists, chromosome assignment for the assembly can be performed. This is achieved by aligning the assembly to the reference using Mashmap.^69^

vf.tl.chrAssign(obj = obj, ref = ref)

This function runs Mashmap using the command: mashmap -r <reference> -q <assembly> --pi 95 -s 10000 -t 8. The results are then filtered to retain segments with a block length greater than 1 Mbp and an identity of 99% for chromosome assignment.

vf.pp.readChr(obj)

The Mashmap alignment results can be read into obj.stats using the readChr function. The obj.stats table includes chromosome assignments, T2T contig statistics, and completeness data from the getT2T function.

##### Graph alignment

To identify ONT reads that support path traversal across gaps for gap filling, we used GraphAligner^41^ (v1.0.19) to align ONT reads to the assembly graph.

vf.tl.graphIdx(obj)

The graph is indexed before running GraphAligner with --diploid-heuristic 21 31 and --diploid-heuristic-cache, which are heuristic parameters designed to better handle alignments in diploid genomes. Additional parameters related to seeding, extension, and indexing are also used to make this consistent with the Verkko assembler: --bandwidth 15, --seeds-mxm-length 30, --mem-index-no-wavelet-tree, and --seeds-mem-count 10000.

vf.tl.graphAlign(obj, ontReadList = "ont.list")

Using the index generated above, ONT reads are aligned to the graph using GraphAligner. In this step, the diploid-related parameters --diploid-heuristic 21 31 are used again. Additionally, the following parameters are applied: --multimap-score-fraction 0.99, --precise-clipping 0.85, --min-alignment-score 5000, --hpc-collapse-reads, --clip-ambiguous-ends 100, --overlap-incompatible-cutoff 0.15, --max-trace-count 5, and --mem-index-no-wavelet-tree. This process generates a GAF (Graph Alignment Format) file for each ONT read with its corresponding paths.

vf.pp.readGaf(obj)

This function reads and parses the GAF file, storing it in the obj.gaf attribute for use in downstream analysis.

##### Align HPC node sequences to the assembly for IGV compatibility

To locate each node within the assembly, which can be useful during gap filling, we provide the script _node_to_assembly.sh. This script requires a path file, the HPC node sequences, the assembly, and the name of the target contig.

First, the list of nodes is extracted from the target contig’s path, and the corresponding HPC sequences are retrieved from the HPC node sequence set. Next, the sequence of the target contig is extracted from the assembly, and an index is generated with minimap2 (v2.30)^63^ using the -H option, which enables homopolymer-compressed k-mers. The HPC node sequences are then aligned to this index with minimap2, and the resulting BAM file is converted to BED format using bedtools (v2.31.1)^64^ bamtobed.

##### Gap filling

The path file, assembly.paths.tsv, generated by the Verkko assembler, contains three columns: contig name, path, and assignment. The gap information can be retrieved from the path column in this file. When the Verkko-Fillet object is created using the read_Verkko() function, the path file is stored in obj.path.

vf.pp.findGaps(obj)

The gap information includes flanking nodes and haplotype details, which helps prevent confusion between different gaps on the same chromosome and gaps with the same flanking nodes but on different haplotypes. This function extracts that information and stores it in obj.gaps.

vf.pp.searchNodes(obj,[node list])

To count the number of ONT reads that support walks spanning the nodes flanking a gap, this function creates a new attribute, obj.paths_freq, by parsing obj.gaf. By default, it filters the ONT reads to retain only the alignment with highest MAPQ per read, preventing duplicate counting of the same read. The function then reports the number of supporting reads for each walk (path), considering both directions.

vf.pp.highlight_nodes(obj, node = <node>)

In some cases, one of the nodes in a bubble may already be used in the other haplotype, and the coverage for both nodes may be sufficient to consider them as part of a haplotype. In such cases, we can assign the ambiguous node to the other haplotype, even if there is no ONT support showing a direct path through the ambiguous node with flanking haplotype-specific nodes.

vf.pp.calNodeDepth(obj)

To estimate the number of loops in repeat regions and exclude nodes from complex regions, it is useful to calculate node coverage. This can be done by analyzing obj.gaf, where the number of aligned reads per node is counted. The raw read counts are then normalized by the node length to obtain coverage values. This coverage information is merged into obj.node, allowing both haplotype assignment and coverage metrics to be assessed together for better-informed filtering.

vf.pp.searchSplit(obj,node_list_input)

If an edge between nodes is missing in the middle of a contig, it is possible to identify supporting ONT reads that align to both sides of the contig. To do this, the function filters for ONT reads that align with more than 5,000 bp at the ends of each contig (within 5,000 bp from the boundary) and have a mapping quality (MAPQ) greater than 0.

Verkko requires at least three supporting reads by default to create an edge. Therefore, if only two reads meet these criteria, Verkko may fail to generate the corresponding edge. Due to the complexity of setting multiple thresholds/accuracy values, it is not currently exposed to the user. The same code is used in verkko-fillet with a custom threshold to resolve the gap as requested by the user. This function helps recover such cases by identifying the supporting ONT reads, which are then incorporated into the final path file to improve connectivity.

vf.pp.fillGaps(obj=obj, gapId=<gapid>, final_path=<new path>).

This function verifies the nodes and orientations of boundary elements to prevent incorrect gap filling. If discrepancies in node identity or orientation are detected at the boundaries, the function issues a warning.

Check the status and history of gap filling using vf.pp.checkGapFilling(obj). There is also a function to delete a specific gap, vf.pp.deleteGap(obj, gapId), to prevent incorrect fixes.

##### Detect internal telomere and trimming

Sometimes, artificial sequences extend beyond the true telomere, causing a contig to be incorrectly labeled as non-T2T. This issue can be resolved by trimming these artifacts, provided they are confirmed to be non-biological.

vf.tl.detect_internal_telomere(obj)

This function runs the script originally used in the VGP assembly project (https://github.com/VGP/vgp-assembly).^6^ Briefly, the script takes an assembly FASTA file as input and applies a distance threshold to define terminal and internal regions at both ends of each contig.

result_merged, tel = vf.pp.find_intra_telo(obj, file=file, loc_from_end=15000)

From the BED file generated by vf.tl.detect_internal_telomere(obj), adjacent telomeric regions are merged. For each merged region, the highest proportion of telomeric sequence is retained. By default, only merged regions with a telomeric proportion greater than 0.5 are kept. Additional filters are applied: the total length of the merged region must be greater than 0 bp, and the region must start at least 15,000 bp from the contig boundary. This function returns two DataFrame, <result_merged> with internal and non-internal telomeres for both ends for each contig with other information such as the length of contig, or chromosome assignment result. <result_merged> is going to be used for plot heatmap of proportion of the telomere for each contig for both sides of contig using vf.pl.percTel() function.

vf.pp.find_reads_intra_telo(tel, <line number>)

Another DataFrame, tel, is generated by vf.pp.find_intra_telo(obj) and is used to extract reads covering the region preceding the internal telomere. This helps validate the structure and assess whether the region is a true biological signal or an assembly artifact. In some cases, reads in this region may originate from only one platform—such as ONT—even when the assembly was built using both HiFi and ONT data. The function locates the relevant contig segment and extracts the corresponding reads based on their relative position in HPC space. To visualize the region, the vf.pl.readOnNode(tel, <line number>, df) function can be used.

vf.tl.runTrimming(obj, <dict>)

If a dictionary in BED file format is provided, with keys for contig, from, and to, this function trims the assembly (by default, assembly.fasta) to the specified region and saves the trimmed version with the suffix "_trimmed.fasta".

##### Finding broken contigs and connecting them

Not only gaps but also broken contigs need to be correctly connected by identifying their counterpart contigs. There are two scenarios that require contig connection: first, when a contig is broken into two pieces, but both fragments are long enough to be assigned a haplotype and chromosome; second, when one of the fragments is too short to be assigned a chromosome, necessitating its connection with the longer fragment.

Scenario 1: Both contigs are assignable to the same haplotype and chromosome

vf.pp.detectBrokenContigs(obj)

All T2T contig statistics, including haplotype and chromosome assignments for the main contigs, are stored in obj.stats. The function uses this information to identify pairs of contigs that belong to the same haplotype and chromosome. It then determines which chromosomes should be connected.

Scenario 2: One of the contigs is not assignable to a chromosome.

In this scenario, the sequences of nodes are aligned pairwise to identify the counterpart nodes on the other haplotype, allowing us to determine which contigs should be connected.

vf.tl.gfaToFasta()

We extracted the HPC (homopolymer-compressed) sequences of each node from the GFA^71^ file into a FASTA format. By default, the file assembly.homopolymer-compressed.gfa is used, and the resulting FASTA file is saved with the same prefix, but with a.fasta extension.

vf.tl.mapBetweenNodes()

The FASTA file containing node sequences is aligned pairwise using Mashmap,^69^ with self-alignments skipped by default.

vf.pl.nodeMashmapBlockSize(node = <node>)

The Mashmap output is parsed and used to sort all nodes aligned to a specific <node> (used as the query), based on the total aligned block size. The block sizes are grouped and summed by reference nodes to rank the best-matching counterparts.

##### Connect the two contigs to each other

vf.pp.connectContigs(obj, contig = <source>, contig_to = <target>, gap = <gapId>, at = <left of right>, flip = <False>)

After identifying the two contigs that should be connected, either contig can serve as the source or the target. At this stage, the orientation and order of the contigs are critical and must be carefully verified—preferably by inspecting the graph in Bandage^43^—before proceeding with the connection. This function does not modify obj.paths directly. Instead, it adds a new gap record to obj.gaps, containing the source and target contig names, their orientations, and connection order. These entries can later be processed by the vf.pp.updateConnect(obj) and vf.pp.writeFixedPaths(obj) functions, as described below.

##### Clean up the paths

After gap filling with the Verkko-Fillet module, the original paths must be updated to incorporate the newly resolved sequences. This process involves not only replacing gaps with the filled segments but also updating connections between nodes to reflect the improved contiguity.

To avoid misassignment of reads to irrelevant or unused nodes during the Verkko consensus step, it is recommended to filter out certain paths. Specifically, short paths composed only of disconnected nodes, nodes used exclusively in gap filling, or nodes that were never part of the original assembly should be excluded.

obj = vf.pp.keepContig(obj, contig_lst, path_lst)

By default, this function utilizes the list of contigs stored in obj.stats. However, if key contigs are missing—such as mitochondrial sequences or other small scaffolds—they should be added manually at this stage. The obj.paths DataFrame is then updated with a new column, ‘rm', which logs filtering decisions. Contigs designated for retention are labeled “keep_contig".

obj = vf.pp.checkDisconnectNode(obj, min_hpc_len=100_000)

When two contigs are connected using the vf.pp.connectContigs() function, the changes are recorded in obj.gaps, but not yet reflected in obj.paths. To finalize these connections, this function marks the source and target contigs within obj.paths. This annotation allows vf.pp.writeFixedPaths(obj) to correctly merge them by filtering out the target contig and connecting it with the source, preparing the structure for consensus generation.

obj, cludlst = vf.pp.keepNodesInUnresolvedGaps(obj)

Some nodes may not have been utilized in the final assembly but remain connected to paths without gaps. This typically occurs when the assembly graph contains many nodes, but tools like Rukki select only a subset for the final paths. These unused nodes can still attract reads, potentially diverting them from the correct locations.

To address this, a directed graph is constructed from obj.edges using NetworkX.^72^ The graph is traversed to identify all nodes upstream and downstream of each gap using nx.ancestors() and nx.descendants(). For gaps between newly connected contigs, edges linking the source and target are added. Nodes found within unresolved gaps are flagged with “keep_Nodes_in_unresolved_gaps" in the final path annotation.

path = vf.pp.writeFixedPaths(obj)

Once all filtering steps are complete, the original paths are replaced with the refined, gap-filled versions. Connections between source and target contigs are finalized, and redundant or trivial paths are removed. The finalized path file and corresponding GAF file are then written to disk, ready for the next step in the Verkko consensus workflow.

#### Run Verkko consensus using the final path with filling the gaps

Verkko automatically determines which steps to run by checking the presence and timestamps of relevant files. Therefore, it is important to ensure that the working directory is properly set up with all necessary files before running the consensus step. Additionally, if new edges are added, several files must be updated to include the corresponding supporting ONT reads and walks to ensure accurate consensus generation.

vf.pp.mkCNSdir(obj, <new_folder_path>, missingEdge=<True or False>)

This function generates a new folder, enabling Verkko to resume from the consensus step while incorporating any new nodes and edges introduced by the user during this stage.

Verkko --slurm -d <new_folder_path> [ --hifi <hifi reads> --nano <ont reads> ] --screen rDNA <rDNA.fasta> --screen mitochondria <mitochondrial.fasta> --paths <final path file> --assembly <original verkko directory> [--snakeopts "--dry-run"]

Since version 2.3, Verkko automatically inherits the read sets from the existing assembly, so this command can be simplified by removing --hifi and --nano.

To generate cleaner results and remove redundant sequences from rDNA, we used rDNA sequences and giraffe mitochondrial sequences (MT605038.1.fasta) to screen mitochondrial nodes. Before running Verkko, it is advisable to use the --snakeopts "--dry-run" parameter with the Verkko consensus command. This will allow you to verify that Verkko is only submitting jobs for buildPackages, cnspath, combineConsensus, and layoutContigs.

#### Tuning and polishing

##### Naming the unplaced nodes

The unplaced sequences were assigned chromosome information by leveraging the edges in the graph. Using the edge and node data from the GFA,^71^ we clustered connected nodes together and assigned chromosome labels based on nodes that could be reliably mapped to a chromosome. Prior to this step, we excluded nodes that were used multiple times across different chromosomes to prevent ambiguity.

duplicate_nodes, node_database = vf.pp.find_multi_used_node(obj)

This function lists all nodes from each path that has been assigned a chromosome in obj.stats and groups them by chromosome to create a clean list of nodes for each chromosome. It then identifies nodes that are used across two different chromosomes. For example, if node utig4-0 is used in both path_number_1 assigned to chr1 and path_number_2 assigned to chr2, the function will return utig4-0 as a “multiple-used" node to exclude it from naming with vf.pp.naming_contigs(). For the sex chromosomes, chrY is treated as chrX to prevent mislabeling of nodes in the PAR region as multiple-used. The function returns duplicate_nodes and node_database, which is a table containing chromosome information and the list of nodes from each path.

chrMap = vf.pp.naming_contigs(obj, node_database, duplicate_nodes)

Using the outputs from the previous function, this function reads the GFA^71^ to obtain edge information and constructs an undirected network using the Python NetworkX package.^72^ It then clusters the nodes based on their connections while excluding nodes that are marked as “multiple-used." This process results in a clean cluster of fully connected nodes. Chromosome information is assigned to all nodes within each cluster if the chromosome information from any node in the cluster is unique. This allows us to determine the chromosome assignment for unplaced nodes. The output, chrMap, is a table containing the contig names from the assembly, along with their assigned chromosome and haplotype names.

vf.tl.renameContig(obj, chrMap)

The renaming of all contigs in the assembly is performed using this function. The output FASTA file is saved with the suffix _sorted.fasta appended to the input assembly file name.

##### Flipping and sorting assembly

The assembly may have the reverse orientation compared to the existing reference or the reference for orthologous species. Before finalizing the assembly, it is recommended to align its orientation with that of the widely used reference and ensure it is properly sorted.

vf.tl.flipContig(<list of contig to be flipped>)

The assembly.fasta file in the Verkko-Fillet directory is used, and the output is saved with the suffix _flipped.fasta in the same directory.

vf.tl.sortContig(sort_by = “hap”)

There are two options for the sort_by parameter: one based on haplotype (sort_by = “hap") and the other based on chromosome number (sort_by = “chr"). The default option is hap.

##### Polishing

Assembly errors were corrected using the variant calling method described by McCartney et al.^14^ and Yoo et al.^49^ To do this, we constructed a haploid reference that included haplotype contigs along with chrX, chrY, and chrM, resulting in T2T-mGirTip1v0.2-Mat and T2T-mGirTip1v0.2-Pat. Illumina and HiFi reads were aligned to both haploid and diploid assemblies, whereas ONT reads were aligned only to the diploid assembly.

Illumina reads were aligned with bwa^67^ and then deduplicated using samtools^73^ markdup. ONT and HiFi reads were aligned with winnowmap (v2.03),^65^ using the -ax map-ont and map-pb parameters, respectively. Primary alignments were retained by filtering with samtools view -F0x104. Finally, BAM files from HiFi and Illumina were merged for each reference with samtools merge, hereafter referred to as the hybrid.

Hybrid alignments on haploid assemblies were processed with DeepVariant (v1.6.1)^66^ using the HYBRID_PACBIO_ILLUMINA mode, with a MAPQ threshold of 1 for BAM files aligned to haploid assemblies and 0 for those aligned to the diploid assembly. Variant calling from ONT alignments was performed with ONT_R104, applying a MAPQ threshold of 0.

VCF files from hybrid alignments on T2T-mGirTip1v0.2-Mat, Pat, and diploid (Dip) assemblies, as well as ONT alignments on T2T-mGirTip1v0.2-Dip, were passed to the snv_candidates.sh script.^49^ The script was run with the hybrid k-mer database and an estimated haploid peak of 68, derived from the hybrid k-mer multiplicity distribution, to generate the final VCF file. Polishing variants were then applied to produce version T2T-mGirTip1-v0.2.4, excluding variants on chrM, using bcftools^74^ consensus -H.

##### rDNA patching

Even after generating the consensus with corrected variants, a high density of error k-mers remained within one rDNA unit on the chr14 paternal haplotype. To address this, we removed that unit and joined the two flanking units at the start site of the 45S alignment between consecutive units. This was done by creating three separate FASTA files: each was broken at the start site of the 45S alignment of the high-density error k-mers and the start site of the following unit, after which the first and last files were connected. The resulting assembly is T2T-mGirTip1v1.0-Dip.

##### Mitochondrial DNA circularization

Three mitochondrial contigs were identified in T2T-mGirTip1v0.2-Dip using the screen-assembly.pl script from the Verkko library. The longest contig was then circularized with the circularize_ctgs.py script, also from the Verkko library. This final circularized mitochondrial contig was included in the submitted genome.

### Quantification and statistical analysis

#### Assembly assessment

##### NucFlag

The nucleotide frequency plots and genome misassembly region file was generated using NucFlag (v0.3.3)^50^ with HiFi alignments on the T2T-mGirTip1-v0.3 genome, applying default parameters (Figure 5A).

##### BUSCO

For each assembly, we assessed completeness using BUSCO (v6.0.0)^51^ with the cetartiodactyla_odb10 lineage dataset. To compare BUSCO scores between T2T-mGirTip1-v0.1 and v1.0, chrX, chrY, and chrM were also included in each haplotype assembly.

##### Benchmark using read alignment

Read alignments on T2T-mGirTip1-v0.1 were performed using the same read types and corresponding parameters described in [Polishing in Method]. Alignment quality statistics were obtained with Picard (v3.3.0, http://broadinstitute.github.io/picard) CollectAlignmentSummaryMetrics for each BAM file using default parameters.

We also compared alignment statistics between ASM1759144v1, T2T-mGirTip1-v1.0, and the primary assembly. Illumina and ONT data were downloaded from the NCBI database (BioProject: PRJNA627604)^52^ and aligned to the corresponding references using the same parameters as for the T2T-mGirTip1 alignment. Quality was then assessed with Picard CollectAlignmentSummaryMetrics.

##### Flagger

We used the read-mapping-based tool Flagger (v1.1.0)^4^ to detect mis-assemblies in dual or diploid genome assemblies. The Singularity image was downloaded from the Flagger GitHub repository, and the tool was executed with Singularity (v4.2.2) using ONT and HiFi alignments on T2T-mGirTip1-v0.1 and T2T-mGirTip1-v1.0. The workflow consisted of two steps: (1) converting BAM files to COV format, and (2) detecting abnormal coverage.

For the first step, we used the following command, where platform (pf) represents either HiFi or ONT:

singularity exec flagger.sif ∖

bam2cov --bam ${pf}_to_dip.pri.bam ∖

--output ${pf}_coverage_file.cov.gz ∖

--runBiasDetection ∖

--annotationJson annotations_path.json ∖

--baselineAnnotation genome.size

For the second step, we used the following command, with parameters adjusted according to pf. For ONT, we specified

alpha = alpha_optimum_trunc_exp_gaussian_w_8000_n_50.ONT_R1041_Dorado_DEC_2024.v1.1.0.tsv

with winlen = 8000. For HiFi, we specified

alpha = alpha_optimum_trunc_exp_gaussian_w_16000_n_50.HiFi_DC_1.2_DEC_2024.v1.1.0.tsv

with winlen = 16000. Alpha TSV files were downloaded from the HMM-Flagger GitHub repository (https://github.com/mobinasri/flagger).

singularity exec flagger.sif ∖

hmm_flagger ∖

--input ${pf}_coverage_file.cov.gz ∖

--outputDir ${pf}_hmm_flagger_outputs ∖

--alphaTsv ${alpha} ∖

--labelNames Err,Dup,Hap,Col ∖

--windowLen ${winlen}

##### Hi-C alignment

Hi-C data from ASM1759114v1 were downloaded from the NCBI database (BioProject: PRJNA627604).^52^ To assess mapping quality, Hi-C reads from ASM1759114v1 and mGirTip1 were aligned to both T2T-mGirTip1v1.0-Pri and ASM1759144v1 using bwa mem -5SP (v0.7.17).^67^ The resulting SAM files were converted into pairs format using pairtools (v1.1.0)^55^ with sorting and deduplication. A mapping quality threshold of 1 was applied for the haploid assembly (ASM1759114v1 and T2T-mGirTip1v1.0-Pri).

The commands used for this analysis are provided below:

bwa mem -t $thread -SP $ref $hic1 $hic2 | ∖

pairtools parse -c $ref.size --min-mapq $mapq |∖

pairtools sort |∖

pairtools dedup ∖

--output $asm.nodups.pairs.gz ∖

--output-dups $asm.dups.pairs.gz ∖

--output-unmapped $asm.unmapped.pairs.gz ∖

--output-stats $asm.dedup.stats

Contact matrices were generated from deduplicated pairs files using cooler (v0.10.4).^68^ For each assembly ($asm), we loaded pairs into a single-resolution.cool at bin size $bin using cooler cload pairs, then produced a multiresolution.mcool with cooler zoomify at 100 kb, 500 kb, 1 Mb, and 2 Mb. Zoomified matrices were balanced during processing.

cooler cload pairs ∖

-c1 2 -p1 3 -c2 4 -p2 5 ∖

--assembly $asm ∖

$ref.size:$bin ∖

$asm.nodups.pairs.gz ∖

$asm.$bin.cool

cooler zoomify ∖

--nproc $thread ∖

--out $asm.$bin.mcool ∖

--resolutions 100000,500000,1000000,2000000 ∖

--balance ∖

$asm.$bin.cool

#### Genome annotation

##### Non-syntenic regions

We mapped T2T-mGirTip1-v1.0 (query) to ASM1759144v1 (reference) using Mashmap (v3.1.1)^69^ with a segment length of 50 kb and a minimum percent identity of 99%. Mashmap output was converted to BED, restricted to chromosome-consistent matches, and subtracted from the T2T-mGirTip1-v1.0 whole genome length BED to identify non-syntenic regions.

mashmap -r $ref ∖

-q $query ∖

--pi 99 ∖

-s 50000 ∖

-o ASM1759144v1.T2T-mGirTip1-v0.1.syntenic.mashamp

awk '{print $1,$3,$4,$6,0,$5}' OFS='∖t' ∖

ASM1759144v1.T2T-mGirTip1-v0.1.syntenic.mashamp ∖

> ASM1759144v1.T2T-mGirTip1-v0.1.syntenic.mashamp.bed

awk '{print $1,$0}' OFS='∖t' ∖

ASM1759144v1.T2T-mGirTip1-v0.1.syntenic.mashamp.bed |∖

sed -e 's/_.at//' |∖

awk '$1==$5 {print $2,$4,$5}' OFS='∖t' ∖

> ASM1759144v1.T2T-mGirTip1-v0.1.syntenic.mashamp.chr_match.bed

bedtools subtract -a assembly_dim.fasta.bed -b ∖

ASM1759144v1.T2T-mGirTip1-v0.1.syntenic.mashamp.chr_match.bed ∖

> ASM1759144v1.T2T-mGirTip1-v0.1.non.syntenic.mashamp.chr_match.bed

##### Repeat annotation

The repeats in T2T-mGirTip1v1.0-Dip were annotated using RepeatMasker (v4.2.1)^54^ with Dfam (v3.9) and Giraffa tippelskirchi as the reference lineage. The resulting annotation table was converted into BED format using the RM2Bed.py script (https://github.com/Dfam-consortium/RepeatMasker). To calculate the percentage and total length of repeats within non-syntenic regions, we intersected the repeats identified by RepeatMasker with the non-syntenic regions using bedtools.^64^

##### Gene annotation

Gene annotation on the T2T-mGirTip1v1.0 was performed with EviAnn software (v2.0.3).^59^ EviAnn software produces evidence-based genome annotation. It derives gene annotations from aligned and assembled transcriptome sequencing data and protein homology. Illumina RNA-seq and PacBio Kinnex read data from testes tissue of mGirTip1 and cardiac muscle tissue of mGirTip4 were used as transcriptomic evidence, in addition to the blood of mGirTip1 sequenced with PacBio Kinnex. A set of 576,821 mammalian proteins was used as protein homology evidence. Function annotations of protein coding genes were performed by aligning protein sequences to the UniProt-Swissprot database^60^ of functional proteins. Table S4 shows count of protein coding and long non-coding RNA genes, transcripts and proteins for both primary and alternative haplotype assemblies. 98% of all annotated transcripts were assigned function with alignments to UniProt.

Both the genome and the annotation were evaluated for completeness of the gene content using BUSCO^51^ version 5.8.3 with “mammalia” orthologous protein database containing 9,226 amino acid sequences. BUSCO scores for the genome and the annotated proteins are shown in Table S5. Primary and alternative haplotype assemblies show excellent completeness, missing only 0.9% and 3.1% of BUSCOs. Note that while annotation has fewer fragmented BUSCOs (0.4%) compared to the assemblies (0.8%), it has about 0.8 percentage points more missing BUSCOs, which implies that BUSCO software was able to identify homologous matches in the genome, but were not able to identify such matches in the protein sequences.

##### PAR regions

We aligned chrY_pat to chrX_mat using minimap2 with the asm20 preset to identify pseudoautosomal (PAR) regions on the sex chromosomes. The resulting PAF file was filtered to retain long, high-identity alignments (≥10 kb and ≥95% identity), representing PAR homologous segments. These intervals were converted to BED format, retaining only the chrY-derived coordinates, used to mask the corresponding regions on the primary assembly with bedtools maskfasta, producing a PAR-masked version of the reference genome.

To match the orientation of chromosome Y with other ruminant genomes, such as cattle and sheep, we inverted the chromosome so that it begins with the large PAR region followed by the centromere, ensuring that the short arm appears first for consistency.

##### HG002 assembly

HG002 data from the HPRC was used to generate the baseline assembly. HiFi reads were downloaded from https://s3-us-west-2.amazonaws.com/human-pangenomics/index.html?prefix=T2T/HG002/assemblies/polishing/HG002/v1.0/mapping/hifi_revio_pbmay24/hg002v1.0.1_hifi_revio_pbmay24.bam, ONT reads from https://s3-us-west-2.amazonaws.com/human-pangenomics/index.html?prefix=NHGRI_UCSC_panel/HG002/nanopore/ultra-long/03_08_22_R941_HG002_rebasecalling-guppy-6.3.7/03∗_[4-6]∗.fq.gz, and Hi-C data from https://s3-us-west-2.amazonaws.com/human-pangenomics/index.html?prefix=T2T/HG002/assemblies/polishing/HG002/v1.0/mapping/ont_r10_ul_dorado/HG002.HiC_1_S∗fastq.gz. ONT reads were corrected using a pre-release version of hifiasm v0.25.0-r831 hybrid correction inputting both R10 data and HiFi data using the --hifi parameter. Only corrected ONT data was retained and corrected HiFi data was discarded. Verkko HG002 assembly was generated using Verkko (v2.3) with HiFi, ONT corrected data input using the --hifi parameter, ONT R9 + R10 input as --nano and, and Hi-C data input as --hic1/2 parameters. The assembly also used --unitig-abundance 8 due to the high input coverage. Mitochondrial DNA (human-mito-NC_012920.1.fasta.gz), rDNA sequences (human-rdna-KY962518.1.fasta.gz), and EBV sequences (human-ebv-AJ507799.2.fasta.gz) under Verkko library directory were screened during assembly using the --screen-human-contaminants parameter.

The base Verkko assembly directory and associated files were loaded into Verkko-Fillet within a Python environment. ONT reads were aligned back to the base Verkko assembly graph using GraphAligner^41^ (v1.0.19) and were used to fill assembly gaps. A new path file (TSV format) was generated after gap filling and provided to Verkko to construct an updated consensus sequence.

The updated consensus assembly was then reloaded into Verkko-Fillet to identify internal telomeres and missing terminal telomeres. For chromosomes lacking a terminal telomere but containing an internal telomeric sequence, and where extended sequences beyond the internal telomere were supported primarily by ONT or corrected ONT reads, the chromosome ends were trimmed to the internal telomere boundary.

To validate assembly improvements achieved using Verkko-Fillet, we generated k-mer databases from both the assembly and raw sequencing reads using Meryl^61^ (v1.4.1) and calculated per-chromosome QV scores using Merqury^61^ (v1.3). GQC^26^ (v0.1.0) (commit 12c2884f5b7be5b4a2f86401d928d122c6422485) was used to compare the assemblies to HG002 Q100 v1.1^26^ with default parameters.

## Supplemental information

Document S1. Figures S1–S19 and Tables S1–S7

Document S2. Article plus supplemental information

## References

[1] Nurk S., Koren S., Rhie A., Rautiainen M., Bzikadze A.V., Mikheenko A., Vollger M.R., Altemose N., Uralsky L., Gershman A. (2022). The complete sequence of a human genome. Science.

[2] Miga K.H., Koren S., Rhie A., Vollger M.R., Gershman A., Bzikadze A., Brooks S., Howe E., Porubsky D., Logsdon G.A. (2020). Telomere-to-telomere assembly of a complete human X chromosome. Nature.

[3] Rhie A., Nurk S., Cechova M., Hoyt S.J., Taylor D.J., Altemose N., Hook P.W., Koren S., Rautiainen M., Alexandrov I.A. (2023). The complete sequence of a human Y chromosome. Nature.

[4] Liao W.-W., Asri M., Ebler J., Doerr D., Haukness M., Hickey G., Lu S., Lucas J.K., Monlong J., Abel H.J. (2023). A draft human pangenome reference. Nature.

[5] Miga K.H., Wang T. (2021). The Need for a Human Pangenome Reference Sequence. Annu. Rev. Genomics Hum. Genet..

[6] Rhie A., McCarthy S.A., Fedrigo O., Damas J., Formenti G., Koren S., Uliano-Silva M., Chow W., Fungtammasan A., Kim J. (2021). Towards complete and error-free genome assemblies of all vertebrate species. Nature.

[7] Kalbfleisch T.S., McKay S.D., Murdoch B.M., Adelson D.L., Almansa-Villa D., Becker G., Beckett L.M., Benítez-Galeano M.J., Biase F., Casey T. (2024). The Ruminant Telomere-to-Telomere (RT2T) Consortium. Nat. Genet..

[8] Cheng H., Concepcion G.T., Feng X., Zhang H., Li H. (2021). Haplotype-resolved de novo assembly using phased assembly graphs with hifiasm. Nat. Methods.

[9] Ruan J., Li H. (2020). Fast and accurate long-read assembly with wtdbg2. Nat. Methods.

[10] Antipov D., Rautiainen M., Nurk S., Walenz B.P., Solar S.J., Phillippy A.M., Koren S. (2025). Verkko2 integrates proximity-ligation data with long-read De Bruijn graphs for efficient telomere-to-telomere genome assembly, phasing, and scaffolding. Genome Res..

[11] Rautiainen M., Nurk S., Walenz B.P., Logsdon G.A., Porubsky D., Rhie A., Eichler E.E., Phillippy A.M., Koren S. (2023). Telomere-to-telomere assembly of diploid chromosomes with Verkko. Nat. Biotechnol..

[12] Formenti G., Rhie A., Walenz B.P., Thibaud-Nissen F., Shafin K., Koren S., Myers E.W., Jarvis E.D., Phillippy A.M. (2022). Merfin: improved variant filtering, assembly evaluation and polishing via k-mer validation. Nat. Methods.

[13] Mastoras M., Asri M., Brambrink L., Hebbar P., Kolesnikov A., Cook D.E., Nattestad M., Lucas J., Won T.S., Chang P.-C. (2025). Highly accurate assembly polishing with DeepPolisher. Genome Research.

[14] Mc Cartney A.M., Shafin K., Alonge M., Bzikadze A.V., Formenti G., Fungtammasan A., Howe K., Jain C., Koren S., Logsdon G.A. (2022). Chasing perfection: validation and polishing strategies for telomere-to-telomere genome assemblies. Nat. Methods.

[15] Gnerre S., Lander E.S., Lindblad-Toh K., Jaffe D.B. (2009). Assisted assembly: how to improve a de novo genome assembly by using related species. Genome Biol..

[16] Bayat A., Deshpande N.P., Wilkins M.R., Parameswaran S. (2020). Fast Short Read De-Novo Assembly Using Overlap-Layout-Consensus Approach. IEEE/ACM Trans. Comput. Biol. Bioinform..

[17] Li Z., Chen Y., Mu D., Yuan J., Shi Y., Zhang H., Gan J., Li N., Hu X., Liu B. (2012). Comparison of the two major classes of assembly algorithms: overlap–layout–consensus and de-bruijn-graph. Brief. Funct. Genomics.

[18] Cao M.D., Nguyen S.H., Ganesamoorthy D., Elliott A.G., Cooper M.A., Coin L.J.M. (2017). Scaffolding and completing genome assemblies in real-time with nanopore sequencing. Nat. Commun..

[19] Burton J.N., Adey A., Patwardhan R.P., Qiu R., Kitzman J.O., Shendure J. (2013). Chromosome-scale scaffolding of de novo genome assemblies based on chromatin interactions. Nat. Biotechnol..

[20] Garg S., Rautiainen M., Novak A.M., Garrison E., Durbin R., Marschall T. (2018). A graph-based approach to diploid genome assembly. Bioinforma. Oxf. Engl..

[21] Cheng H., Jarvis E.D., Fedrigo O., Koepfli K.-P., Urban L., Gemmell N.J., Li H. (2022). Haplotype-resolved assembly of diploid genomes without parental data. Nat. Biotechnol..

[22] Koren S., Rhie A., Walenz B.P., Dilthey A.T., Bickhart D.M., Kingan S.B., Hiendleder S., Williams J.L., Smith T.P.L., Phillippy A.M. (2018). De novo assembly of haplotype-resolved genomes with trio binning. Nat. Biotechnol..

[23] Ghurye J., Rhie A., Walenz B.P., Schmitt A., Selvaraj S., Pop M., Phillippy A.M., Koren S. (2019). Integrating Hi-C links with assembly graphs for chromosome-scale assembly. PLoS Comput. Biol..

[24] Koren S., Walenz B.P., Berlin K., Miller J.R., Bergman N.H., Phillippy A.M. (2017). Canu: scalable and accurate long-read assembly via adaptive k-mer weighting and repeat separation. Genome Res..

[25] Volpe E., Colantoni A., Corda L., Di Tommaso E., Pelliccia F., Ottalevi R., Guarracino A., Licastro D., Faino L., Capulli M. (2025). The reference genome of the human diploid cell line RPE-1. Nat. Commun..

[26] Hansen N.F., Dwarshuis N., Ji H.J., Rhie A., Loucks H., Logsdon G.A., Vollger M.R., Storer J.M., Kim J., Adam E. (2026). A complete diploid human genome benchmark for personalized genomics. Cell.

[27] Yoshitake K., Shirasawa K., Kojima K.K., Asakawa S., Tanaka N., Kurokochi H. (2025). Nearly telomere-to-telomere genome assembly of the L. *edodes* diploid genome. Fungal Genet. Biol..

[28] Sarashetti P., Lipovac J., Jia Q., Wang L., Li Z., Vrcek L., Chen Y., Yao F., Sia Y.Y., Lin D. (2025). A Complete Telomere-to-Telomere Diploid Reference Genome for South Asian Population. Preprint at bioRxiv.

[29] Godec T., Beier S., Rodriguez-Granados N.Y., Sasidharan R., Abdelhakim L., Teige M., Usadel B., Gruden K., Petek M. (2025). Haplotype-resolved genome assembly of the tetraploid potato cultivar Désirée. Sci. Data.

[30] Serra Mari R., Schrinner S., Finkers R., Ziegler F.M.R., Arens P., Schmidt M.H.-W., Usadel B., Klau G.W., Marschall T. (2024). Haplotype-resolved assembly of a tetraploid potato genome using long reads and low-depth offspring data. Genome Biol..

[31] Cheng H., Asri M., Lucas J., Koren S., Li H. (2024). Scalable telomere-to-telomere assembly for diploid and polyploid genomes with double graph. Nat. Methods.

[32] Zhu Y., Watson C., Safonova Y., Pennell M., Bankevich A. (2025). CloseRead: a tool for assessing assembly errors in immunoglobulin loci applied to vertebrate long-read genome assemblies. Genome Biol..

[33] Chen Y., Zhang Y., Wang A.Y., Gao M., Chong Z. (2021). Accurate long-read de novo assembly evaluation with Inspector. Genome Biol..

[34] Rabanal F.A., Gräff M., Lanz C., Fritschi K., Llaca V., Lang M., Carbonell-Bejerano P., Henderson I., Weigel D. (2022). Pushing the limits of HiFi assemblies reveals centromere diversity between two Arabidopsis thaliana genomes. Nucleic Acids Res..

[35] Navrátilová P., Toegelová H., Tulpová Z., Kuo Y.-T., Stein N., Doležel J., Houben A., Šimková H., Mascher M. (2022). Prospects of telomere-to-telomere assembly in barley: Analysis of sequence gaps in the MorexV3 reference genome. Plant Biotechnol. J..

[36] Chu C., Li X., Wu Y. (2019). GAPPadder: a sensitive approach for closing gaps on draft genomes with short sequence reads. BMC Genom..

[37] Midekso F.D., Yi G. (2022). RFfiller: a robust and fast statistical algorithm for gap filling in draft genomes. PeerJ.

[38] Schmeing S., Robinson M.D. (2023). Gapless provides combined scaffolding, gap filling, and assembly correction with long reads. Life Sci. Alliance.

[39] Mastoras M., Asri M., Brambrink L., Hebbar P., Kolesnikov A., Cook D.E., Nattestad M., Lucas J., Won T.S., Chang P.-C. (2025). Highly accurate assembly polishing with DeepPolisher. Genome Res..

[40] Zhang E., Coombe L., Wong J., Warren R.L., Birol I. (2025). GoldPolish-target: targeted long-read genome assembly polishing. BMC Bioinf..

[41] Rautiainen M., Marschall T. (2020). GraphAligner: rapid and versatile sequence-to-graph alignment. Genome Biol..

[42] Thorvaldsdóttir H., Robinson J.T., Mesirov J.P. (2013). Integrative Genomics Viewer (IGV): high-performance genomics data visualization and exploration. Brief. Bioinform..

[43] Wick R.R., Schultz M.B., Zobel J., Holt K.E. (2015). Bandage: interactive visualization of de novo genome assemblies. Bioinforma. Oxf. Engl..

[44] Rautiainen M. (2024). Ribotin: automated assembly and phasing of rDNA morphs. Bioinforma. Oxf. Engl..

[45] Huang L., Nesterenko A., Nie W., Wang J., Su W., Graphodatsky A.S., Yang F. (2008). Karyotype evolution of giraffes (Giraffa camelopardalis) revealed by cross-species chromosome painting with Chinese muntjac (Muntiacus reevesi) and human (Homo sapiens) paints. Cytogenet. Genome Res..

[46] Xie Z., Zhang J., Yang T., Bo X., Fu Z., Yang F., Ye F., Ni M. (2026). Filtering of artificial chimeric reads generated by ligation preparation method of nanopore sequencing. iScience.

[47] Cernohorska H., Kubickova S., Kopecna O., Kulemzina A.I., Perelman P.L., Elder F.F.B., Robinson T.J., Graphodatsky A.S., Rubes J. (2013). Molecular cytogenetic insights to the phylogenetic affinities of the giraffe (Giraffa camelopardalis) and pronghorn (Antilocapra americana). Chromosome Res..

[48] Farré M., Li Q., Darolti I., Zhou Y., Damas J., Proskuryakova A.A., Kulemzina A.I., Chemnick L.G., Kim J., Ryder O.A. (2019). An integrated chromosome-scale genome assembly of the Masai giraffe (Giraffa camelopardalis tippelskirchi). GigaScience.

[49] Yoo D., Rhie A., Hebbar P., Antonacci F., Logsdon G.A., Solar S.J., Antipov D., Pickett B.D., Safonova Y., Montinaro F. (2025). Complete sequencing of ape genomes. Nature.

[50] Vollger M.R., Dishuck P.C., Sorensen M., Welch A.E., Dang V., Dougherty M.L., Graves-Lindsay T.A., Wilson R.K., Chaisson M.J.P., Eichler E.E. (2019). Long-read sequence and assembly of segmental duplications. Nat. Methods.

[51] Seppey M., Manni M., Zdobnov E.M. (2019). BUSCO: Assessing Genome Assembly and Annotation Completeness. Methods Mol. Biol. Clifton NJ *1962*. Methods Mol. Biol..

[52] Liu C., Gao J., Cui X., Li Z., Chen L., Yuan Y., Zhang Y., Mei L., Zhao L., Cai D. (2021). A towering genome: Experimentally validated adaptations to high blood pressure and extreme stature in the giraffe. Sci. Adv..

[53] Porubsky D., Guitart X., Yoo D., Dishuck P.C., Harvey W.T., Eichler E.E. (2025). SVbyEye: a visual tool to characterize structural variation among whole-genome assemblies. Bioinformatics.

[54] Tarailo-Graovac M., Chen N. (2009). Using RepeatMasker to Identify Repetitive Elements in Genomic Sequences. Current Protocols in Bioinformatics.

[55] Abdennur N., Fudenberg G., Flyamer I.M., Galitsyna A.A., Goloborodko A., Imakaev M., Venev S.V. (2024). Pairtools: From sequencing data to chromosome contacts. PLoS Comput. Biol..

[56] Wang X.-B., Lu H.-W., Liu Q.-Y., Li A.-L., Zhou H.-L., Zhang Y., Zhu T.-Q., Ruan J. (2024). An effective strategy for assembling the sex-limited chromosome. GigaScience.

[57] Carey S.B., Lovell J.T., Jenkins J., Leebens-Mack J., Schmutz J., Wilson M.A., Harkess A. (2022). Representing sex chromosomes in genome assemblies. Cell Genom..

[58] Frohlich J., Kubickova S., Musilova P., Cernohorska H., Muskova H., Vodicka R., Rubes J. (2017). Karyotype relationships among selected deer species and cattle revealed by bovine FISH probes. PLoS One.

[59] Zimin A.V., Puiu D., Pertea M., Yorke J.A., Salzberg S.L. (2026). Efficient evidence-based genome annotation with EviAnn. Nat. Methods.

[60] The UniProt Consortium (2007). The universal protein resource (UniProt). Nucleic Acids Res..

[61] Rhie A., Walenz B.P., Koren S., Phillippy A.M. (2020). Merqury: reference-free quality, completeness, and phasing assessment for genome assemblies. Genome Biol..

[62] Li, H. (2025). seqtk github. https://github.com/lh3/seqtk.

[63] Li H. (2018). Minimap2: pairwise alignment for nucleotide sequences. Bioinforma. Oxf. Engl..

[64] Quinlan A.R., Hall I.M. (2010). BEDTools: a flexible suite of utilities for comparing genomic features. Bioinforma. Oxf. Engl..

[65] Jain C., Rhie A., Zhang H., Chu C., Walenz B.P., Koren S., Phillippy A.M. (2020). Weighted minimizer sampling improves long read mapping. Bioinforma. Oxf. Engl..

[66] Poplin R., Chang P.-C., Alexander D., Schwartz S., Colthurst T., Ku A., Newburger D., Dijamco J., Nguyen N., Afshar P.T. (2018). A universal SNP and small-indel variant caller using deep neural networks. Nat. Biotechnol..

[67] Li H., Durbin R. (2009). Fast and accurate short read alignment with Burrows-Wheeler transform. Bioinforma. Oxf. Engl..

[68] Abdennur N., Mirny L.A. (2020). Cooler: scalable storage for Hi-C data and other genomically labeled arrays. Bioinforma. Oxf. Engl..

[69] Jain C., Koren S., Dilthey A., Phillippy A.M., Aluru S. (2018). A fast adaptive algorithm for computing whole-genome homology maps. Bioinforma. Oxf. Engl..

[70] McKinney, W. (2010). Data Structures for Statistical Computing in Python. SciPy 2010, 10.25080/Majora-92bf1922-00a

[71] GFA Format Specification Working Group (2025).

[72] Hagberg A., Swart P.J., Schult D.A. (2007).

[73] Li H., Handsaker B., Wysoker A., Fennell T., Ruan J., Homer N., Marth G., Abecasis G., Durbin R., 1000 Genome Project Data Processing Subgroup (2009). The Sequence Alignment/Map format and SAMtools. Bioinforma. Oxf. Engl..

[74] Genovese G., Rockweiler N.B., Gorman B.R., Bigdeli T.B., Pato M.T., Pato C.N., Ichihara K., McCarroll S.A. (2024). BCFtools/liftover: an accurate and comprehensive tool to convert genetic variants across genome assemblies. Bioinforma. Oxf. Engl..

