# Supplementary material for: Finishing a complete giraffe genome from telomere to telomere with Verkko-Fillet
Source: Cell Genom. 2026 Aug 6;6(8):101279. doi: 10.1016/j.xgen.2026.101279 (PMC13477062; doi:10.1016/j.xgen.2026.101279)
Supplement: Supplementary file 1 [file mmc1.pdf]

Cell Genomics, Volume *6*

Supplemental information

Finishing a complete giraffe genome

from telomere to telomere with Verkko-Fillet

Juhyun Kim, Benjamin D. Rosen, Sarah E. Fumagalli, Kristen L. Kuhn, Amy Long, Jeffrey J. Schoenebeck, Heather Schwartz, Lan Wu-Cavener, Aleksey V. Zimin, Douglas R. Cavener, Timothy P.L. Smith, Adam M. Phillippy, Sergey Koren, and Arang Rhie

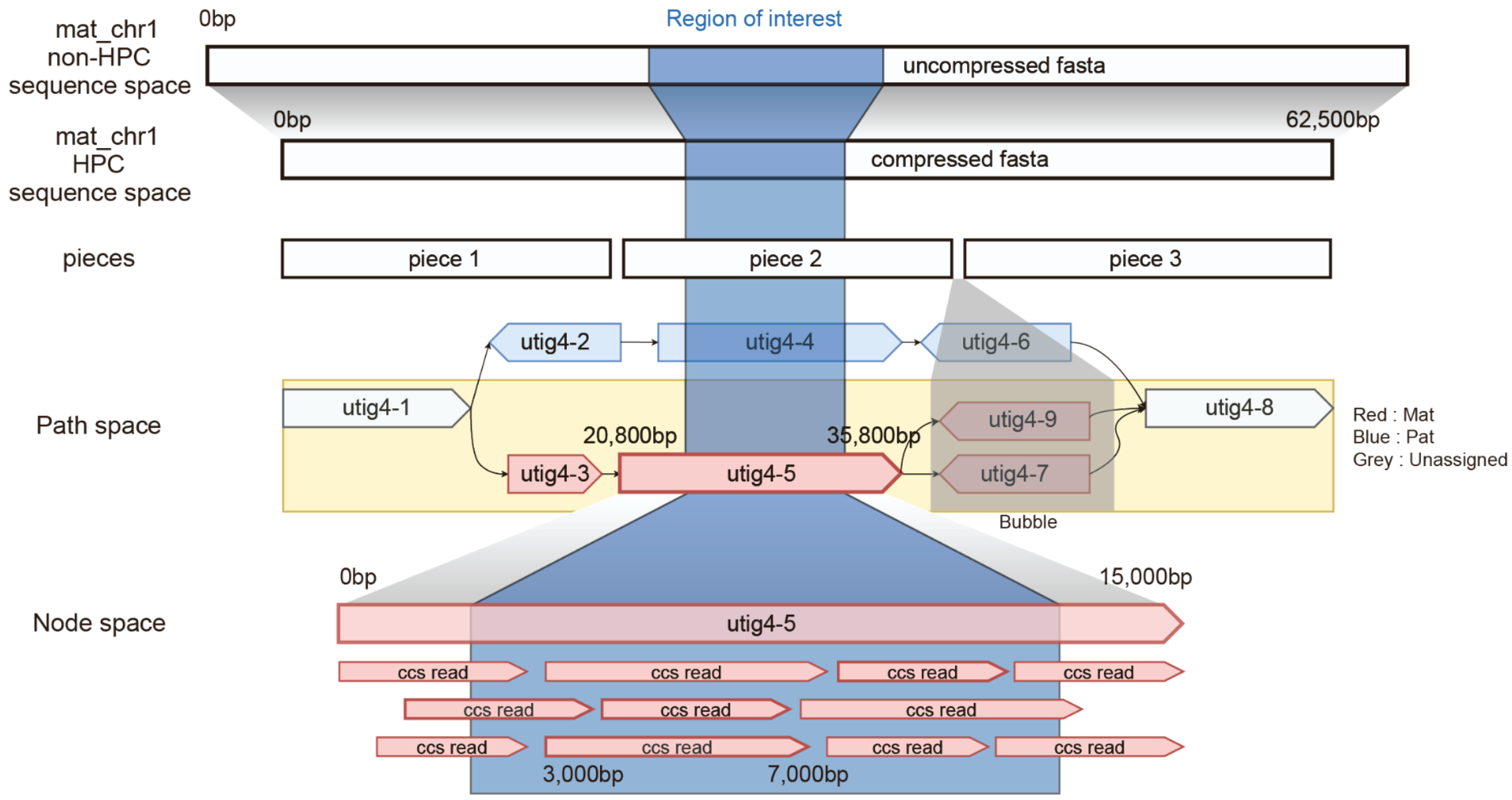

Figure S1. Verkko assembly structure, related to Figure 1.
The Verkko assembly is organized into multiple layers. The bottom-most layer shows the node space, which records the reads used and later contributes to the consensus. Directly above the node space is the path space, where the order, connectivity, and orientation of nodes define how contigs are generated. For example, the maternal (red) haplotype is assembled from the path utig4-1 (+), utig4-3 (+), utig4-5 (+), [gap], utig4-8 (+). The next layer is the piece space, in which paths are split when the node walk becomes ambiguous (i.e., at a gap) and are represented as separate pieces, as illustrated between utig4-5 and utig4-8. Finally, all pieces are assembled in homopolymer-compressed (HPC) sequence, concatenated with gaps. The HPC sequences are later reversed in the final consensus step of the assembly process.

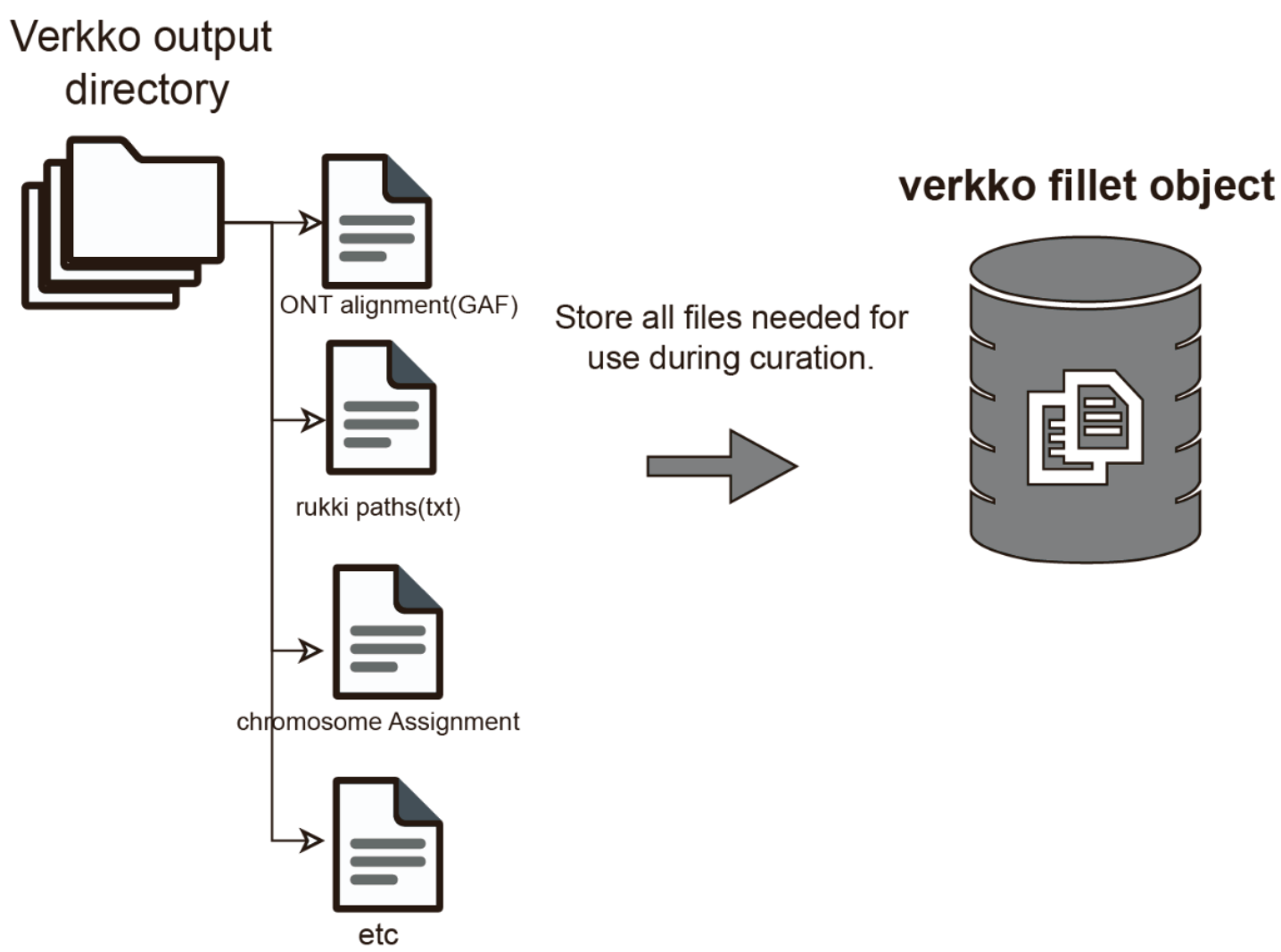

vf-obj.gaps

gapId	contig	gap	fixdPath
gapid_0	chr5_mat	[utig4-2639+, [N5000N:ambig_path], utig4-2652+]	utig4-2639+, utig4-2652+
gapid_1	chr5_mat	[utig4-70+, [N212780N:tangle], utig4-100+]	utig4-70+, utig4-99-, utig4-96-, utig4-97+, utig4-96-, utig4-98+, utig4-100+
gapid_2	chr5_mat	[utig4-100+, [N5000N:ambig_path], utig4-2421+]	utig4-100+, utig4-2421+, utig4-2421+, utig4-2421+
gapid_3	chr11_mat	[utig4-1130-, [N5000N:ambig_path], utig4-1082+]	utig4-1130-, utig4-1131+, utig4-333-, utig4-331+, utig4-334+, utig4-1087-, utig4-1083-, utig4-1082+
gapid_4	chr11_mat	[utig4-370+, [N100000N:scaffold], utig4-2178+]	utig4-370+, utig4-373+, utig4-2179-, utig4-2176-, utig4-2178+
gapid_5	chr10_mat	[utig4-1957+, [N5000N:ambig_path], utig4-2581-]	utig4-1957+, utig4-1961+, utig4-2583-, utig4-2581-

vf-obj.node

node	len	mat-kmer	pat-kmer	hap	ONT support
utig4-626	910848	29	36	ambiguous	10682
utig4-629	3058	0	0	ambiguous	225
utig4-630	8464	0	16	ambiguous	195
utig4-2613	525203	6	28	ambiguous	6049

vf-obj.paths

contig	path
chr10_mat	utig4-1795+,utig4-2059-,utig4-2060+, …, ,utig4-1672-,utig4-294-,utig4-290-,utig4-292+
chr10_pat	utig4-2062-,utig4-2059-,utig4-2061+, … , ,utig4-1672-,utig4-293-,utig4-290-,utig4-291+

Figure S2. The structure of the Verkko-Fillet object, related to Figure 1.
The Verkko-Fillet object parses key outputs from the Verkko assembly directory, including paths, gaps, nodes, edges, and haplotype information. Gap filling is performed by integrating multiple data sources, such as ONT, HiFi, and Hi-C alignments. All attributes stored in the Verkko-Fillet object can be accessed in a dataframe format. In this figure, we show the gaps, nodes, and paths attributes, which represent key components of the Verkko-Fillet.

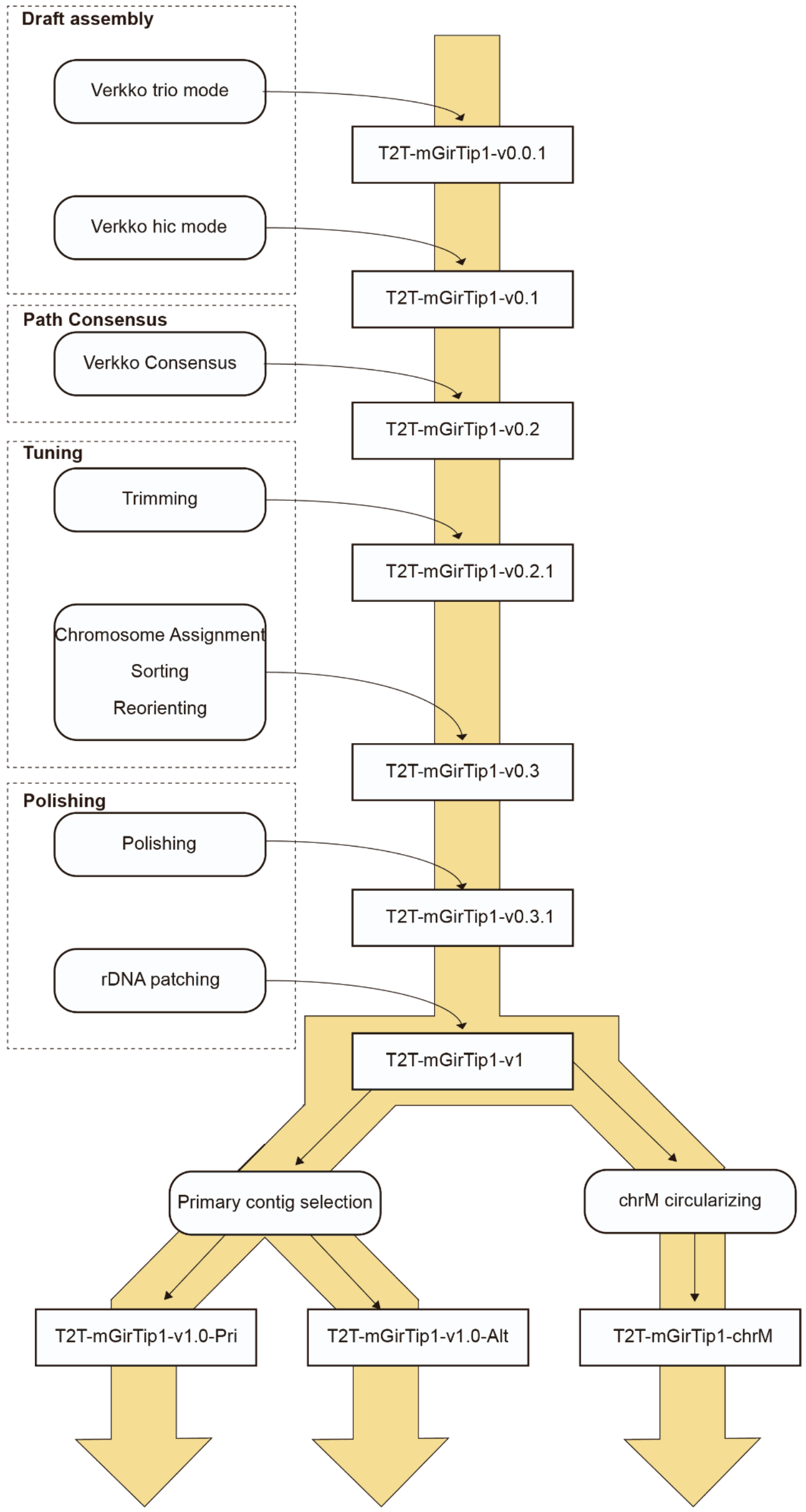

Figure S3. Versioning of T2T-mGirTip1 and associated workflow, related to Figure 1.
The giraffe genome assemblies were generated following the workflow described in Figure 1. The final primary, alternate, and circularized mitochondrial genome assemblies are provided as FASTA files ready for genome submission.

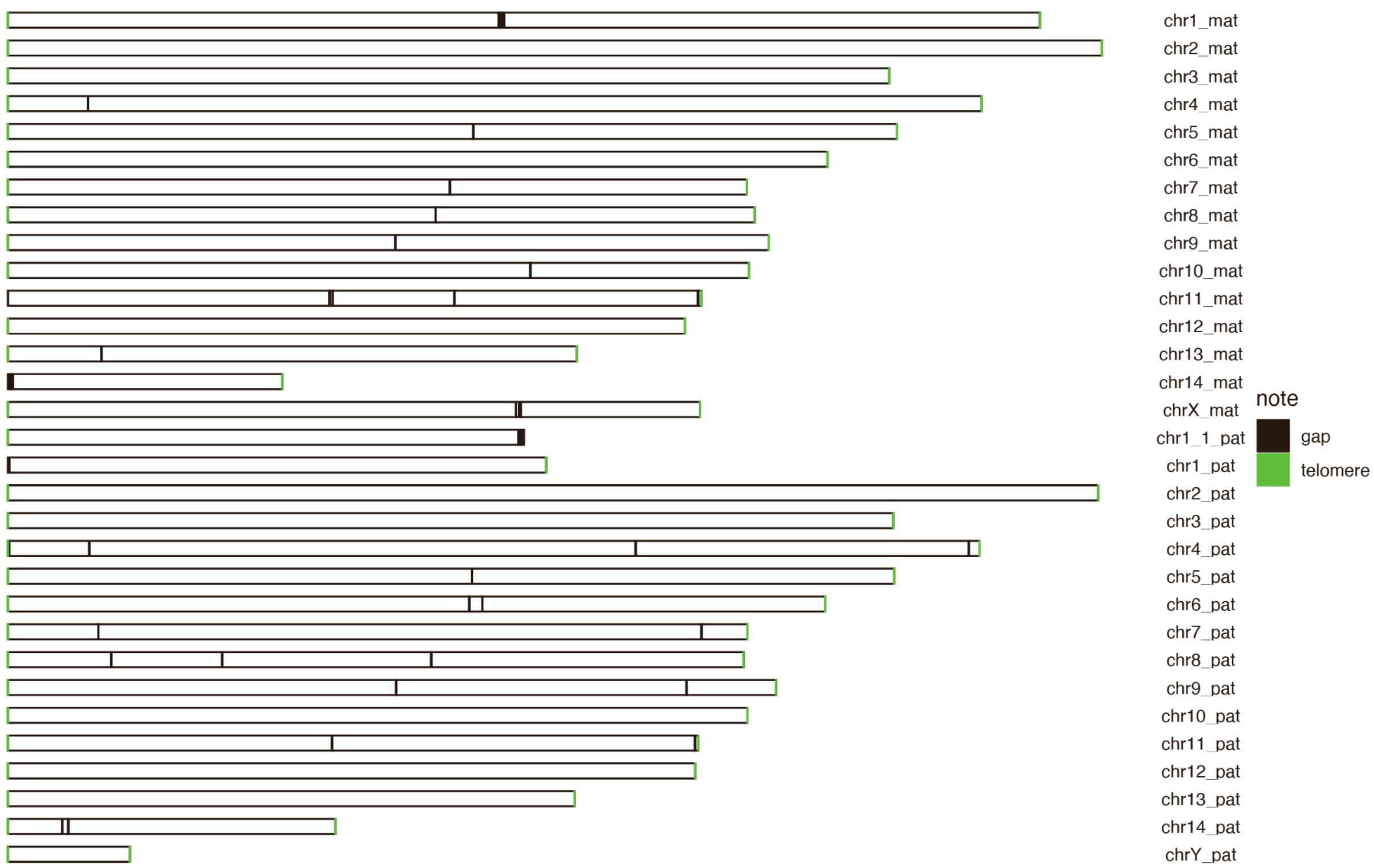

Figure S4. Ideogram of T2T-mGirTip1v0.1 with gap and telomere annotations, related to Figure 2. Black indicates gaps, and green marks telomere regions identified in T2T-mGirTip1v0.1. Chr1 paternal haplotype consists of two separate contigs (chr1_1_pat and chr1_pat), with only one telomere present at each contig end, and is nearly half the size of chr1 of the maternal haplotype.

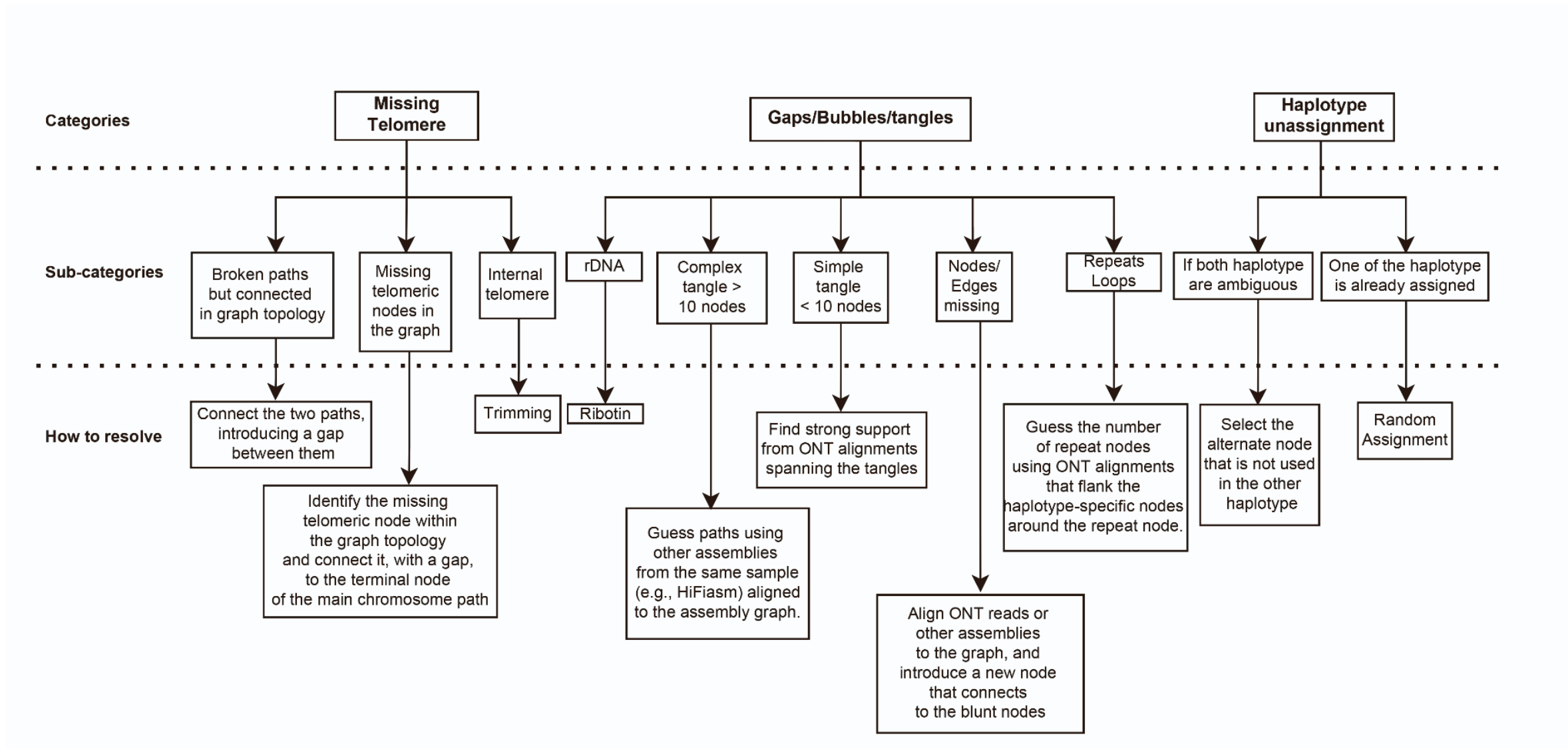

Figure S5. Decision-making tree for resolving tangles or correcting contigs, related to Figure 3. We defined three major categories—missing telomeres, tangles, and haplotype unassignment—each with their own subcategories. For every subcategory, we outline the corresponding strategy used to resolve the issue, providing a systematic framework for addressing assembly errors.

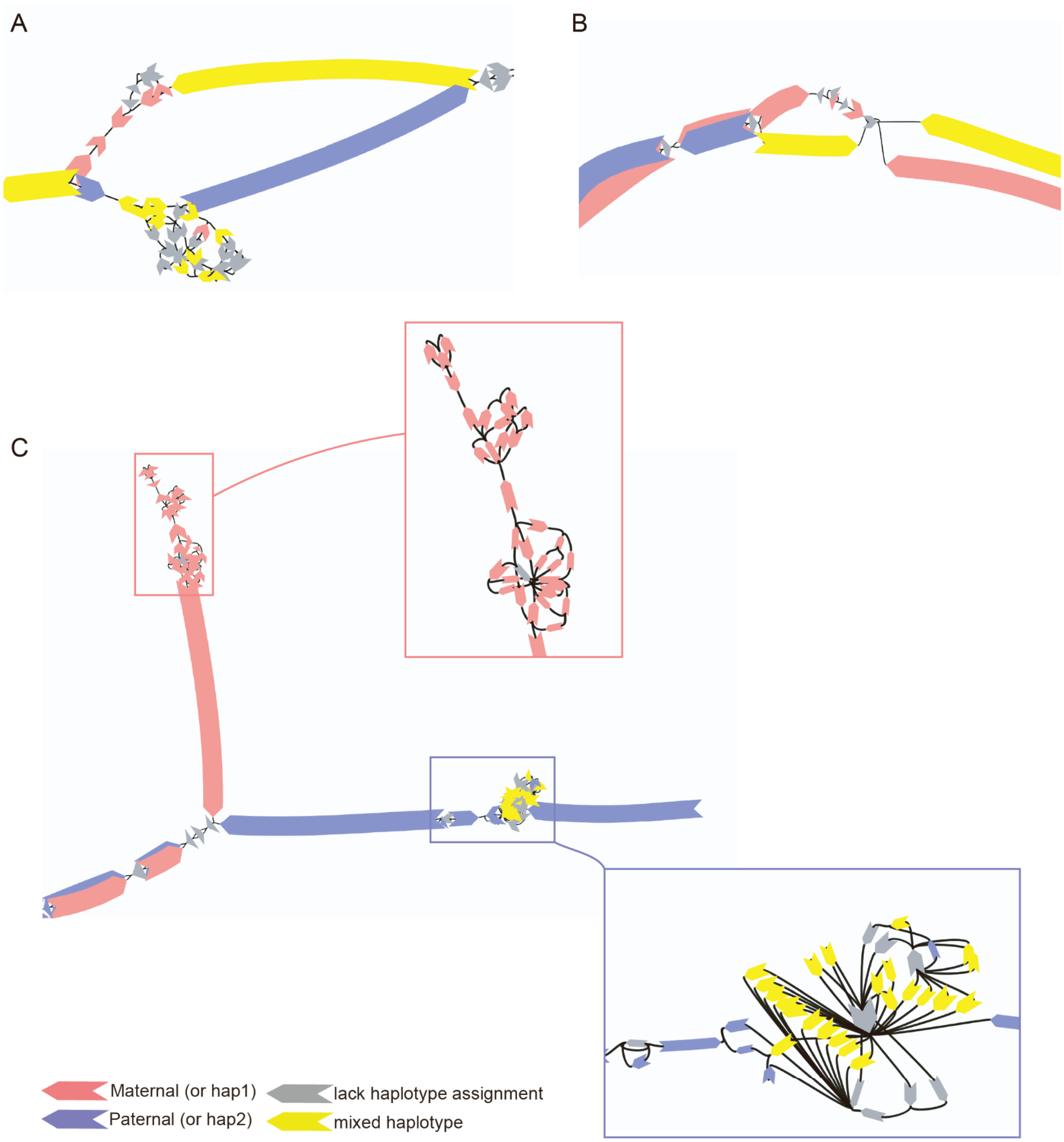

Figure S6. rDNA tangles in the Verkko graph, related to Figure 3.
rDNA tangles in the diploid Verkko graph for chromosomes 8 (A), 11 (B), and 14 (C). Nodes are colored by haplotype: maternal nodes are shown in pink and paternal nodes in blue. Gray indicates nodes lacking parent-specific *k*-mers, and yellow indicates nodes with mixed parent *k*-mers.

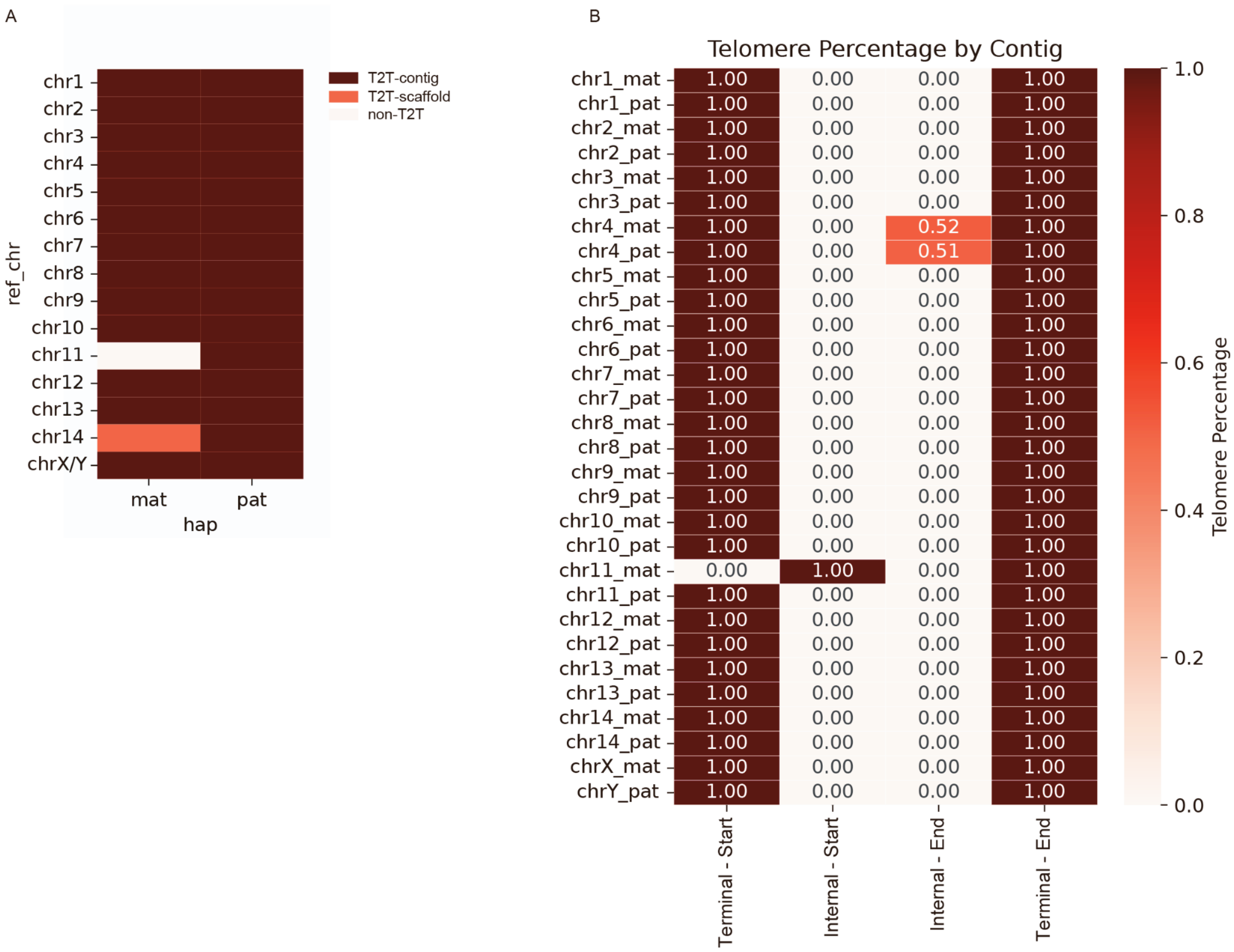

Figure S7. T2T statistics and telomere distribution in T2T-mGirTip1v0.1 (after gap filling), related to Figure4.
(A) After path correction, including gap filling and contig joining, one contig (chr11_mat) lacks a telomere at its end. Another contig (chr14_mat) is the only T2T scaffold, with telomeres at both ends but containing a gap. (B) Chr11_mat shows a high portion of telomeric sequences found in the internal region (18,000 bps away from the terminus).

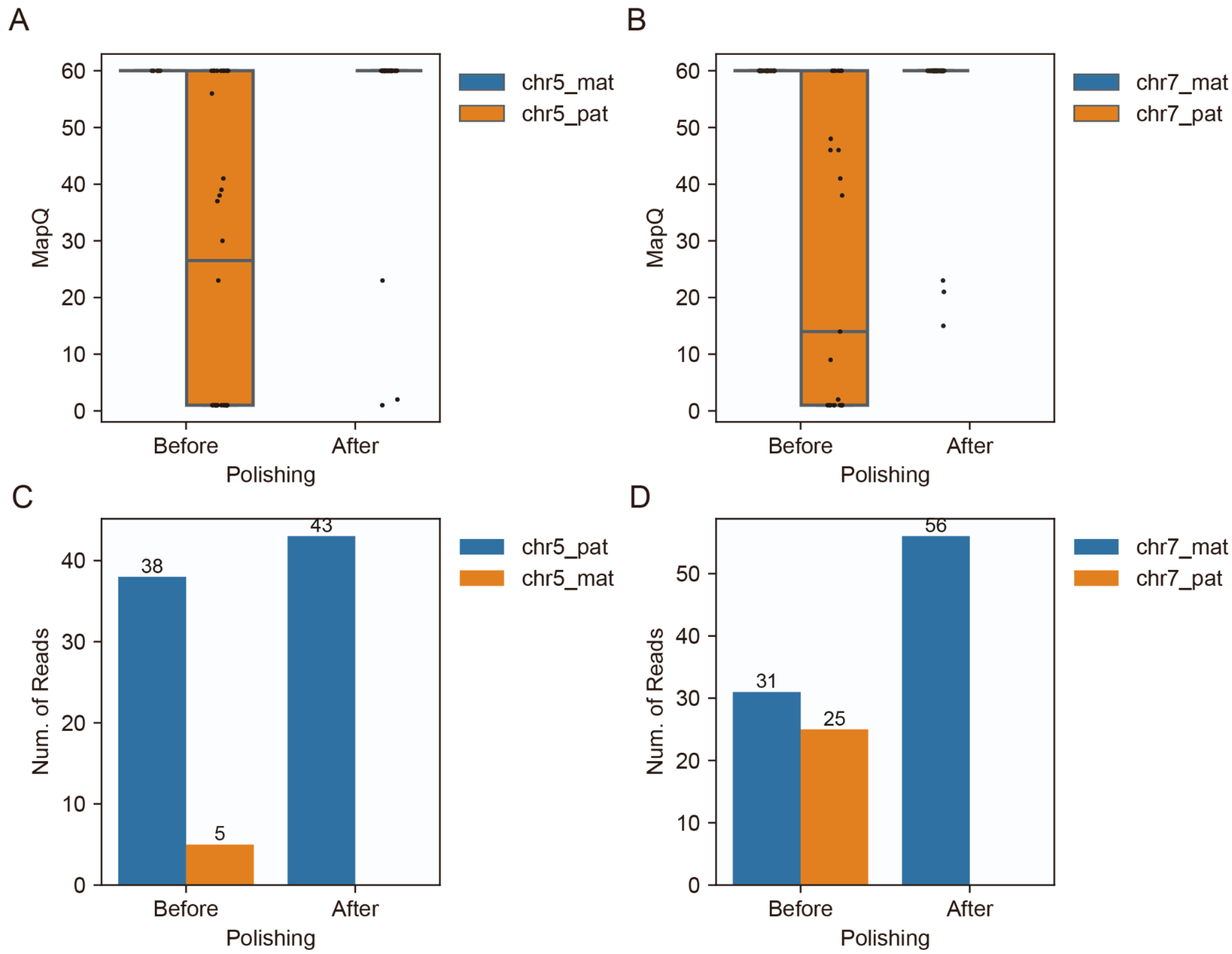

Figure S8. Comparison of mapping quality for reads that are more accurately mapped to the polished genome, stratified by chromosome and haplotype, related to Figure 4.

Reads were extracted from the regions on the polished genome shown in Figures 4E (A) and 4F (B), and their corresponding alignments in the unpolished genome were identified. Mapping qualities of the reads are presented in panels A and B. Panels C and D (for Figure 4E and 4F, respectively) show the number of reads mapped to each chromosome and haplotype.

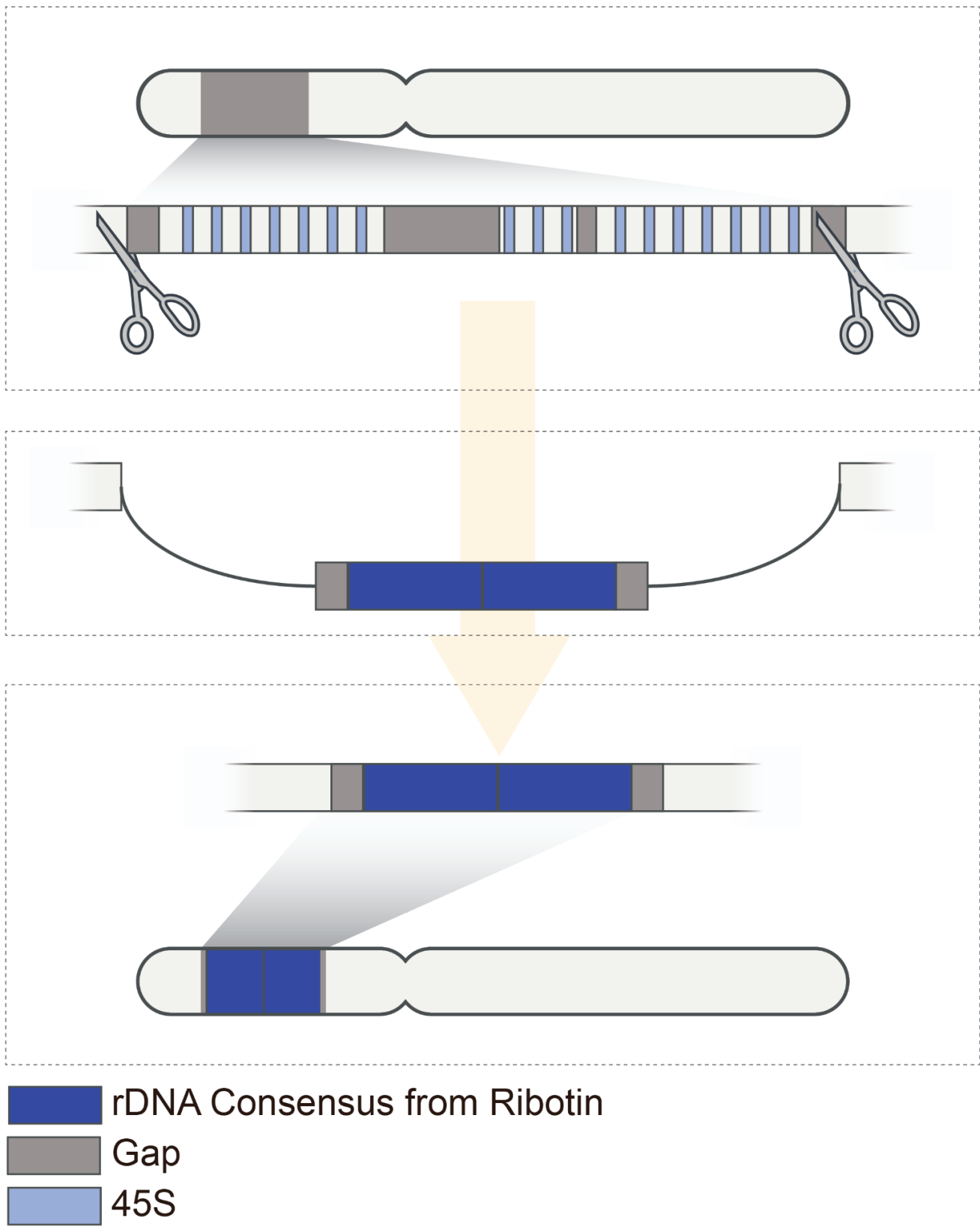

Figure S9. rDNA patching feature in Verkko-Fillet using rDNA consensus, related to Figure 1F. The workflow illustrates the process of patching rDNA regions. rDNA clusters often appear as mosaics of gaps and sequences, sometimes with multiple gaps within a single region. To address this, we provide a workflow that replaces the entire problematic rDNA cluster on a contig with a set of rDNA consensus sequences generated by Ribotin when no rDNA units are found outside the gap flanking sequences. In this case, the rDNA consensus sequences were derived from rDNA nodes within the graph. Two units are concatenated and inserted to bridge the rDNA junction. This approach ensures that intact rDNA units are included, which can be particularly valuable for genome alignment in downstream analyses. Because the exact copy number of the complete rDNA unit is not known, the rDNA consensus is placed in surrounded with two gaps in the main contig.

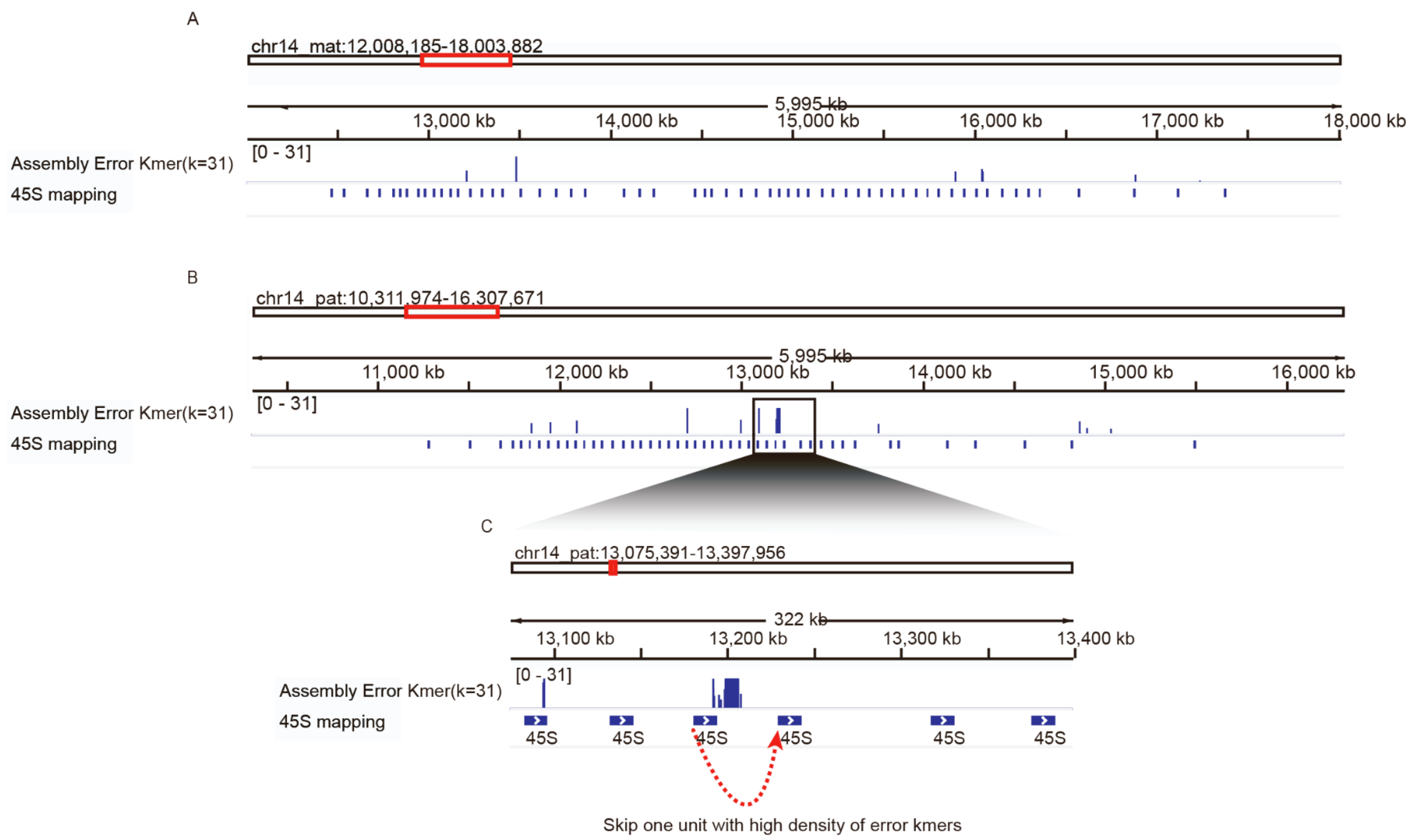

Figure S10. Error k-mer density in the rDNA region on chr14 after polishing, related to Figure 5. (A) rDNA (45S mapping) regions on the chr14 maternal haplotype. No high density of local error *k*-mers are observed. (B) rDNA regions on the chr14 paternal haplotype. One unit exhibits a high density of error *k*-mers. (C) IGV zoom-in view of the boxed region in panel B. The red dotted arrow indicates the site where the assembly was rejoined after removing the erroneous unit.

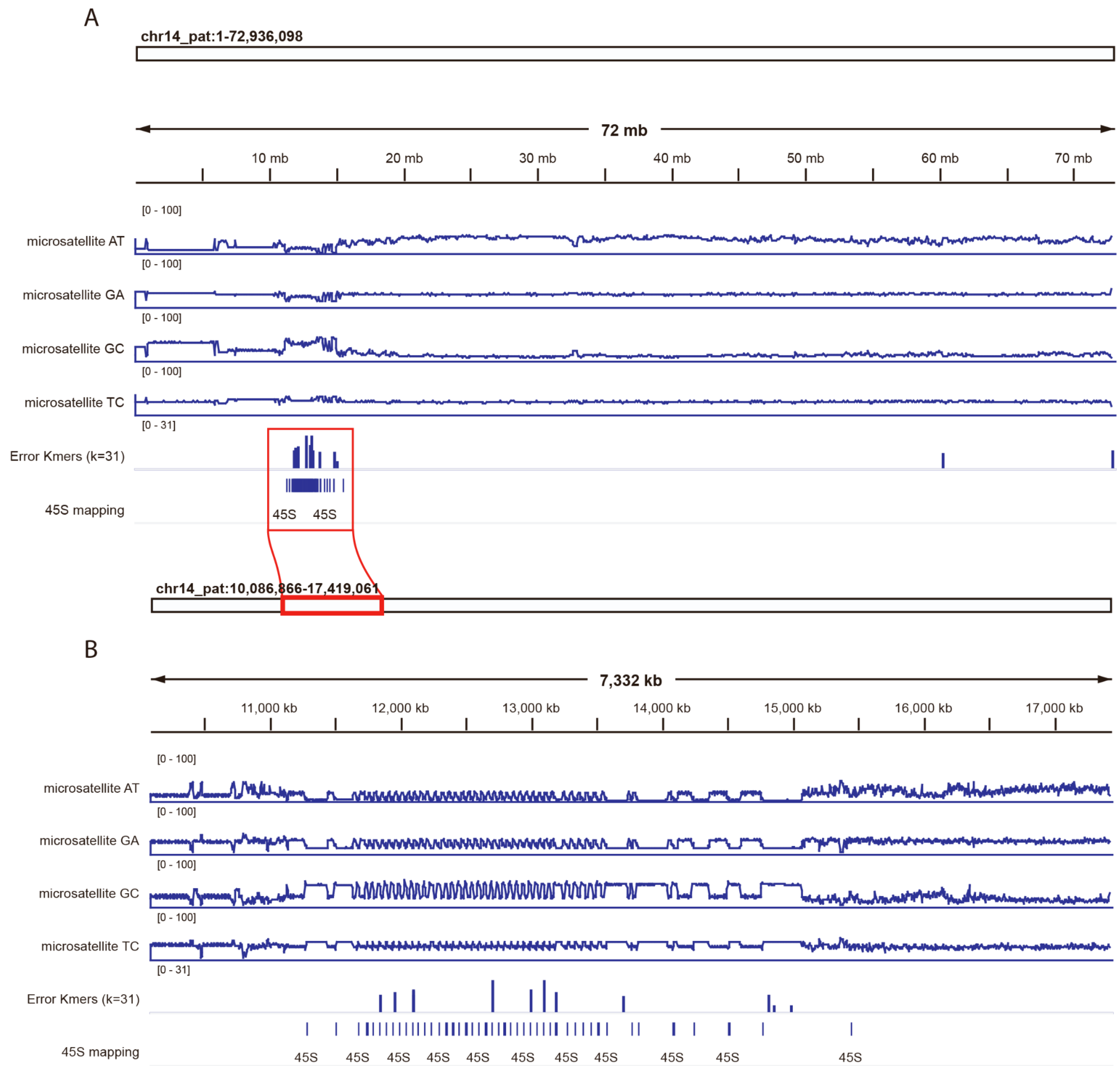

Figure S11. Error *k*-mer clusters around the rDNA array on the paternal haplotype of chromosome 14 after skipping one unit shown in Figure S10, related to Figure 5.
The assembly error *k*-mers (*k*=31) for the whole chr14_pat are shown in (A), with a zoomed-in view of the highlighted rDNA region shown in (B) along with the 45S annotation.

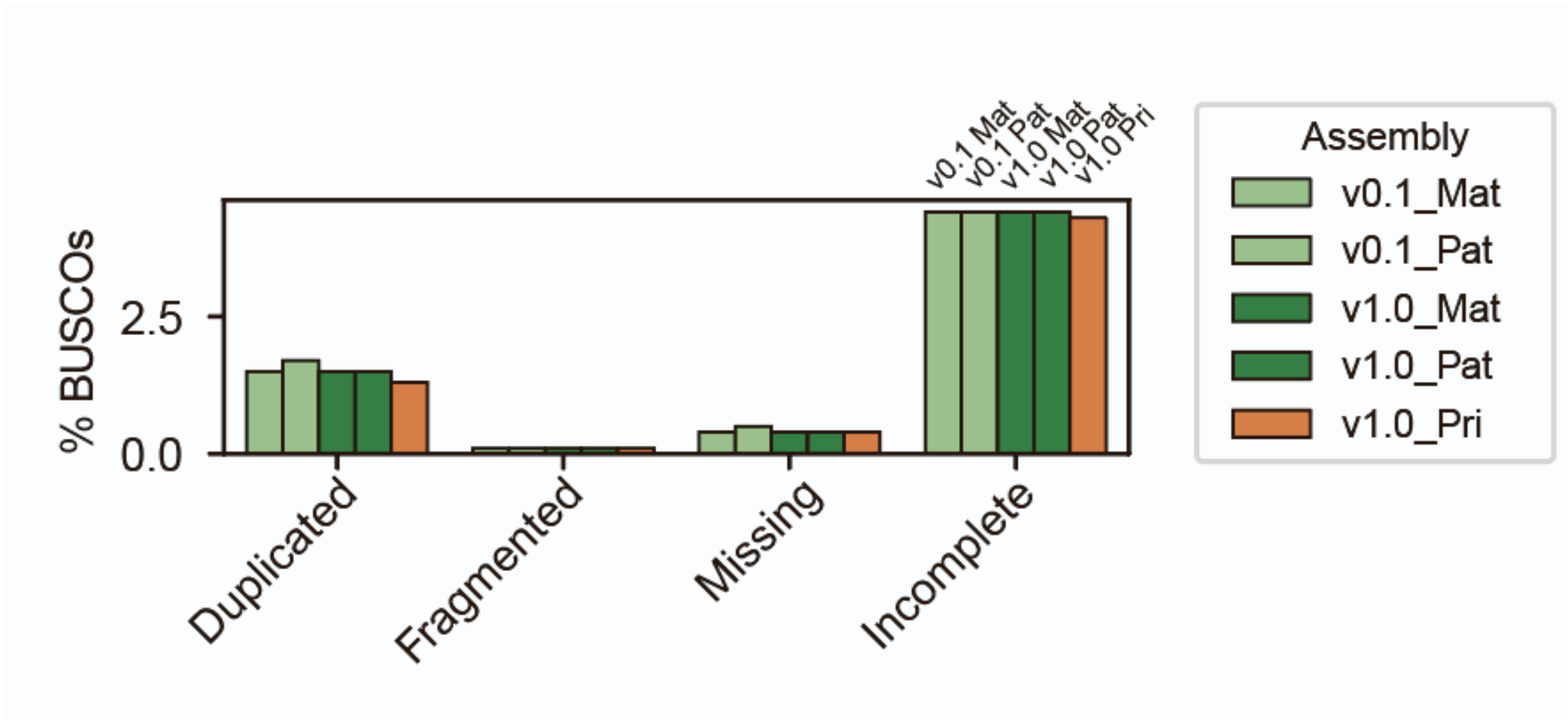

Figure S12. BUSCO scores percentages excluding the "Single Copy" category, related to Figure 5. This comparison shows BUSCO results using the cetartiodactyla_odb10 database, excluding the "Single Copy" category. "Incomplete" indicates predictions containing internal stop codons in Miniprot gene predictions.

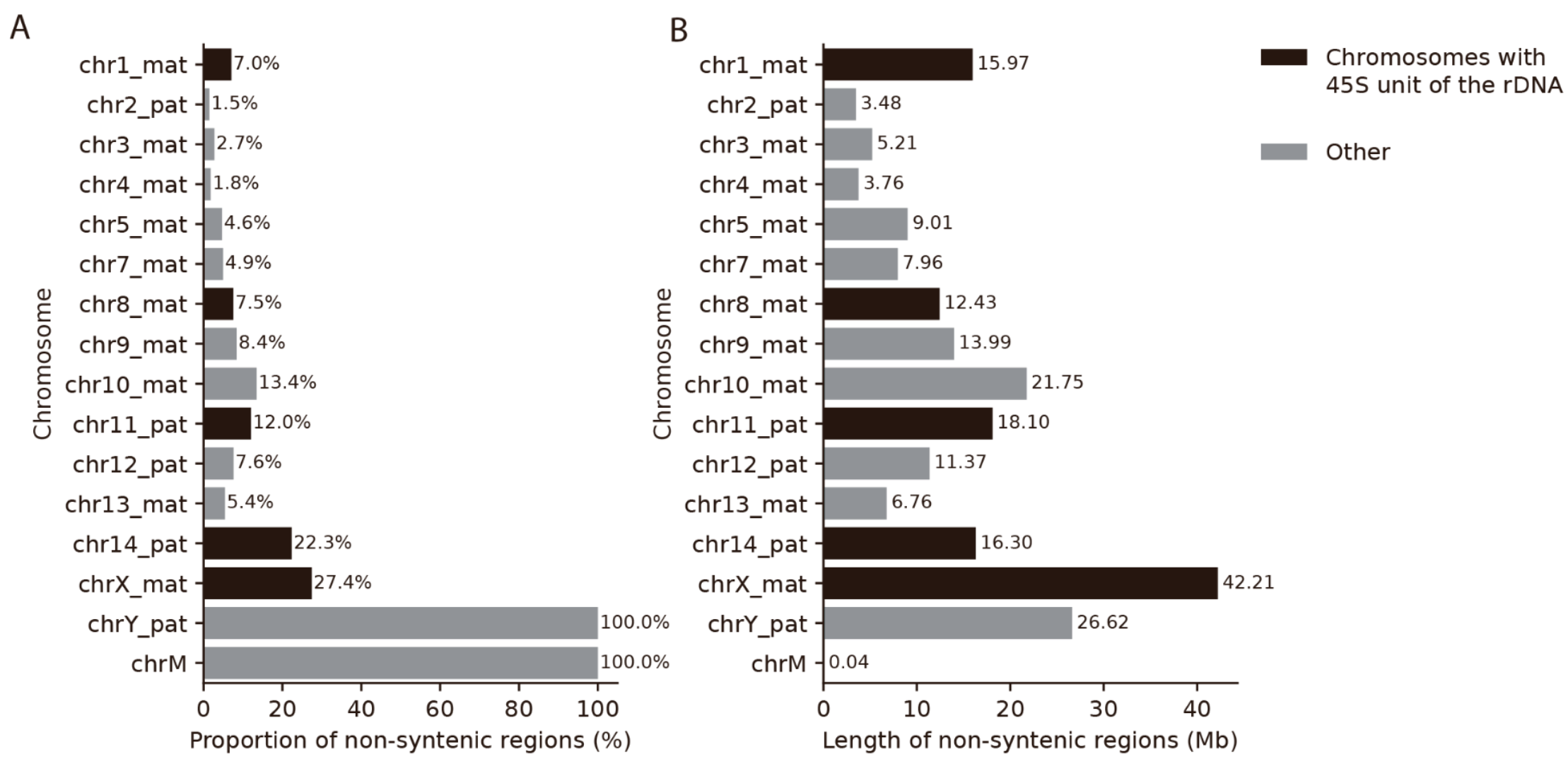

Figure S13. Length and proportion of non-syntenic regions for each chromosome in v1.0-Pri compared to ASM1759144v1, related to Figure 6.

Non-syntenic region are defined as unmappable region when aligning T2T-mGirTip1v1.0-Pri assembly to ASM1759144v1.Chromosomes with >85% identity to the human 45S rDNA unit are highlighted in black. (A) Proportion of non-syntenic regions. (B) Total length of non-syntenic regions in Mbp.

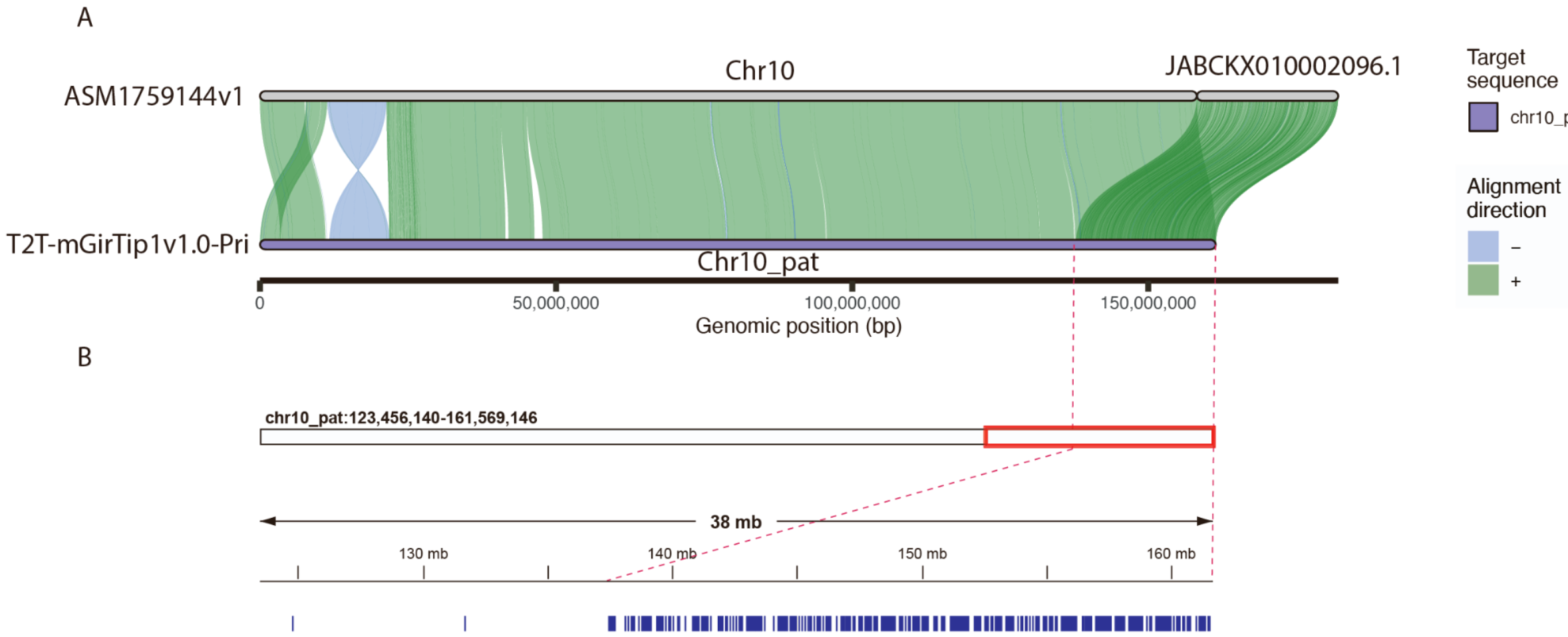

Figure S14. Detailed distribution of gaps in ASM1759144v1 and non-syntenic regions in mGirTip1 on chr10 and chr12, related to Figure 6.

In Figure 6A, some regions appear syntenic between the two assemblies but contain large gaps in ASM1759144v1 or correspond to non-syntenic regions in mGirTip1 at the same genomic location. This reflects a limitation of visualizing a high density of features within a small space. (A) The terminal region of chr10 of T2T-mGirTip1.v1.0-Pri exhibits high synteny not only with the chr10 chromosome from ASM1759144v1, but also with the unplaced contig JABCKX010002096.1. (B) Distribution of non-syntenic regions on chr10 in T2T-mGirTip1v1.0-Pri, showing numerous non-syntenic blocks clustered near the chromosome end, which appear as a single large block in Figure 6A.

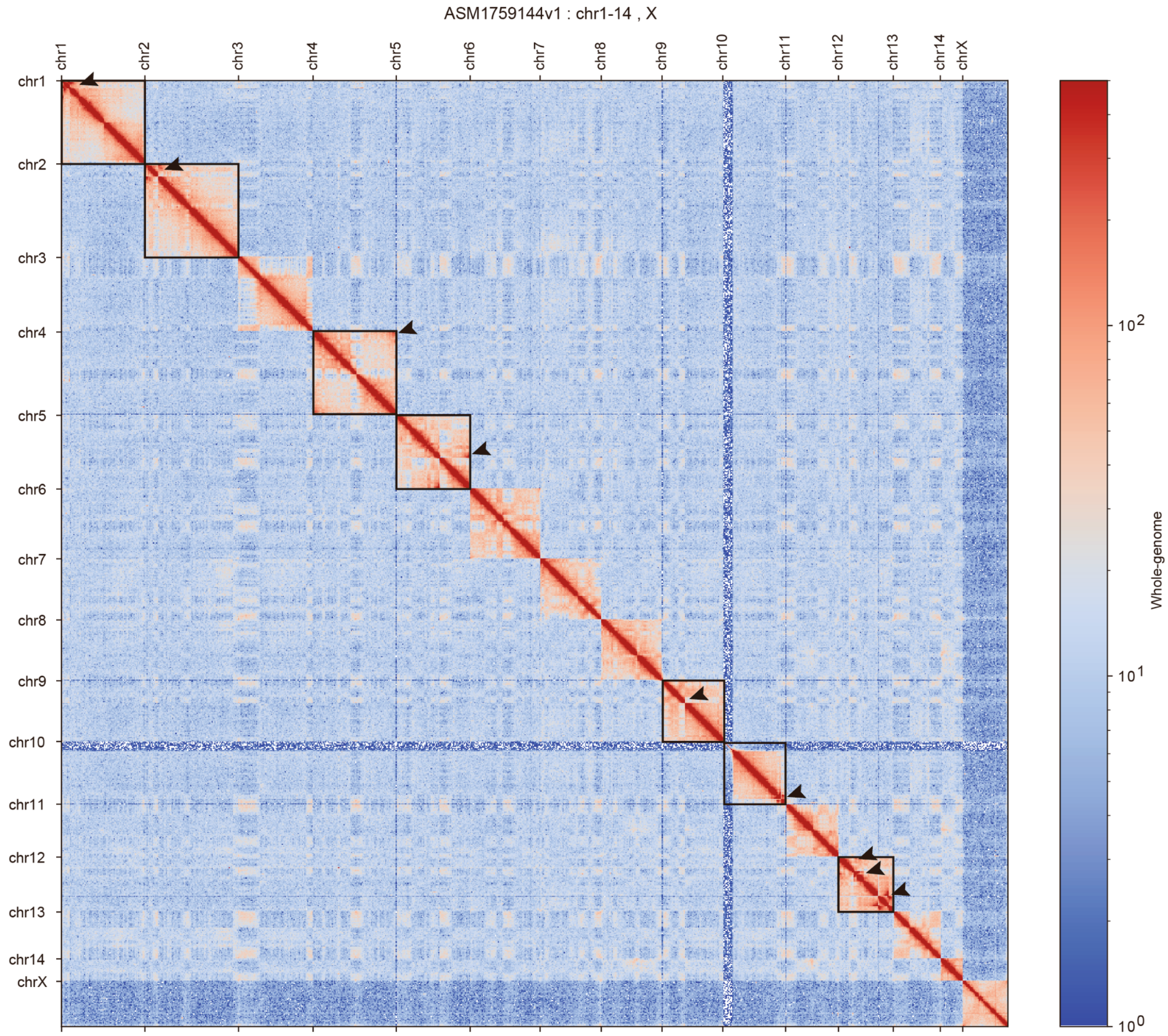

Figure S15. Whole-genome Hi-C contact map of mGirTip1 aligned to ASM1759144v1, related to Figure 6.

The Hi-C contact map reveals large structural differences, including detectable inversions on chr1, 2, 4, 9, and 10. A more detailed zoom-in of these regions is provided in Figure S17. Because ASM1759144v1 lacks chrY and chrM, only the 14 autosomes and chrX are shown here. Other short unplaced scaffolds are not displayed. Black boxes highlight the chromosomes identified as having inversions in Figure 6, and arrows indicate long-range interactions within the chromosomes.

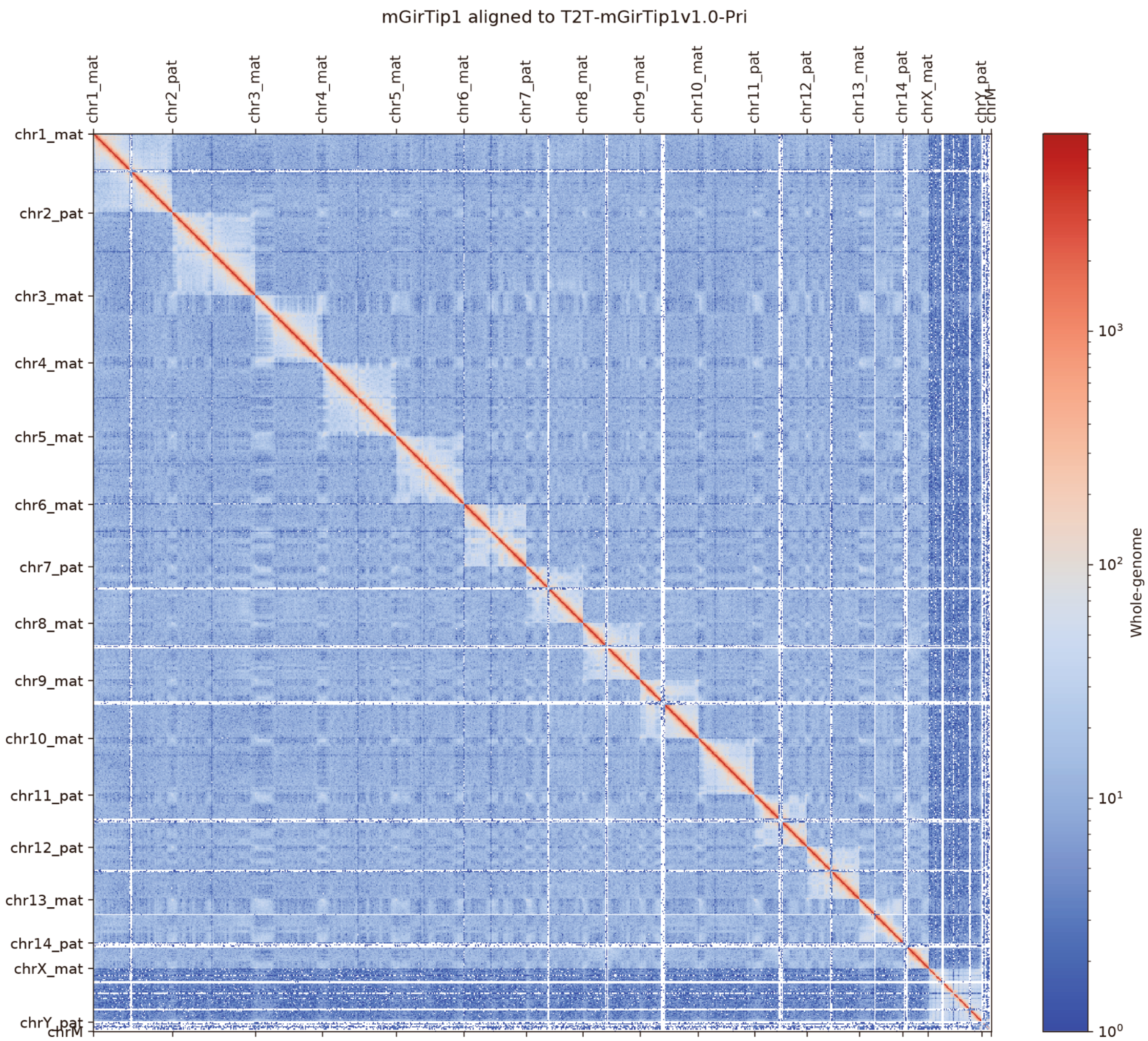

Figure S16. Whole-genome Hi-C contact map of mGirTip1 aligned to the T2T-mGirTip1v1.0 Primary assembly, related to Figure 6.
The Hi-C contact map of T2T-mGirTip1v1.0-Pri shows no long-range interaction across the whole genome. All 14 autosomes, the 2 sex chromosomes, and chrM are displayed.

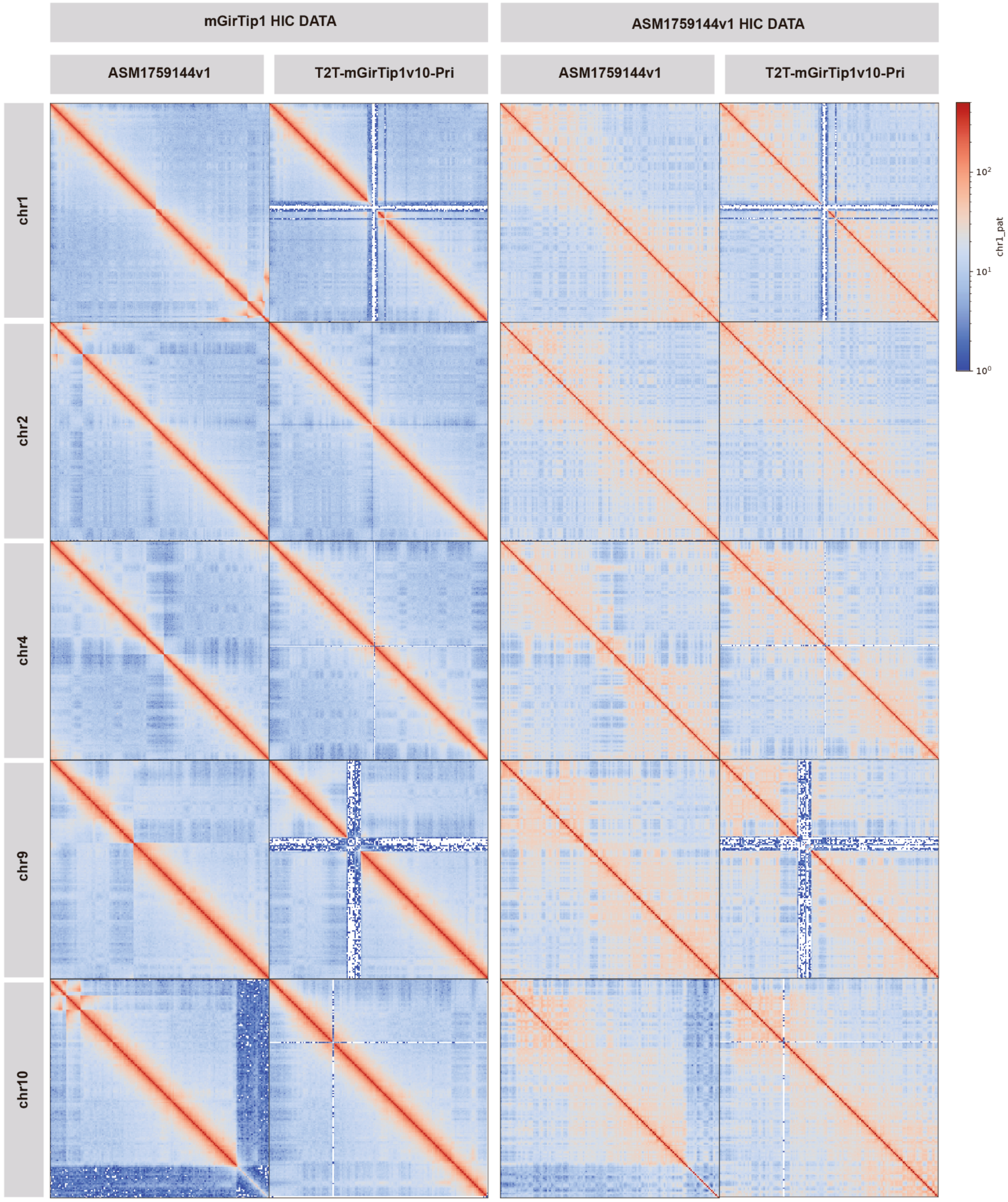

Figure S17. Large structural errors in the ASM1759144v1 assembly, related to Figure 6.
Hi-C contact maps are shown for the full regions of contigs that exhibit large structural discrepancies in Figure 6A. The two left columns display Hi-C data from mGirTip1v1, whereas the two right columns display Hi-C data from ASM1759144v1. Within each dataset, contact maps are presented using both ASM1759144v1 and v1.0-Pri as references.

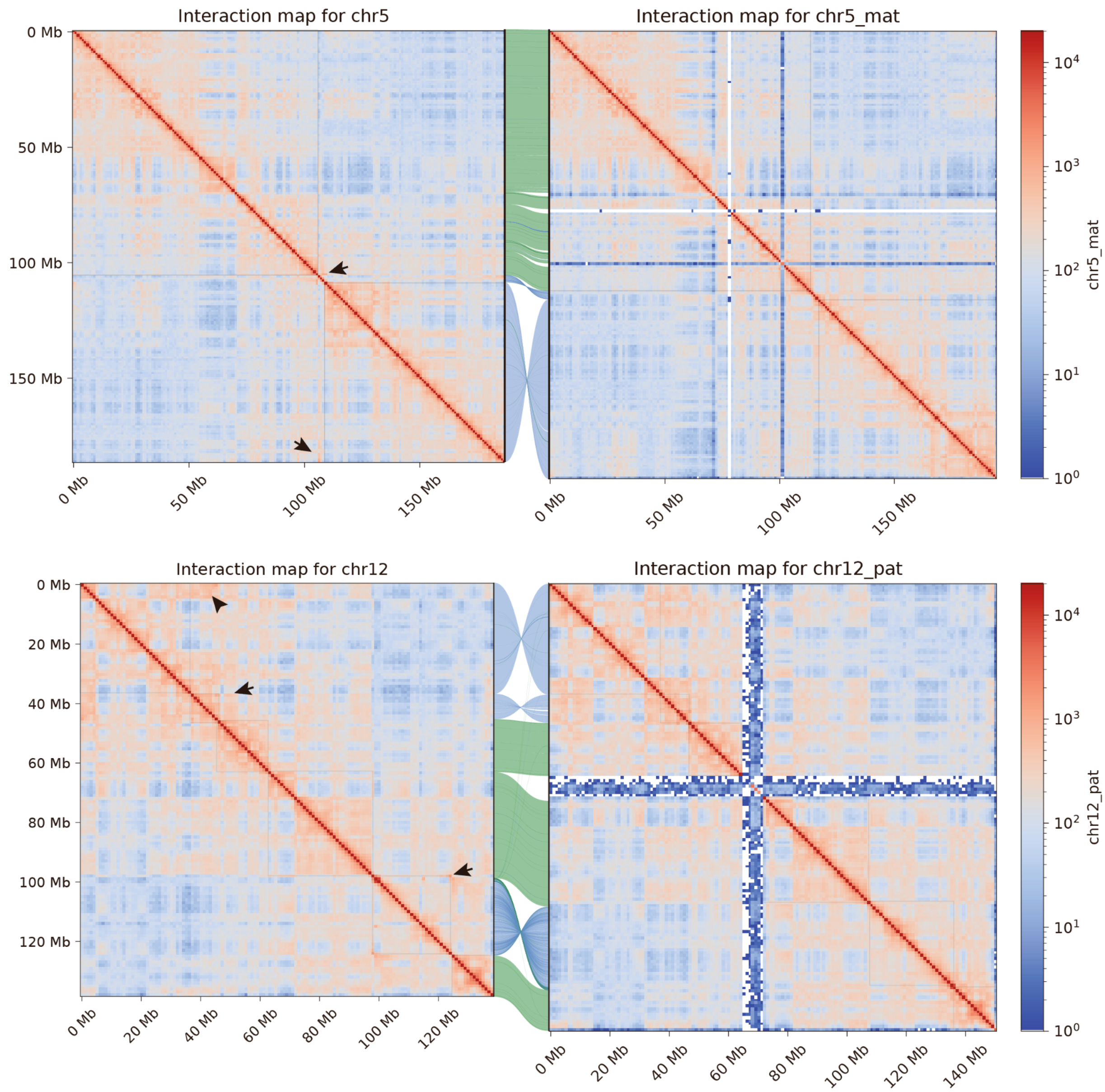

Figure S18. Hi-C contact map of ASM1759144v1 data for chr5 and chr12, related to Figure 6. This figure corresponds to the same region shown in Figures 6G and 6H and presents Hi-C data from ASM1759144v1 aligned to ASM1759144v1 (left) and T2T-mGirTip1v1.0-Pri (right). Arrows in the ASM1759144v1 panel (left) indicate long-range interactions. Overall, these signals are less intense than those observed in the mGirTip1 Hi-C data shown in Figure 6, likely due to lower sequencing coverage.

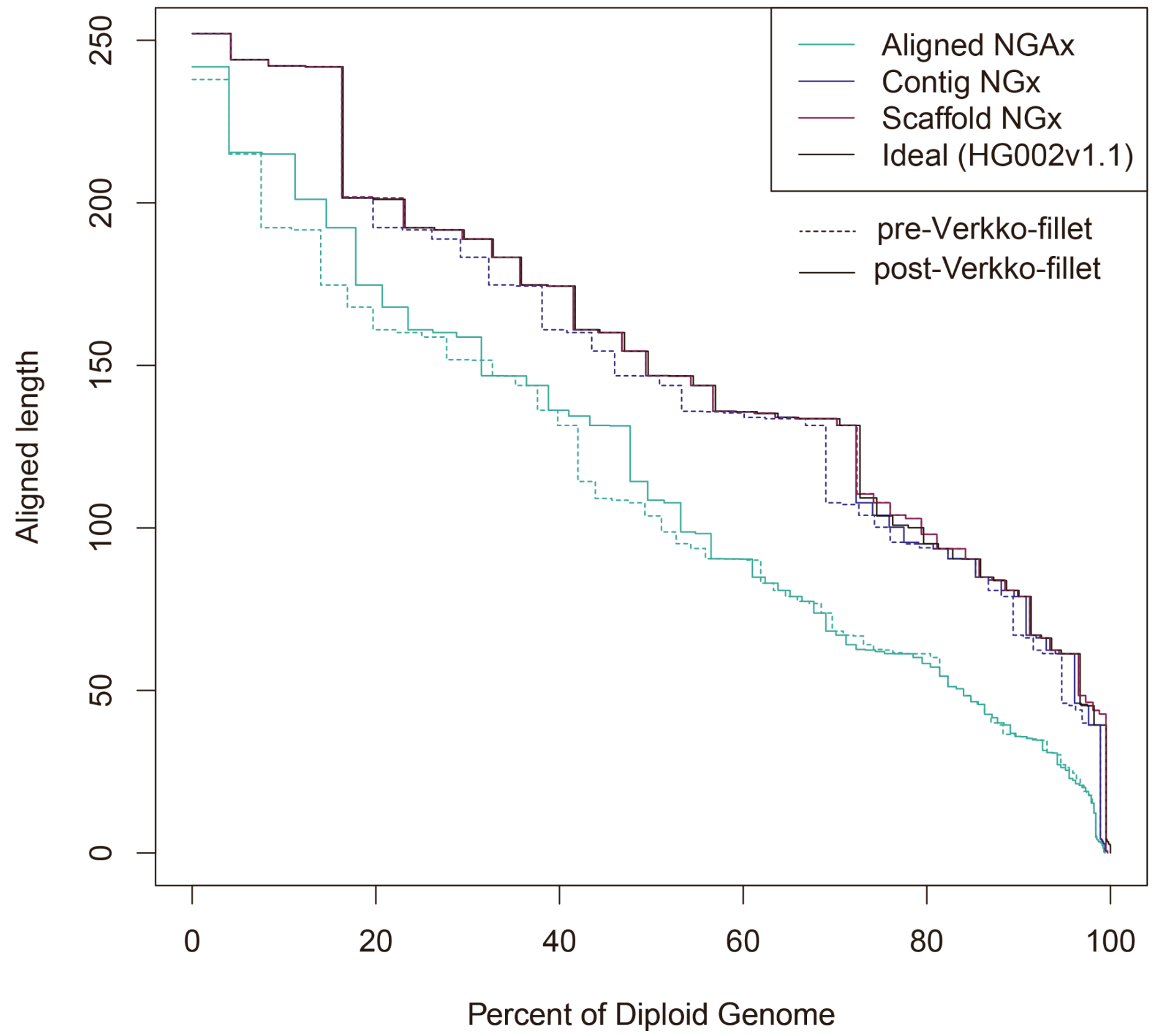

Figure S19. Comparison of assembly quality relative to the HG002 Q100 genome, related to Figure 5.

Assembly contiguity metrics (contig NGx and scaffold NGx) and auNGA statistics, which stands for the area under the NGAx curve, were calculated by aligning each pre- (dotted line) and post- (solid line) Verkko-Fillet assembly to the T2T-HG002v1.1 reference genome.

Table S1. Quality improvements in each T2T-mGirTip1 assembly version using the Verkko-Fillet workflow. QV for chr1_pat is shown for the two contigs before joining to a chromosome, related to Figure 5.

	T2T-mGirTip1v0.1			T2T-mGirTip1v0.2			T2T-mGirTip1v1.0			
	QV	TEL	GAP	QV	TEL	GAP	QV	TEL	GAP	Primary
chr1_mat	67.2146	pq	6	69.4814	pq	0	75.6412	pq	0	*
chr2_mat	73.1596	pq	0	73.1596	pq	0	77.4927	pq	0	
chr3_mat	72.9163	pq	0	72.9163	pq	0	80.9469	pq	0	*
chr4_mat	68.3565	pq	1	72.0424	pq	0	78.1482	pq	0	*
chr5_mat	70.1927	pq	1	72.6101	pq	0	80.0137	pq	0	*
chr6_mat	72.22	pq	0	72.22	pq	0	79.9626	pq	0	*
chr7_mat	67.0505	pq	1	67.7105	pq	0	76.7466	pq	0	
chr8_mat	68.3886	pq	1	69.9227	pq	0	77.5403	pq	0	*
chr9_mat	69.0864	pq	1	71.6048	pq	0	78.4392	pq	0	*
chr10_mat	69.471	pq	1	71.5066	pq	0	79.7661	pq	0	*
chr11_mat	60.5087	q	4	67.8271	pq	0	70.2007	pq	0	
chr12_mat	73.6913	pq	0	73.6913	pq	0	79.2899	pq	0	
chr13_mat	68.4702	pq	1	71.0893	pq	0	81.389	pq	0	*
chr14_mat	61.7228	q	2	63.2882	pq	1	64.0459	pq	1	
chr1_pat	66.5345<br>65.8425	p/q	3/1	68.4283	pq	0	72.3769	pq	0	
chr2_pat	70.8588	pq	0	70.8588	pq	0	79.8754	pq	0	*
chr3_pat	71.1894	pq	0	71.1894	pq	0	77.5672	pq	0	
chr4_pat	64.8251	pq	4	70.7072	pq	0	77.0419	pq	0	
chr5_pat	70.8733	pq	1	71.8264	pq	0	75.9282	pq	0	
chr6_pat	66.993	pq	2	69.9015	pq	0	72.2183	pq	0	
chr7_pat	62.0297	pq	2	70.4032	pq	0	77.374	pq	0	*
chr8_pat	60.4297	pq	4	69.8121	pq	0	75.4083	pq	0	
chr9_pat	67.4827	pq	2	69.6501	pq	0	77.0346	pq	0	
chr10_pat	68.9832	pq	0	68.9832	pq	0	76.1336	pq	0	
chr11_pat	67.6551	pq	2	71.7729	q	0	76.7442	pq	0	*
chr12_pat	72.6999	pq	0	72.6999	pq	0	79.5193	pq	0	*
chr13_pat	72.1124	pq	0	72.1866	pq	0	75.8415	pq	0	
chr14_pat	63.7976	pq	2	53.5123	pq	0	70.0497	pq	0	*
chrX_mat	67.6872	pq	3	68.6135	pq	0	60.8037	pq	0	
chrY_pat	68.5219	pq	0	68.5219	pq	0	70.8414	pq	0	

Table S2. Assembly quality comparison between T2T-mGirTip1 and ASM1759114v1 and versions of T2T-mGirTip1, related to Figure 6.

	ASM1759114v1	T2T-mGirTip1 (each haploid assembly has chrX, chrY, and chrM)				
		v0.1-Mat	v0.1-Pat	v1.0-Mat	v1.0-Pat	v1.0-Pri
**Genome**						
Num. of total sequences	2,096	117	114	17	17	17
Num. of contigs	2,056	106	103	16	17	17
Num. of scaffolds	40	11	11	1	0	0
Num. of gaps	35,588	22	26	1	0	0
Gap bases (Mbp)	3.46	0.66	0.79	0.01	0.00	0.00
Chromosome assigned (Mbp)	2391.48	2557.63	2572.80	2580.18	2586.41	2580.00
Num. of unplaced sequences	2081	100	97	0	0	0
Unplaced bases (Mbp)	50.00	13.68	14.50	0.00	0.00	0.00
**Scaffold**						
Total length (Gbp)	2.44	2.57	2.59	2.58	2.59	2.58
Minimum length (bp)	504	16,977	16,567	39,421	39,421	16430
Average length (Mbp)	1.16	21.98	22.70	151.78	152.14	151.67
Maximum length (Mbp)	236.01	239.04	238.23	239.04	238.23	238.23
Q1 (bp)	1,732	81,659	76,573	147,919,939	150,165,456	150,165,456
Q2 (bp)	4,000	121,204	123,799	162,004,231	162,115,330	162,115,330
Q3 (bp)	9,026	215,508	212,438	192,562,917	193,438,572	192,562,917
N50 (Mbp) inc. gap	158.23	166.25	161.59	166.64	167.85	166.64
**Gene Contents (inc. unplaced)**						
Single_copy	96.4	98	97.7	98	98	98.2
Duplicated	2.6	1.5	1.7	1.5	1.5	1.3
Fragmented	0.2	0.1	0.1	0.1	0.1	0.1
Missing	0.8	0.4	0.5	0.4	0.4	0.4
Incomplete	4.5	4.4	4.4	4.4	4.4	4.3

Table S3. Non-syntenic region to ASM1759114v1 and its % annotated with RepeatMasker in T2T-mGirTip1.v1.0, related to Figure 6.

Chromosome		Non-syntenic		RepeatMasker (within non-syntenic region)		
Sequence	Length (bp)	Length (bp)	Proportion (%)	Not annotated	Annotated as repeat	% annotated
chr1_mat	227,015,169	16,115,169	7.10	9,639,077	6,476,092	40.19
chr1_pat	236,983,288	26,833,288	11.32	15,791,547	11,041,741	41.15
chr2_mat	239,044,432	4,794,432	2.01	3,187,326	1,607,106	33.52
chr2_pat	238,229,697	3,529,697	1.48	2,334,184	1,195,513	33.87
chr3_mat	192,562,917	5,312,917	2.76	3,069,523	2,243,394	42.23
chr3_pat	193,438,572	6,388,572	3.30	4,144,827	2,243,745	35.12
chr4_mat	212,706,620	3,856,620	1.81	2,867,627	988,993	25.64
chr4_pat	212,213,469	3,613,469	1.70	2,378,193	1,235,276	34.19
chr5_mat	194,161,901	9,261,901	4.77	5,876,868	3,385,033	36.55
chr5_pat	194,525,741	9,825,741	5.05	6,076,867	3,748,874	38.15
chr6_mat	179,095,304	4,145,304	2.31	2,894,204	1,251,100	30.18
chr6_pat	178,487,063	3,137,063	1.76	2,013,543	1,123,520	35.81
chr7_mat	161,659,603	8,009,603	4.95	5,940,532	2,069,071	25.83
chr7_pat	162,115,330	8,865,330	5.47	5,920,298	2,945,032	33.22
chr8_mat	164,781,490	12,481,490	7.57	9,690,476	2,791,014	22.36
chr8_pat	162,492,602	10,442,602	6.43	7,425,260	3,017,342	28.89
chr9_mat	166,642,449	13,992,449	8.40	9,929,912	4,062,537	29.03
chr9_pat	167,853,992	15,553,992	9.27	11,054,381	4,499,611	28.93
chr10_mat	162,004,231	21,754,231	13.43	13,100,616	8,653,615	39.78
chr10_pat	161,569,146	21,019,146	13.01	12,444,848	8,574,298	40.79
chr11_mat	152,365,032	19,365,032	12.71	14,165,614	5,199,418	26.85
chr11_pat	150,900,226	18,100,226	11.99	12,914,101	5,186,125	28.65
chr12_mat	147,919,939	9,419,939	6.37	6,038,324	3,381,615	35.90
chr12_pat	150,165,456	11,565,456	7.70	7,577,535	3,987,921	34.48
chr13_mat	124,363,158	6,763,158	5.44	4,798,110	1,965,048	29.06
chr13_pat	123,819,281	5,969,281	4.82	4,064,365	1,904,916	31.91
chr14_mat	75,183,212	18,800,000	25.01	15,593,527	3,206,473	17.06
chr14_pat	72,936,098	16,300,000	22.35	13,473,890	2,826,110	17.34
chrM	39,421	39,421	100.00	38,664	757	1.92
chrX_mat	154,011,775	42,211,775	27.41	22,747,307	19,464,468	46.11
chrY_pat	26,624,642	26,624,642	100.00	12,692,993	13,931,649	52.33
**Diploid**	**4,985,911,256**	**384,091,946**	**7.70**	**249,884,539**	**134,207,407**	**34.94**
**Primary**	**2,578,355,884**	**220,919,786**	**8.57**	**139,565,385**	**81,354,401**	**36.83**

Table S4. Annotation results for primary and alternative haplotype assemblies of the T2T-mGirTip1v1.0 genome, related to Figure 6.

	**T2T-mGirTip1v1.0**	
**Statistics**	**Primary (Autosomes + XY and MT)**	**Alternative**
**Genes**		
Protein-coding	20,848	19,824
Non-coding	2,785	2,817
Processed pseudogenes	409	346
**Transcripts**		
Protein coding	85,186	82,481
Functionally annotated	83,131	80,465
Long non-coding RNA	2,877	2,911
Processed pseudo	417	351
**Proteins**		
Unique proteins	61,891	59,786

Table S5. BUSCO “mammalia” single-copy ortholog counts for T2T-mGirTip1v1.0 assembly, using 9,226 protein coding genes, related to Figure 6.

		T2T-mGirTip1-v1.0	
		**Primary**	**Alternative**
**Genome**			
	Complete BUSCOs	98.3%	96.1%
	Single-copy	96.7%	94.8%
	Duplicated	1.6%	1.4%
	Fragmented BUSCOs	0.8%	0.8%
	Missing BUSCOs	0.9%	3.1%
**Annotated proteins**			
	Complete BUSCOs	97.9%	95.7%
	Single-copy	30.9%	30.3%
	Duplicated	67.0%	65.5%
	Fragmented BUSCOs	0.4%	0.4%
	Missing BUSCOs	1.7%	3.9%

Table S6. Quality improvement of HG002 assembly using verkko-fillet, related to Figure S19.

	BASE VERKKO	POST CURATION	IMPROVEMENT	
**Assembly stats**				
**num_seqs**	114	86	-28	
**sum_len**	5,989,674,860	5,984,376,403	-5,298,457	
**min_len**	16,570	16,570	0	
**avg_len**	52,541,007.50	69,585,772.10	17,044,764.60	
**max_len**	252,059,510	252,059,489	-21	
**Q1**	152,546	445,976	293,430	
**Q2**	566,348	47,411,991.50	46,845,643.50	
**Q3**	102,878,168	133,994,645	31,116,477	
**sum_gap bases**	1,294,629	1,284,129	-10,500	
**# of gap**	19	16	-3	
**N50**	146,787,937	146,788,106	169	
**GC(%)**	40.81	40.81	0	
**T2T stats**				
**Not T2T**	8	1	-7	
**number of T2T contig (w/ gap)**	6	9	3	
**number of T2T contig (w/o gap)**	32	36	4	
**Assembly accuracy (reported from GQC)**				
**Total errors in alignments of verkko_hg002 to v1.1**	140601	138642	-1959	
**Total substitution errors in alignments of verkko_hg002to v1.1**	35987	35374	-613	
**Total indel errors in alignments of verkko_hg002 to v1.1**	104614	103268	-1346	
**QV improvement**				Notes
**haplotype1_chr1**	70.7944	71.0941	0.2997	
**haplotype2_chr1**	69.8815	69.3192	-0.5623	
**haplotype1_chr2**	68.8328	68.9221	0.0893	
**haplotype2_chr2**	70.4436	71.3856	0.942	
**haplotype1_chr3**	56.6618	70.6238	13.962	2 gaps filled
**haplotype2_chr3**	72.7584	72.4058	-0.3526	
**haplotype1_chr4**	68.6813	69.1882	0.5069	
**haplotype2_chr4**	66.0572	65.8528	-0.2044	
**haplotype1_chr5**	69.6339	69.0124	-0.6215	
**haplotype2_chr5**	69.9856	70.1171	0.1315	
**haplotype1_chr6**	69.6209	69.7479	0.127	
**haplotype2_chr6**	71.4718	71.811	0.3392	
**haplotype1_chr7**	68.5921	68.9666	0.3745	

**haplotype2_chr7**	63.4338	63.3916	-0.0422	
**haplotype1_chr8**	62.2833	62.3141	0.0308	
**haplotype2_chr8**	70.6587	70.8634	0.2047	
**haplotype1_chr9**	67.4066	67.7699	0.3633	
**haplotype2_chr9**	61.9662	64.2767	2.3105	trimmed
**haplotype1_chr10**	68.2053	67.7764	-0.4289	
**haplotype2_chr10**	65.2009	66.0587	0.8578	
**haplotype1_chr11**	69.8196	69.7303	-0.0893	
**haplotype2_chr11**	69.4986	69.3761	-0.1225	trimmed
**haplotype1_chr12**	68.0219	67.9494	-0.0725	
**haplotype2_chr12**	69.553	69.2692	-0.2838	
**haplotype1_chr13**	60.1238	64.0838	3.96	trimmed
**haplotype2_chr13**	49.6105	56.5745	6.964	trimmed
**haplotype1_chr14**	58.351	67.7445	9.3935	trimmed
**haplotype2_chr14**	68.4302	69.0128	0.5826	
**haplotype1_chr15**	59.2623	59.2384	-0.0239	
**haplotype2_chr15**	60.4685	60.7335	0.265	trimmed
**haplotype1_chr16**	67.8953	67.8953	0	
**haplotype2_chr16**	65.4308	65.6286	0.1978	
**haplotype1_chr17**	69.2167	65.7488	-3.4679	1 gap filled
**haplotype2_chr17**	66.7129	67.6823	0.9694	
**haplotype1_chr18**	72.8813	72.8813	0	
**haplotype2_chr18**	69.6249	69.6249	0	
**haplotype1_chr19**	58.9368	58.9637	0.0269	
**haplotype2_chr19**	61.9184	61.8445	-0.0739	
**haplotype1_chr20**	57.49	57.4865	-0.0035	
**haplotype2_chr20**	65.7643	65.1269	-0.6374	trimmed
**haplotype1_chr21**	64.5066	64.5039	-0.0027	trimmed
**haplotype2_chr21**	63.1655	63.1654	-0.0001	
**haplotype1_chr22**	65.7177	66.2467	0.529	
**haplotype2_chr22**	66.9276	65.8611	-1.0665	
**haplotype2_chrX**	66.5713	66.5713	0	
**haplotype1_chrY**	64.4589	66.0539	1.595	

Table S7. The computing resources required for each step of the essential key features in the Verkko fillet, related to Section [Verkko-Fillet improves a benchmark human genome assembly].

Step	Elapsed	CPU	maxMem	maxVmem	%CPU	AvgCPU	Storage	External Tools used in each step	Subdirectory	Details
T2T status check	0.03	0.01	0.22	0.81	16.83	0.006	20G	seqtk, samtools, bedtools	stats	
QV calculation	0.58	7.60	21.13	22.69	646.79	3.80	16K	meryl, merqury	kmer	
chromosome assignment	0.10	0.50	4.30	6.75	251.95	0.25	5K	seqtk, mashmap, gfacpp	chromosome_assignment	
Align ONT reads on the graph	0.3494	13.01	12.53	18.53	1862.01	6.50	7.2G	GraphAligner	graphAlignment	Align 53,000 reads onto the graph (192 batches)
writing verkko-fillet obj							1.6G	NA (python pickle)		For storage estimation, this job also does not require significant computing resources and takes approximately 1 minute to complete.
Running CNS steps	124.62	13.64	238.48	385468189	5.47	6.82	700G	verkko		

* The majority of other features and functions used in Jupyter or Python do not require significant computing resources or storage, as they mainly involve dataframe management and use data already existing in the Verkko output folder. Therefore, they are excluded from this table.
